# Tumor mutation burden-related long non-coding RNAs is predictor for prognosis and immune response in pancreatic cancer

**DOI:** 10.1186/s12876-022-02535-z

**Published:** 2022-11-29

**Authors:** Chunjing Wang, Zhen Wang, Yue Zhao, Ruichun Jia

**Affiliations:** 1 Department of General Surgery, The Second Affiliated Hospital of Harbin Medical University, Harbin, China; 2 Department of Blood Transfusion, The Second Affiliated Hospital of Harbin Medical University, 150001 Harbin, China

**Keywords:** Pancreatic cancer, Tumor mutation burden, Long non-coding RNA, Immune infiltration, Mechanism

## Abstract

**Background:**

Pancreatic cancer is one of the most common malignant tumors with extremely poor prognosis. It is urgent to identify promising prognostic biomarkers for pancreatic cancer.

**Methods:**

A total of 266 patients with pancreatic adenocarcinoma (PAAD) in the Cancer Genome Atlas (TCGA)-PAAD cohort and the PACA-AU cohort were enrolled in this study. Firstly, prognostic tumor mutation burden (TMB)-related long non-coding RNAs (lncRNAs) were identified by DESeq2 and univariate analysis in the TCGA-PAAD cohort. And then, the TCGA-PAAD cohort was randomized into the training set and the testing set. Least absolute shrinkage and selection operator (LASSO) was used to construct the model in the training set. The testing set, the TCGA-PAAD cohort and the PACA-AU cohort was used as validation. The model was evaluated by multiple methods. Finally, functional analysis and immune status analysis were applied to explore the potential mechanism of our model.

**Results:**

A prognostic model based on fourteen TMB-related lncRNAs was established in PAAD. Patients with High risk score was associated with worse prognosis compared to those with low risk score in all four datasets. Besides, the model had great performance in the prediction of 5-year overall survival in four datasets. Multivariate analysis also indicated that the risk score based on our model was independent prognostic factor in PAAD. Additionally, our model had the best predictive efficiency in PAAD compared to typical features and other three published models. And then, our findings also showed that high risk score was also associated with high TMB, microsatellite instability (MSI) and homologous recombination deficiency (HRD) score. Finally, we indicated that high risk score was related to low immune score and less infiltration of immune cells in PAAD.

**Conclusion:**

we established a 14 TMB-related lncRNAs prognostic model in PAAD and the model had excellent performance in the prediction of prognosis in PAAD. Our findings provided new strategy for risk stratification and new clues for precision treatment in PAAD.

**Supplementary Information:**

The online version contains supplementary material available at 10.1186/s12876-022-02535-z.

## Introduction

Pancreatic cancer is one of the most common fatal malignancies and also known as the “king of cancers” [1]. Patients with pancreatic cancer have very poor outcomes and its 5-year survival rate is only 8% [2]. The morbidity and mortality of pancreatic cancer is continuous increasing [3]. Clinically, the treatment that offers a potential cure of pancreatic cancer is surgical resection followed by the adjuvant chemotherapy. However, the prognosis of patients with pancreatic cancer is still very poor because of the difficult of early diagnosis and the exhibition of a remarkable resistance to therapies. Recently, neoadjuvant therapy has been developed and several trials have suggested the beneficial effects of it for pancreatic cancer [4]. Besides, targeted therapies and immune therapeutic have become the newer trends in the treatment of pancreatic cancer. Some highly mutated genes have been used as targets in the treatment of pancreatic cancer [5], and some targeted-drug have been approved to be used in clinical treatment, including Erlotinib (EGFR inhibitor) [6], Larotrectinib (NTRK inhibitor), and olaparib (PARP inhibitor) [7]. However, immune checkpoint blockade has limited efficacy in pancreatic cancer, which may be partly attributable to the unique immunosuppressive tumor microenvironment of pancreatic cancer [8–10]. Therefore, researchers are focusing more on combination therapy [11]. However, lack of effective risk stratification is a challenge for personalized therapy.

Tumor mutation murden (TMB) has become an independent biomarker for predicting the response to immune checkpoint inhibitors (ICIs) [12, 13]. Patients with high TMB are more sensitive to immunotherapy than those with low TMB. Several studies have demonstrated that TMB played crucial role in immune microenvironment of pancreatic cancer and could be the potential biomarker for immunotherapy in pancreatic cancer [14–16]. Long non-coding RNA (lncRNA) is a module of RNA that has no or limited capacity of protein coding and its length is longer than 200nt. Although lncRNA has been considered as the junk in the genome, many biological functions of lncRNA have been disclosed [17]. lncRNA plays important role in the regulation of many biological processes in health cells, thus, the role of lncRNA in tumor cells arouse the interest of researchers [18]. In pancreatic cancer, numerous lncRNA has been reported to participate in the regulation of tumorigenesis [19–22], apoptosis [23, 24], metastasis [25–27], and chemoresistance [28, 29]. To the best of our knowledge, the role of TMB-related lncRNA in the prognosis of pancreatic cancer has not been investigated yet. In this study, we established a prognostic model in pancreatic cancer based on the TMB-related lncRNA. And then, we compared our model with other published models to test the predictive efficiency of our model. Finally, the relationship between our model and immune infiltration in pancreatic cancer was discussed. This study was looking forward to provide new thought for biomarker searching and precision treatment in pancreatic cancer.

## Methods

### Data collection

The transcriptome profiles, somatic mutation data, and complete clinical information of 176 patients with pancreatic adenocarcinoma (PAAD) were downloaded from The Cancer Genome Atlas (TCGA) database. As validation, PACA-AU cohort with 90 PAAD patients was also enrolled in this study. The transcriptome profiles and survival data of the PACA-AU cohort were downloaded from the International Cancer Genome Consortium (ICGC) database.

### Identification of prognostic TMB-related lncRNAs

TMB is a measure of the total number of mutations per megabyte of tumor tissue. The TMB score of each patient in the TCGA-PAAD cohort was calculated by the “maftools” R package. According to the median value of TMB score, patients in the TCGA-PAAD cohort were divided into high-TMB and low-TMB two groups. DESeq2 [30] was used to perform the differentially expressed genes analysis between high-TMB and low-TMB groups, and the lncRNAs that satisfied the following criteria: Fold Change (FC) > 1.5 and p-value < 0.05 were selected as differentially expressed lncRNA (DElncRNA). Univariate analysis was used to evaluate the prognostic significance of DElncRNAs in the TCGA-PAAD cohort.

### Model construction

176 patients in the TCGA-PAAD cohort were divided into two sets (the training set and the testing set) randomly and equally and the clinical information of the training set, the testing set and the TCGA-PAAD cohort was shown in Supplementary Table 1. Least absolute shrinkage and selection operator (LASSO) Cox regression was used to construct a model based on the prognostic TMB-related lncRNAs in the training set by using “glmnet” R package. The risk score of each patient was calculated by a unified formula as following:

$${\sum }_{i}^{n}coefi*expri$$

In the formula, “coefi” represents the coefficient of the selected prognostic TMB-related lncRNA, and “expri” represents the expression level of the selected prognostic TMB-related lncRNA.

### Model evaluation and validation

The training set was used as the evaluation set while the testing set and the entire TCGA-PAAD cohort were used as the internal validation sets. Besides, the PACA-AU cohort was used as the external validation set. Each set was separated into high-risk and low-risk groups based on the median value of the risk score. To test the prognostic value of the model, Kaplan-Meier survival analysis was used to draw the survival curve and log-rank was used to evaluate the significant difference of prognosis between high-risk and low-risk groups. To test the accuracy of the model, receiver operating characteristic (ROC) curve of 1-year, 3-year and 5-year survival was performed and the value of Area Under Curve (AUC) was used to measure the accuracy of the model in each set. Univariate analysis and multivariate analysis were performed to test the independence of the model in the TCGA-PAAD cohort. Several clinical characteristics were enrolled in the analysis, including age, gender, tumor stage, TMN stage, grade, family history of cancer, history of chronic pancreatitis, history of diabetes, history of alcohol exposure, and history of radiation therapy.

### Model comparison

Many clinicopathological features have been used as the indexes in risk stratification clinically. Therefore, we first compared the predictability of our model and some clinical features including age, gender, tumor stage, TMN stage, grade, and history of radiation therapy by using ROC analysis. In recent years, many novel molecular models have been developed. We also compared the predictive efficiency of our model and other three published models by using ROC analysis and concordance index (C-index).

### Functional enrichment analysis

To further demonstrate the potential mechanism of our model, comparison of the differentially expressed genes between high-risk and low-risk groups in the TCGA-PAAD cohort was performed by using DESeq2 [30] with the criteria of FC > 1.5 and p-value < 0.05. Differentially expressed genes (DEGs) were used to perform the functional enrichment analysis by using Kyoto Encyclopedia of Genes and Genomes (KEGG) pathway analysis [31]. “clusterProfiler” R package [32] was used to perform the KEGG analysis.

### Immune status analysis

We obtained the gene sets of 28 immune cell types [33]. The ssGSEA was performed to explore the different infiltration degrees of immune cell types between high-risk and low-risk groups in the TCGA-PAAD cohort by using “GSVA” R package [34, 35].

### Statistical analysis

All the statistical analysis and visualization were performed with the R version 4.0.2 (Institute for Statistics and Mathematics, Vienna, Austria 4). Two-tailed p 0.05 was used to determine statistical significance. All methods were carried out in accordance with relevant guidelines and regulations.

## Results

### Identification of prognostic TMB-related lncRNAs and model construction

**Fig. 1:**
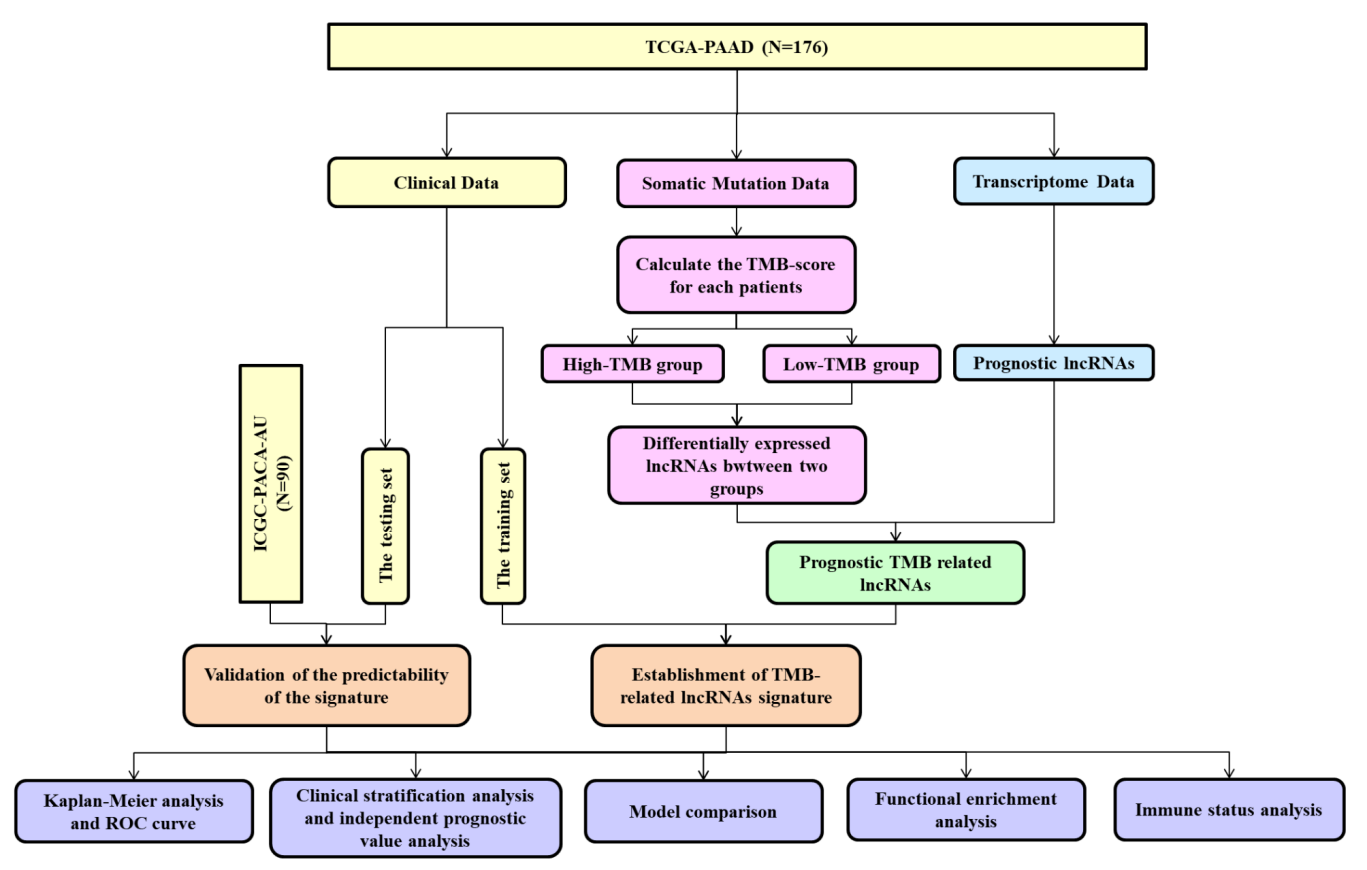
Research roadmap of this study

**Fig. 2:**
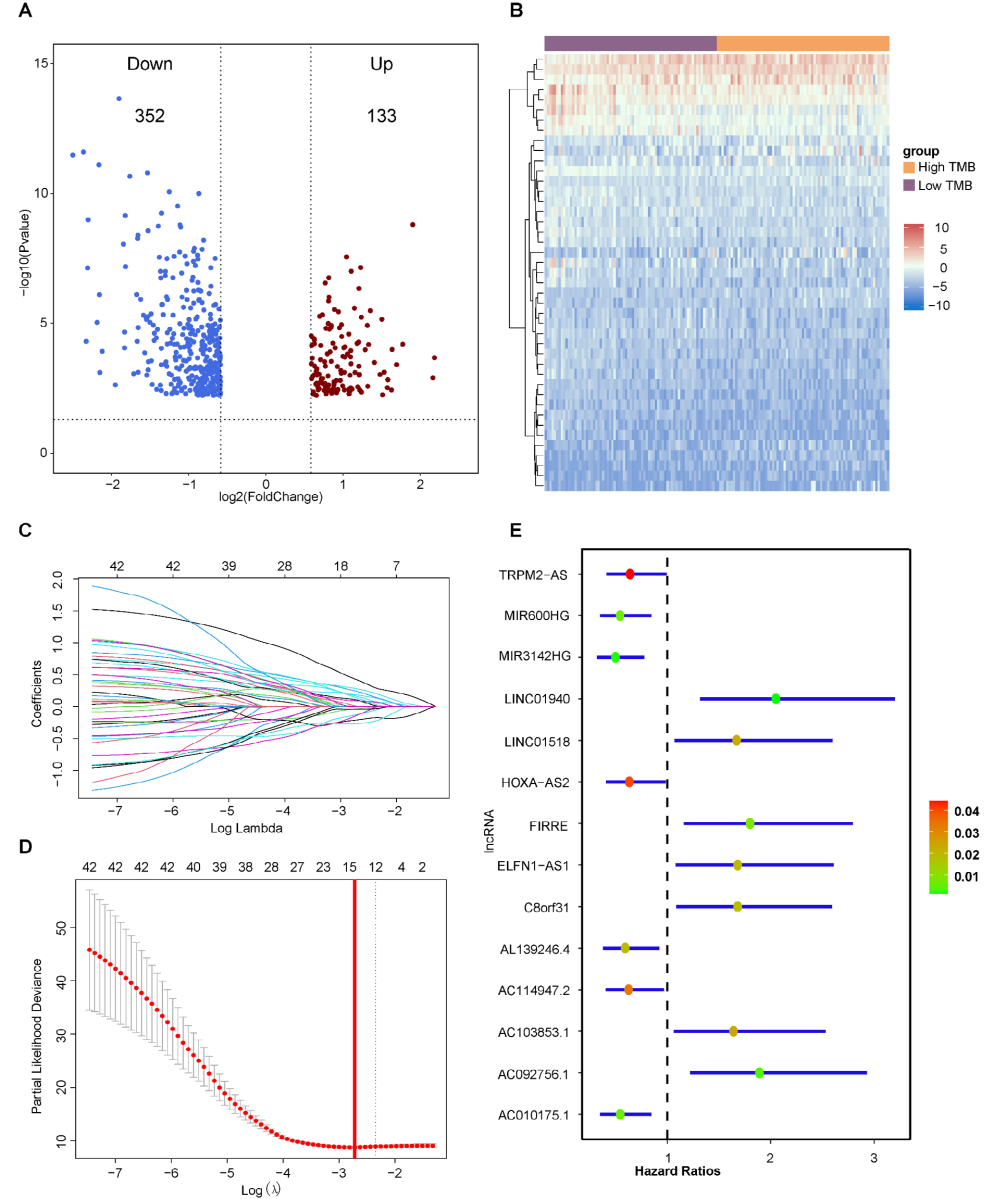
Identification of prognostic TMB-related lncRNAs and model construction. (A) Volcano plot of differentially expressed lncRNAs between high-TMB and low-TMB groups. Red dot represents up-regulated lncRNA and blue dot represents down-regulated lncRNA. (B) heatmap of the expression of 43 prognostic TMB-related lncRNAs in high-TMB and low-TMB groups. (C) The LASSO coefficient profile of 43 prognostic TMB-related lncRNAs and perpendicular imaginary lines were drawn at the value chosen by 10-fold cross-validation. (D) The tuning parameters (log l) of OS-related proteins were selected to cross-verify the error curve. According to the minimal criterion and 1-se criterion, perpendicular imaginary lines were drawn at the optimal value. (E) Multivariate analysis of fourteen selected genes in the TCGA-PAAD cohort

As shown in Fig. 1, Patients in the TCGA-PAAD cohort were divided into high-TMB and low-TMB groups based on the median value of the TMB score. 352 down-regulated lncRNAs and 133 up-regulated lncRNAs were identified as TMB-related lncRNAs (Fig. 2A) and the details were shown in Supplementary Table  2. And then, univariate analysis showed that 43 TMB-related lncRNAs were selected as candidates for model construction (Supplementary Table 3) and the expression pattern of 43 prognostic TMB-related lncRNAs between high-TMB and low-TMB groups was shown in Fig. 2B. Finally, a 14-lncRNA prognostic model was constructed by LASSO Cox method (Fig. 2C-2D) in the training set, including TRPM2-AS, MIR600HG, MIR3142HG, LINC01940, LINC01518, HOXA-AS2, FIRRE, ELFN1-AS1, C8orf31, AL139246.4, AC114947.2, AC103853.1, AC092756.1, and AC010175.1 (Supplementary Table 4). In addition, multivariate analysis was performed and the result indicated that these 14 lncRNAs were independent factor on the prognosis in the TCGA-PAAD cohort (p < 0.05), seven of which (TRPM2-AS, MIR600HG, MIR3142HG, HOXA-AS2, AL139246.4, AC114947.2, and AC010175.1) were protective factors with hazard ratios (HR) < 1 and the rest of which were poor factors with HR > 1 (Fig. 2E).

### Evaluation and validation of the 14-lncRNA prognostic model

**Fig. 3:**
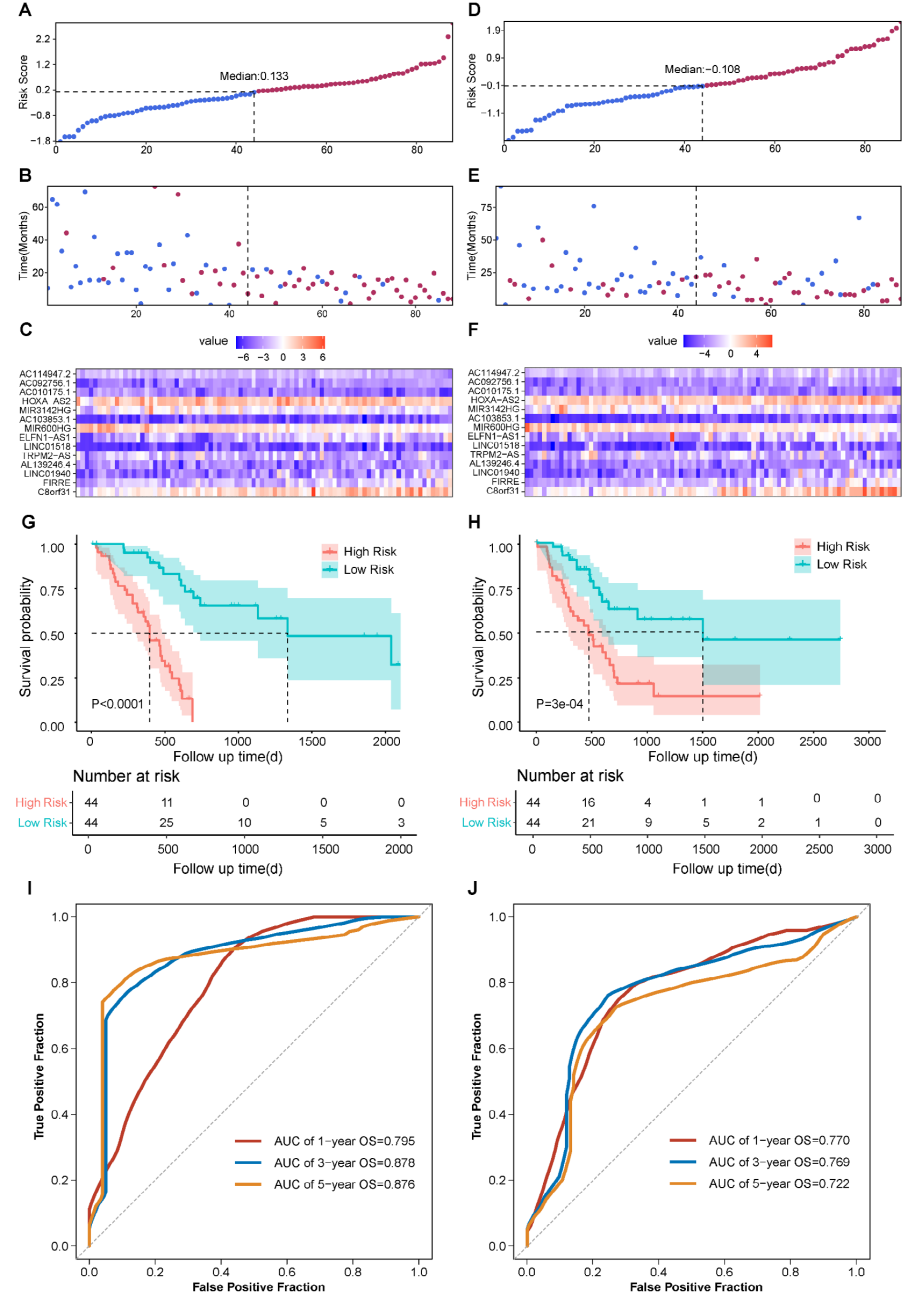
Prognosis analysis of the 14-lncRNAs prognostic model in the training set and the testing set. Distribution of risk score based on the sixteen-gene model in the training set (A) and the testing set (D). Patterns of survival status and survival time of each patient in the training set (B) and the testing set (E). Expression pattern of sixteen genes of each patient in the training set (C) and the testing set (F). Kaplan-Meier survival curves of the OS of patients in the high- and low-risk groups in the training set (G) and the testing set (H). ROC curve of 1-year, 3-year, and 5-year overall survival in the training set (I) and the testing set (J)

**Fig. 4:**
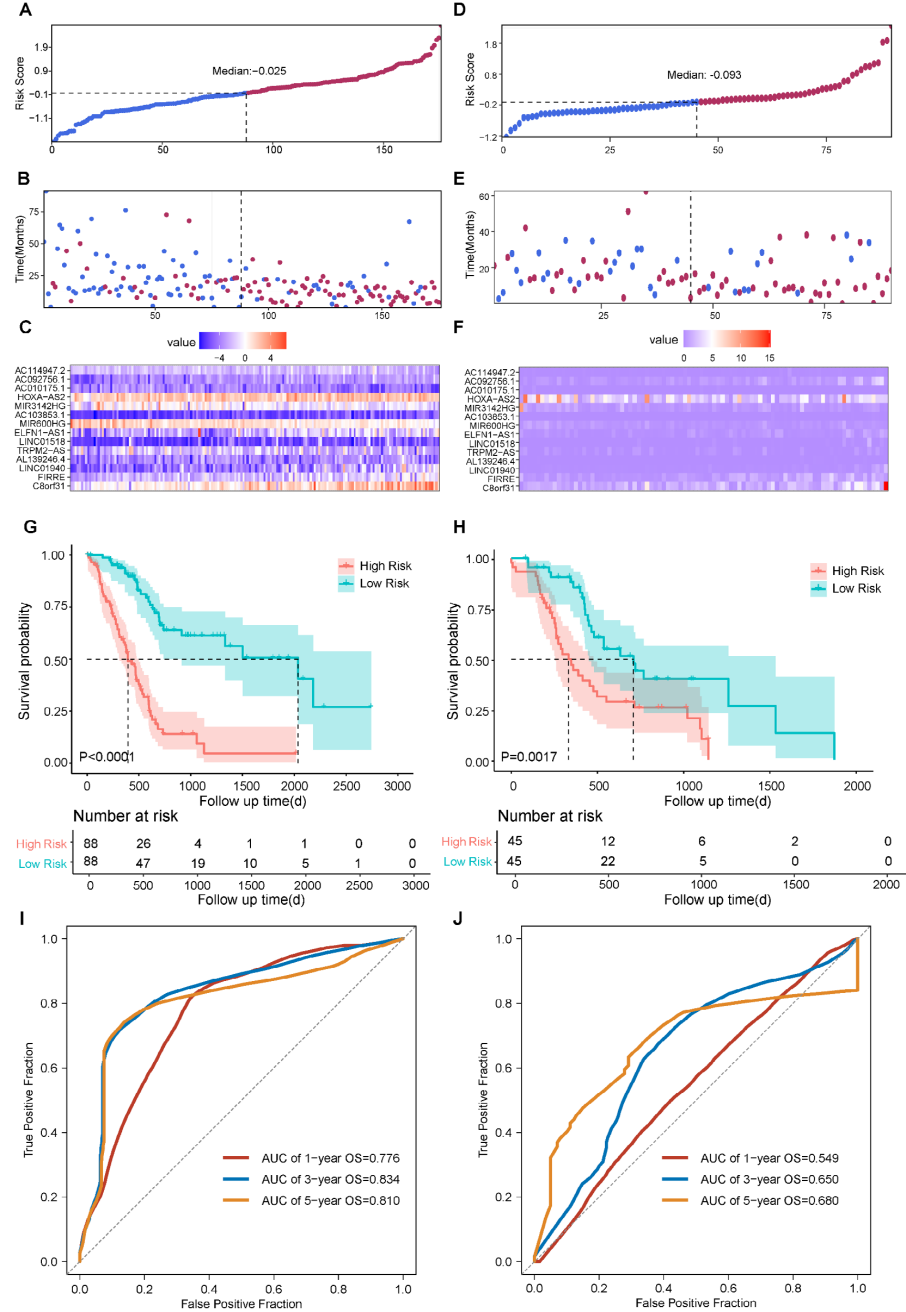
Prognosis analysis of the 14-lncRNAs prognostic model in the TCGA-PAAD cohort and PACA-AU cohort. Distribution of risk score based on the sixteen-gene model in the TCGA-PAAD cohort (A) and the PACA-AU cohort (D). Patterns of survival status and survival time of each patient in the TCGA-PAAD cohort (B) and the PACA-AU cohort (E). Expression pattern of sixteen genes of each patient in the TCGA-PAAD cohort (C) and the PACA-AU cohort (F). Kaplan-Meier survival curves of the OS of patients in the high- and low-risk groups in the TCGA-PAAD cohort (G) and the PACA-AU cohort (H). ROC curve of 1-year, 3-year, and 5-year overall survival in the TCGA-PAAD cohort (I) and the PACA-AU cohort (J)

The risk scores of each patient were calculated and the training set was divided into high-risk and low-risk groups based on the median value of risk scores (Fig. 3A). The status of each patient and the expression pattern of 14 lncRNAs of each patient were shown in Fig. 3B-3C respectively. Comparison of survival curve between high-risk and low-risk groups suggested that patients with high risk score had significantly shorter overall survival (OS) than those with low risk score (Fig. 3G, p < 0.0001). Besides, the AUC of 1-year, 3-year, and 5-year OS was 0.795, 0.878, and 0.876, respectively (Fig. 3I). As validation, the same process was performed in the testing set (Fig. 3D-3F), the entire TCGA-PAAD cohort (Fig. 4A-4C), and the PACA-AU cohort (Fig. 4D-4F). Similarly, the high-risk group was associated with worse prognosis compared to the low-risk group in the testing set (Fig. 3H, p = 3e-04), the entire TCGA-PAAD cohort (Fig. 4G, p < 0.0001), and the PACA-AU cohort (Fig. 4H, p = 0.0017). Additionally, we also evaluated the AUC of 1-year, 3-year, and 5-year OS in the testing set (Fig. 3J), the entire TCGA-PAAD cohort (Fig. 4I), and the PACA-AU cohort (Fig. 4J). It is also well-known that 90% of PAAD were classified as pancreatic ductal adenocarcinoma (PDAC). Thus, we further evaluate the prognostic value of our signature only in the PDAC patients of the PACA-AU cohort. The result showed that patients with high risk score had inferior outcomes compared to those with low risk score, but the difference was not significant (Supplementary Fig. 1). The possible reason for this result is the small sample size of PDAC (n = 40).

### Association of the 14-lncRNA prognostic model and clinicopathological characteristics

**Fig. 5:**
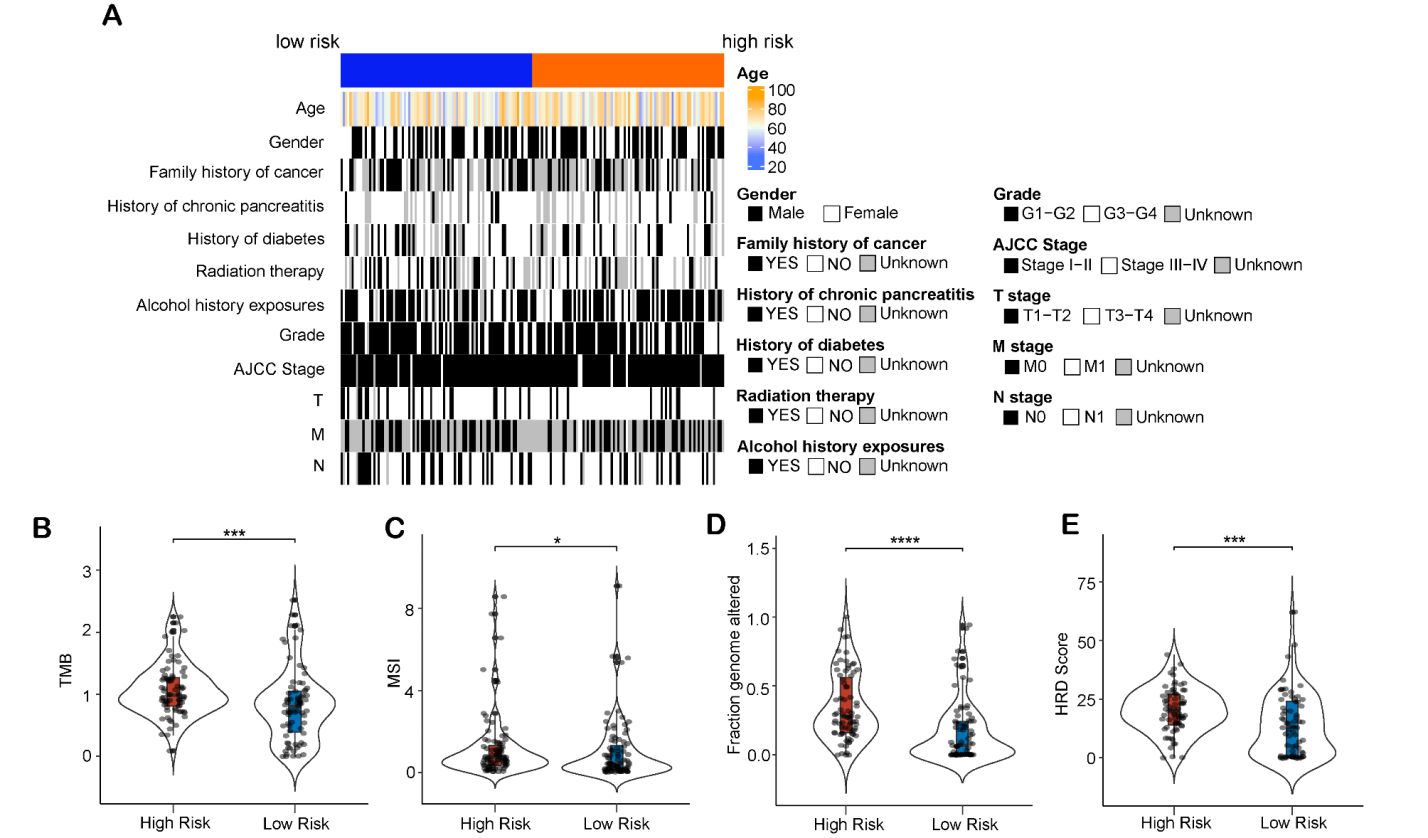
Association of the 14-lncRNAs prognostic model and clinicopathological features. (A) Comparison of clinical features between high-risk and low-risk groups in the TCGA-PAAD cohort. (B) Comparison of tumor mutation burden (TMB) between high-risk and low-risk groups in the TCGA-PAAD cohort. (C) Comparison of microsatellite instability (MSI) between high-risk and low-risk groups in the TCGA-PAAD cohort. (D) Comparison of fraction genome altered (FGA) between high-risk and low-risk groups in the TCGA-PAAD cohort. (E) Comparison of homologous recombination deficiency (HRD)-score between high-risk and low-risk groups in the TCGA-PAAD cohort. “*” represents p < 0.05, “***” represents p < 0.001, “****” represents p < 0.0001

We compared the clinicopathological characteristics of high-risk and low-risk groups in the TCGA-PAAD cohort. As shown in Fig. 5A, the T stage was significantly associated with the risk score: high risk group had more patients with T3-T4 while patients with T1-T2 were more clustered in low risk group. No significant difference was found in other clinicopathological characteristics between high-risk and low-risk groups. And then, the differences of TMB, microsatellite instability (MSI), fraction genome altered (FGA), homologous recombination deficiency (HRD)-score, and immune score between high-risk and low-risk groups were also compared. Notably, the risk score was positively correlated with the TMB (Fig. 5B, p < 0.001), MSI (Fig. 5C, p < 0.05), FGA (Fig. 5D, p < 0.0001) and HRD-score (Fig. 5E, p < 0.001).

**Fig. 6:**
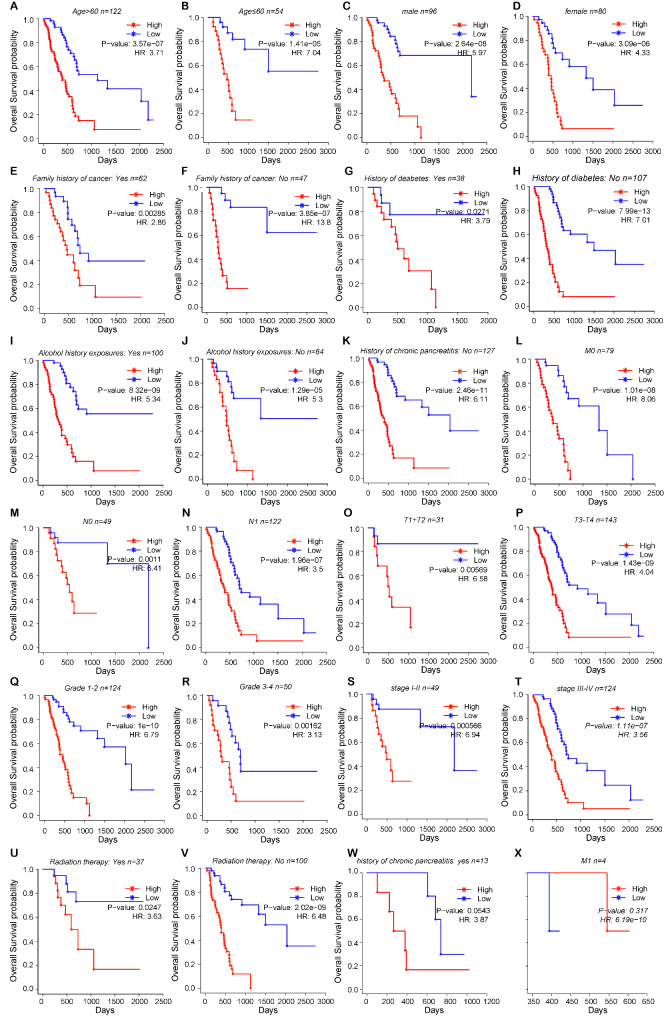
Prognosis analysis of PAAD patients in different subgroups

**Fig. 7:**
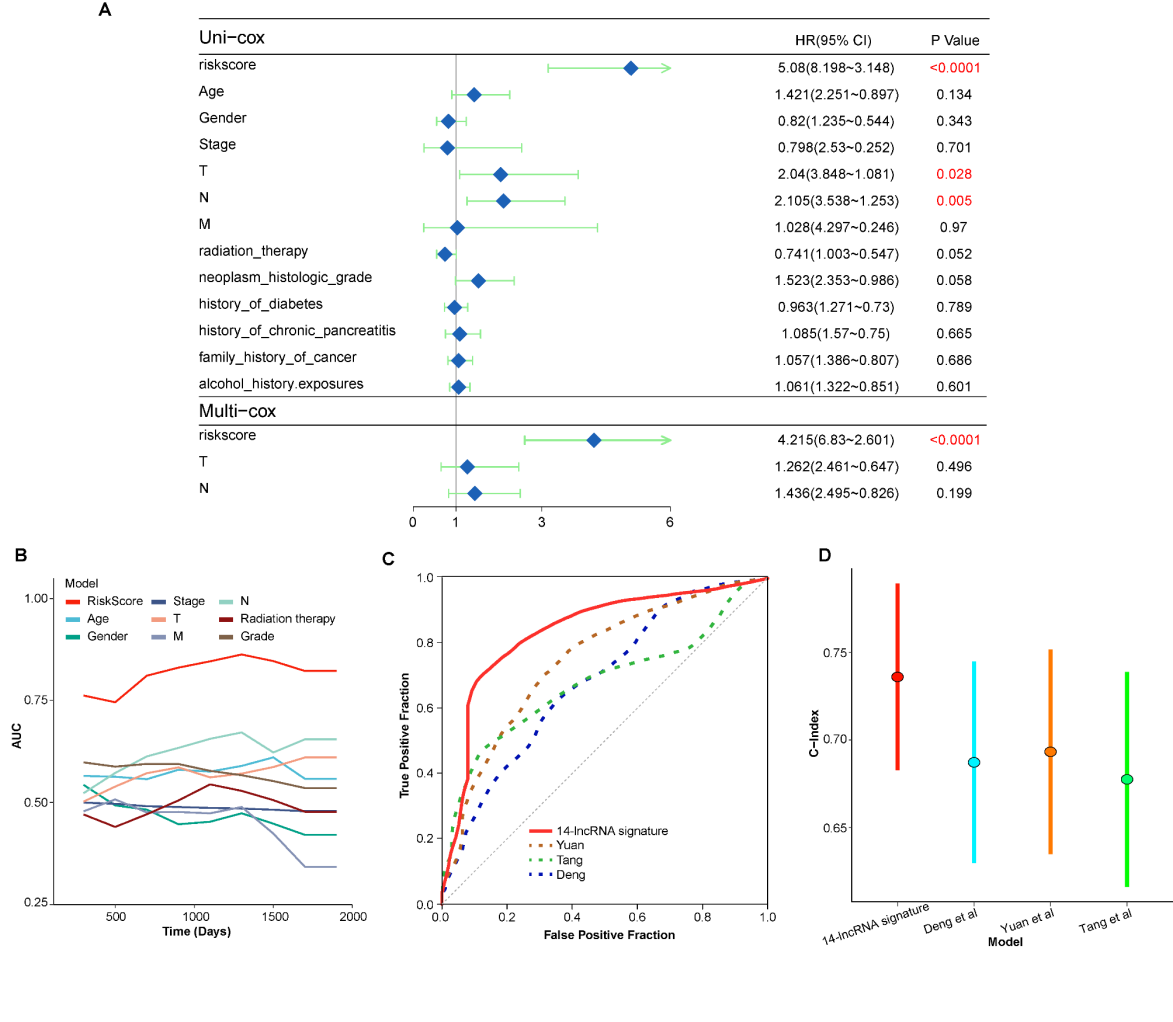
Multivariate analysis and models comparison. (A) Univariate analysis and multivariate analysis of the risk score based on the 14-lncRNA prognostic model and other clinical features. (B) Comparison of AUC value of the 14-lncRNA prognostic model and other clinical features. (C) Comparison of ROC curve of 3-year overall survival between the 14-lncRNA prognostic model and other three published models. (D) Comparison of C-index between the 14-lncRNA prognostic model and other three published models

To further investigate whether the prognostic value of the 14-lncRNA prognostic model can be impacted by the clinicopathological characteristics, patients in the TCGA-PAAD cohort were grouped by the clinicopathological characteristics including age (≤ 60 years and > 60 years), gender (male and female), family history of cancer (yes and no), history of diabetes (yes and no), history of alcohol exposure (yes and no), history of chronic pancreatitis (yes and no), M stage (M0 and M1), N stage (N0 and N1), T stage (T1 + T2 and T3 + T4), grade (grade 1–2 and grade 3–4), tumor stage (stage I-II and stage III-IV), history of radiation therapy (yes and no). Each subgroup was further divided into high-risk and low-risk groups based on the median value of risk score. Except for patients with M1 and patients with history of chronic pancreatitis, high-risk group was associated with inferior prognosis compared to low-risk group in every subgroup (Fig. 6). The possible reason for this result was the small number of patients in the subgroup of patients with M1 (n = 4) and patients with history of chronic pancreatitis (n = 13). Besides, all the clinicopathological characteristics were enrolled in the univariate analysis, and the result showed that the risk score was also the poor prognostic factor in the TCGA-PAAD cohort (Fig. 7A, p < 0.0001) besides T stage (Fig. 7A, p = 0.028) and N stage (Fig. 7A, p = 0.005). And then, we enrolled these three prognostic factors in the multivariate analysis. Interestingly, the risk score was also the only independent factor on the prognosis in the TCGA-PAAD cohort (Fig. 7A, p < 0.0001).

### Models comparison

Firstly, we compared the value of AUC between our model and some clinicopathological features including age, gender, tumor stage, grade, TMN stage and history of radiation therapy. Unsurprisingly, the AUC of our model was always the largest comparing with other features (Fig. 7B). And then, comparison of our model and three recently published models was performed, including the m6A-related lncRNA prognostic model reported by Yuan et al. [36], the TMB-related genes prognostic model reported by Tang et al. [16], and the autophagy-related lncRNA prognostic model reported by Deng et al. [37]. As shown in Fig. 7C, our 14-lncRNA prognostic model had the best predictive efficiency of 3-year OS in the TCGA-PAAD cohort compared with other three published models. Besides, our model had the biggest value of C-index compared to other three published models (Fig. 7D), which further indicated that our model had excellent performance in the prediction of prognosis in PAAD.

### Exploration of potential mechanism of the 14-lncRNA prognostic model in PAAD.

**Fig. 8:**
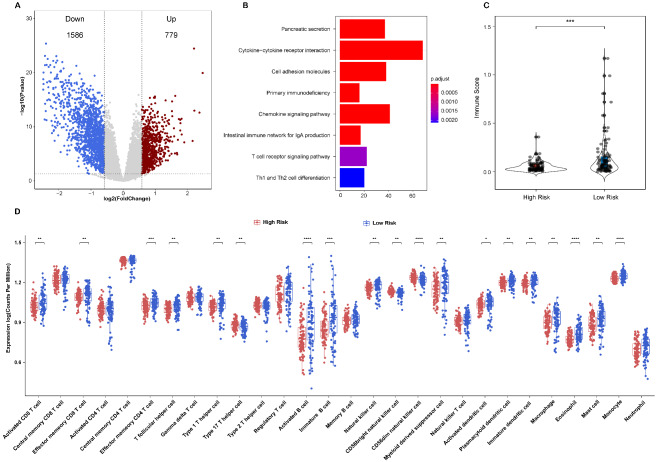
Exploration of potential mechanism of the 14-lncRNA prognostic model. (A) Volcano plot of differentially expressed genes between high-risk and low-risk groups in the TCGA-PAAD cohort. Red dot represents up-regulated genes, blue dot represents down-regulated genes, and grey dot represents not differentially expressed genes. (B) KEGG pathway enrichment analysis of differentially expressed genes. (C) Comparison of immune score between high-risk and low-risk groups in the TCGA-PAAD cohort. (D) Immune status analysis of 28 types of immune cells between high-risk and low-risk groups in the TCGA-PAAD cohort

To further explore the potential mechanism of our model, differentially expressed genes (DEGs) analysis was performed between high-risk and low-risk groups in the TCGA-PAAD cohort. 1586 down-regulated genes and 779 up-regulated genes were selected as DEGs (Fig. 8A, Supplementary Table 5). And then, functional enrichment analysis was performed based on the DEGs. KEGG analysis showed that the DEGs were majorly enriched in pancreatic secretion pathway and many immune-related signaling pathways including cytokine-cytokine receptor interaction, cell adhesion molecules, primary immunodeficiency, chemokine signaling pathway, and intestinal immune network for IgA production, T cell receptor signaling pathway, and Th1 and Th2 cell differentiation (Fig. 8B), which suggested that our model might be associated with the immunoregulation of PAAD. Therefore, we compared the immune score and immune infiltration of 28 immune cells between high-risk and low-risk groups in the TCGA-PAAD cohort. Notably, patients with high risk score had significantly lower immune score (Fig. 8C, p < 0.001). Besides, the infiltrations of 19 types of immune cells were significantly higher in the low-risk groups compared to the high-risk group, including some immune cells that played crucial role in tumor immunity such as CD8 T cells, CD4 T cells, natural killer cells and B cells (Fig. 8D).

## Discussion

TMB has been used as the predictor for immune checkpoint inhibitors in tumors [12]. Recent studies have been illustrated that lncRNAs could be the prognostic biomarker for many types of cancer including pancreatic cancer [38]. To the best of our knowledge, a prognostic model based on the TMB-related lncRNAs in pancreatic cancer has not been reported yet. In this study, two datasets: TCGA-PAAD and PACA-AU were collected. The TMB of 176 patients in the TCGA-PAAD cohort was calculated and the cohort was divided into high-TMB and low-TMB groups based on the median value of TMB. And then, 43 lncRNAs were identified as prognostic TMB-related lncRNAs by using differentially expressed genes analysis and univariate analysis. Subsequently, the TCGA-PAAD cohort was randomized into the training set and the testing set. LASSO was used to construct a 14-lncRNAs prognostic model in the training set. The training set was used as the evaluation set. The testing set and the entire TCGA-PAAD cohort were used as the internal validation sets, and the PACA-AU cohort was used as the external validation set. The risk scores of each patient were calculated and the patients of each set were divided into high-risk and low-risk groups based on the median value of risk scores respectively. Prognosis analysis suggested that patients with high risk score was associated with poor prognosis in all four sets (p < 0.01). Besides, ROC curve also indicated that the predictability of the 14-lncRNA prognostic model was great in the 1-year, 3-year, and 5-year OS. Additionally, univariate analysis and multivariate analysis of the risk score and other clinical features were indicated that the risk score based on the 14-lncRNA prognostic model was the independent protective factor on the prognosis of PAAD. To further evaluate the accuracy of the 14-lncRNA prognostic model, we compared the AUC value of the 14-lncRNA prognostic model and some clinical factors and the result suggested that our model had the best accuracy of prognosis prediction compared to other clinical features. Besides, we also compared the AUC of 3-year OS and the value of C-index of the 14-lncRNA prognostic model and other three published models. Unsurprisingly, our model also had the best predictive efficiency compared to other published models.

To further investigate the relationship of the 14-lncRNA prognostic model and some hallmark of tumorigenesis including TMB, MSI, FGA, and HRD-score, we indicated that high risk score was associated with high TMB, MSI, FGA and HRD-score. TMB represents the number of mutations per megabase (Mut/Mb) of DNA that were sequenced in a specific cancer. Nowadays, numerous studies have illuminated that cancer patients with high TMB could benefit from immunotherapy [12, 39]. Besides, MSI and FGA are hallmarks of genomic instability which has been recognized as one of the drivers of carcinogenesis [40, 41]. Many researchers have demonstrated that high genomic instability could be the basis for a tumor’s sensitivity to DNA-damaging therapies [42]. Our findings suggested that the 14 TMB-related lncRNAs prognostic model had the potential to be the indicator for immunotherapy and DNA-damaging therapies response, which provided new thought for precision treatment in PAAD.

To further explore the potential mechanism the 14-lncRNA prognostic model in PAAD, differentially expressed genes analysis was performed to identify the DEGs between high-risk and low-risk groups in the TCGA-PAAD cohort. And then, KEGG pathway analysis indicated that the 14-lncRNA prognostic model might participate in many immune-related pathways. In addition, we compared the immune score and the immune infiltration of 28 types of immune cells between high-risk and low-risk groups in TCGA-PAAD cohort. The results showed that patients with high risk score had significantly lower immune score and less immune infiltration of 19 types of immune cells. Our findings indicated that high risk score might be associated with inactivation of immune-related pathways and the inefficient infiltration of immune cells, which resulted to poor prognosis in PAAD.

In conclusion, our findings provided new strategy for risk stratification in PAAD and offered new thought for prognostic biomarker and precision therapy in PAAD. However, there are some limitations in this study. For example, our study was more suitable for retrospective analysis and the established model was still not clinical actionable. This study tentative explored that the 14-TMB-related lncRNA prognostic model might participate in the immunoregulation of PAAD. However, external experiments would be advantaged for further investigation. In the future, we will attempt to overcome these shortcomings.

## Conclusion

A prognostic model based on fourteen TMB-related lncRNAs was established. Patients with high risk score were associated with worse prognosis in both the training set and all the validation sets. Besides, our model had the best predictive efficiency compared to other clinical factors and published models. We also discussed the potential mechanism of our model which provided new thought for precision treatment and biomarkers searching in pancreatic cancer.

## Electronic supplementary material

Below is the link to the electronic supplementary material.

Supplementary Material 1. Supplementary Figure 1. Prognosis analysis of the 14-lncRNAs prognostic model in the PDAC patients in the PACA-AU cohort. 

Supplementary Material 2. Supplementary Table 1. Differentially expressed lncRNAs between high-TMB and low-TMB groups in the TCGA-PAAD cohort.

## Data Availability

The datasets generated and/or analysed during the current study are available in the TCGA and the ICGC repository, https://portal.gdc.cancer.gov; https://dcc.icgc.org/.
