# Supplementary figures and images for: Tumor mutation burden-related long non-coding RNAs is predictor for prognosis and immune response in pancreatic cancer
Source: BMC Gastroenterol. 2022 Nov 29;22:495. doi: 10.1186/s12876-022-02535-z (PMC9710014; doi:10.1186/s12876-022-02535-z)

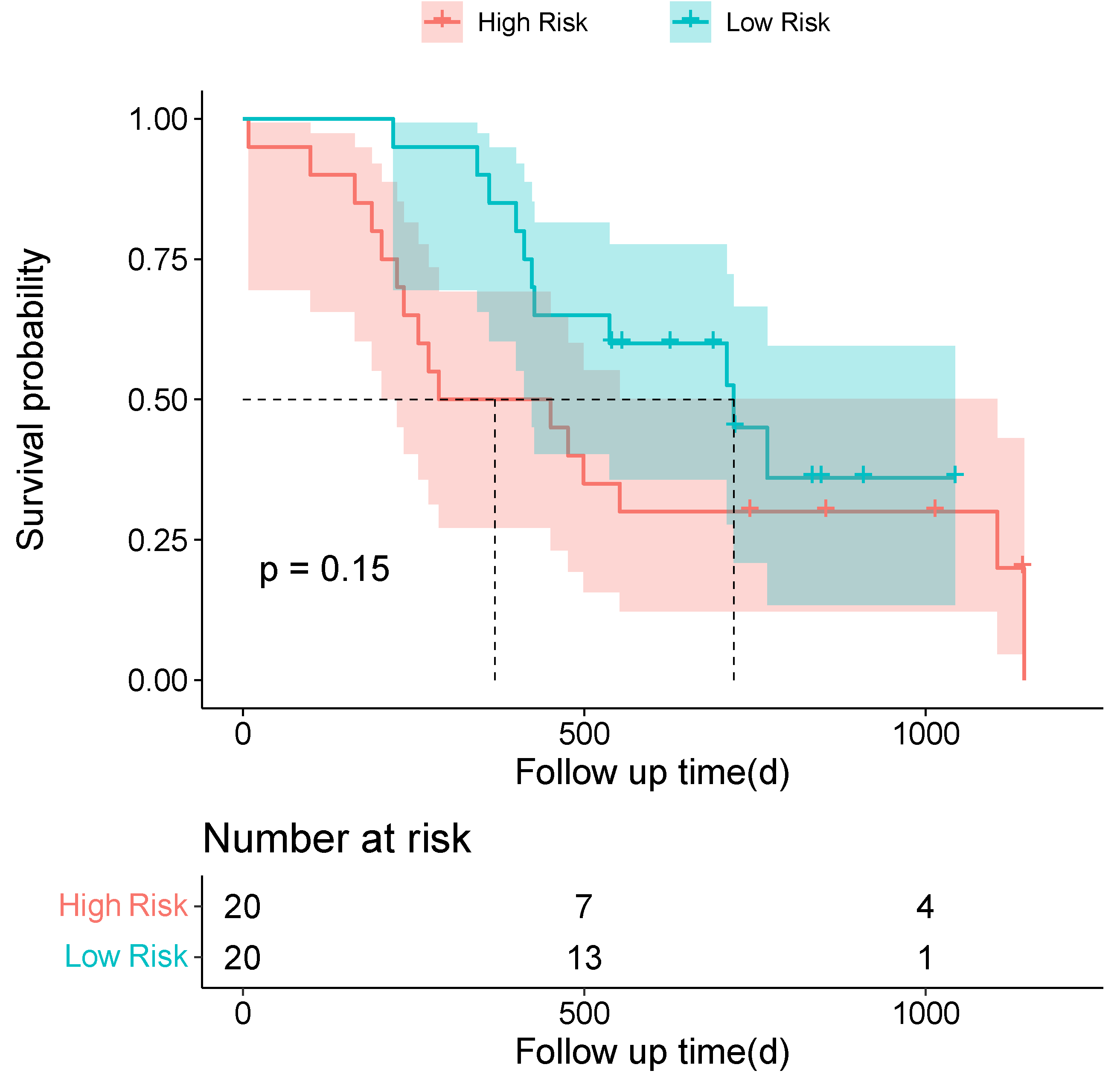

Supplement: Supplementary file 1 — Supplementary Material 1. Supplementary Figure 1. Prognosis analysis of the 14-lncRNAs prognostic model in the PDAC patients in the PACA-AU cohort. [file 12876_2022_2535_MOESM1_ESM.png]
