# Supplementary material for: Tumor mutation burden-related long non-coding RNAs is predictor for prognosis and immune response in pancreatic cancer
Source: BMC Gastroenterol. 2022 Nov 29;22:495. doi: 10.1186/s12876-022-02535-z (PMC9710014; doi:10.1186/s12876-022-02535-z)
Supplement: Supplementary file 2 — Supplementary Material 2. Supplementary Table 1. Differentially expressed lncRNAs between high-TMB and low-TMB groups in the TCGA-PAAD cohort. [file 12876_2022_2535_MOESM2_ESM.pdf]

upplementary Table 1. Differentially expressed lncRNAs between high-TMB and low-TMB groups in the TCGA-PAAD coho

ENSEMBL	symbol	group	logFC	PValue	FDR
ENSG00000245164	LINC00861	long_non_coding	-1.901033915	2.25E-14	3.34E-10
ENSG00000205056	LINC02397	long_non_coding	-2.360281066	2.55E-12	1.02E-08
ENSG00000255354	AC022239.2	long_non_coding	-2.498571018	3.32E-12	1.22E-08
ENSG00000235532	LINC00402	long_non_coding	-2.160889964	7.87E-12	1.74E-08
ENSG00000259834	AL365361.1	long_non_coding	-1.531862476	1.63E-11	2.85E-08
ENSG00000247982	LINC00926	long_non_coding	-1.760842501	2.18E-11	3.21E-08
ENSG00000228863	AL121985.1	long_non_coding	-1.249865019	8.70E-11	8.53E-08
ENSG00000237499	AL357060.2	long_non_coding	-0.867260157	1.01E-10	9.44E-08
ENSG00000233806	LINC01237	long_non_coding	-1.142108566	3.08E-10	2.09E-07
ENSG00000253522	MIR3142HG	long_non_coding	-1.350751819	5.76E-10	3.30E-07
ENSG00000248050	AC079061.1	long_non_coding	-1.819489346	7.13E-10	3.93E-07
ENSG00000228536	AL513283.1	long_non_coding	-2.300197726	1.04E-09	5.35E-07
ENSG00000198788	MUC2	long_non_coding	2.631660549	1.15E-09	5.45E-07
ENSG00000258545	RHOXF1-AS1	long_non_coding	1.901702961	1.58E-09	6.72E-07
ENSG00000238121	LINC00426	long_non_coding	-1.111422647	1.63E-09	6.77E-07
ENSG00000196668	LINC00173	long_non_coding	-1.39252254	1.81E-09	7.18E-07
ENSG00000255240	AP001636.3	long_non_coding	-1.101760012	1.98E-09	7.56E-07
ENSG00000258793	AL355102.4	long_non_coding	-1.523788406	2.74E-09	1.01E-06
ENSG00000187621	TCL6	long_non_coding	-1.656294388	4.00E-09	1.30E-06
ENSG00000227508	LINC01624	long_non_coding	-1.659242828	5.41E-09	1.66E-06
ENSG00000257151	PWAR6	long_non_coding	-0.805550731	6.36E-09	1.91E-06
ENSG00000226777	FAM30A	long_non_coding	-1.842980189	8.84E-09	2.42E-06
ENSG00000232470	AL136368.1	long_non_coding	-0.92451159	1.28E-08	3.34E-06
ENSG00000197291	RAMP2-AS1	long_non_coding	-0.825931743	1.42E-08	3.63E-06
ENSG00000226252	AL135960.1	long_non_coding	-0.93491426	1.69E-08	4.18E-06
ENSG00000237940	LINC01238	long_non_coding	-1.115923424	1.81E-08	4.37E-06
ENSG00000226237	GAS1RR	long_non_coding	-0.883938294	2.36E-08	5.37E-06
ENSG00000223403	MEG9	long_non_coding	-1.043897594	2.59E-08	5.80E-06
ENSG00000258810	AL133371.2	long_non_coding	-1.18836507	2.65E-08	5.86E-06
ENSG00000231483	AL365356.4	long_non_coding	1.043885153	2.78E-08	6.01E-06
ENSG00000260719	AC009133.3	long_non_coding	-1.378594043	2.83E-08	6.07E-06
ENSG00000224078	SNHG14	long_non_coding	-0.656050134	3.15E-08	6.63E-06
ENSG00000233665	AC060234.2	long_non_coding	-1.280090506	3.23E-08	6.71E-06
ENSG00000230747	AC021188.1	long_non_coding	-0.880269083	3.35E-08	6.92E-06
ENSG00000228956	SATB1-AS1	long_non_coding	-0.937858741	4.15E-08	8.26E-06
ENSG00000260997	AC004847.1	long_non_coding	-1.020907207	5.09E-08	9.36E-06
ENSG00000224614	TNK2-AS1	long_non_coding	-0.911302923	5.91E-08	1.05E-05
ENSG00000255760	LINC02422	long_non_coding	-1.816874511	6.56E-08	1.13E-05
ENSG00000235529	AGAP1-IT1	long_non_coding	1.226621657	7.09E-08	1.19E-05
ENSG00000225342	AC079630.1	long_non_coding	-0.712189512	7.23E-08	1.21E-05
ENSG00000255518	AC022239.3	long_non_coding	-2.307066399	7.36E-08	1.22E-05
ENSG00000253307	AC011676.1	long_non_coding	1.105048514	9.86E-08	1.52E-05
ENSG00000245954	LINC02273	long_non_coding	-1.363651716	9.92E-08	1.52E-05
ENSG00000232613	AC007386.2	long_non_coding	-1.301430263	1.00E-07	1.53E-05
ENSG00000261416	AC012645.3	long_non_coding	-1.089800457	1.16E-07	1.73E-05
ENSG00000224137	LINC01857	long_non_coding	-1.30221727	1.46E-07	2.09E-05
ENSG00000245904	AC025164.2	long_non_coding	-0.943219341	1.67E-07	2.27E-05
ENSG00000231566	AC234772.2	long_non_coding	0.815164419	1.75E-07	2.31E-05
ENSG00000233355	CHRM3-AS2	long_non_coding	-1.203190324	1.75E-07	2.31E-05
ENSG00000225670	CADM3-AS1	long_non_coding	-1.359492851	1.78E-07	2.33E-05
ENSG00000250986	AC141928.1	long_non_coding	-0.953638074	2.22E-07	2.75E-05
ENSG00000227155	AL161725.1	long_non_coding	-0.879360109	2.64E-07	3.16E-05
ENSG00000261553	AL137782.1	long_non_coding	0.768594637	2.76E-07	3.25E-05
ENSG00000257242	LINC01619	long_non_coding	-0.710961024	3.72E-07	3.95E-05
ENSG00000256982	AC135782.1	long_non_coding	1.205079281	4.52E-07	4.64E-05
ENSG00000240521	AC092979.1	long_non_coding	-2.64057916	4.97E-07	4.95E-05
ENSG00000261644	AC007728.2	long_non_coding	-0.873185273	5.23E-07	5.13E-05
ENSG00000259436	AC010247.2	long_non_coding	-1.026907767	5.34E-07	5.19E-05
ENSG00000247774	PCED1B-AS1	long_non_coding	-0.850564832	5.49E-07	5.31E-05

ENSG00000242258	LINC00996	long_non_coding	-0.932894155	5.64E-07	5.41E-05
ENSG00000225756	DBH-AS1	long_non_coding	-0.841947163	5.73E-07	5.48E-05
ENSG00000236901	MIR600HG	long_non_coding	-0.73316512	7.04E-07	6.34E-05
ENSG00000225948	AL158210.1	long_non_coding	-1.671047624	7.70E-07	6.78E-05
ENSG00000255020	AF131216.3	long_non_coding	-2.154523525	7.76E-07	6.80E-05
ENSG00000235304	LINC01281	long_non_coding	-1.286617834	9.46E-07	7.87E-05
ENSG00000236283	AC019197.1	long_non_coding	-1.085251954	9.73E-07	8.02E-05
ENSG00000261123	AC009065.5	long_non_coding	0.817776218	9.77E-07	8.03E-05
ENSG00000261069	AC124312.3	long_non_coding	-0.862566974	1.19E-06	9.38E-05
ENSG00000213373	LINC00671	long_non_coding	-1.620657941	1.20E-06	9.42E-05
ENSG00000257221	AC007569.1	long_non_coding	-1.019709401	1.21E-06	9.54E-05
ENSG00000259459	AC087525.1	long_non_coding	-1.272467881	1.22E-06	9.55E-05
ENSG00000231858	AC067945.3	long_non_coding	-0.869108373	1.29E-06	9.93E-05
ENSG00000259933	AC009065.2	long_non_coding	0.812046835	1.38E-06	0.000105303
ENSG00000231574	LINC02015	long_non_coding	-0.825277506	1.58E-06	0.000115762
ENSG00000234663	LINC01934	long_non_coding	-0.933779178	1.67E-06	0.000120159
ENSG00000231621	AC013264.1	long_non_coding	-1.339971872	1.86E-06	0.000129941
ENSG00000254602	AP000662.2	long_non_coding	-0.651117567	1.86E-06	0.000129941
ENSG00000216863	LY86-AS1	long_non_coding	-1.346608094	1.88E-06	0.00013102
ENSG00000232504	ST3GAL5-AS1	long_non_coding	-0.672712831	2.04E-06	0.000139428
ENSG00000240535	AC034238.1	long_non_coding	-1.065239626	2.38E-06	0.000156715
ENSG00000240143	AL023653.1	long_non_coding	-0.973861543	2.55E-06	0.000164395
ENSG00000214546	AC087491.1	long_non_coding	1.146731175	2.61E-06	0.000166346
ENSG00000256234	AC022509.2	long_non_coding	-0.606896026	2.77E-06	0.000175302
ENSG00000231226	TRIM31-AS1	long_non_coding	0.887743199	2.91E-06	0.000180963
ENSG00000257647	AC124312.2	long_non_coding	-0.802219169	3.06E-06	0.000187291
ENSG00000258053	AC025575.2	long_non_coding	1.353162711	3.26E-06	0.000197791
ENSG00000233559	LINC00513	long_non_coding	0.939777241	3.57E-06	0.000210325
ENSG00000259347	AC087482.1	long_non_coding	-1.485552049	4.27E-06	0.000243278
ENSG00000238078	LINC01352	long_non_coding	-0.717380616	4.47E-06	0.000252027
ENSG00000235621	LINC00494	long_non_coding	-1.327953221	4.59E-06	0.000256631
ENSG00000188242	PP7080	long_non_coding	0.732250506	4.67E-06	0.00026024
ENSG00000237914	SIRPG-AS1	long_non_coding	-1.200363757	4.81E-06	0.00026626
ENSG00000253364	AL928742.1	long_non_coding	-1.583829473	4.86E-06	0.000267765
ENSG00000245694	CRNDE	long_non_coding	0.697761843	5.34E-06	0.000287172
ENSG00000228639	LINC02095	long_non_coding	-1.528035869	5.52E-06	0.000294928
ENSG00000240219	AL512306.2	long_non_coding	-0.888517	5.66E-06	0.000300002
ENSG00000236081	ELFN1-AS1	long_non_coding	1.235415701	5.76E-06	0.0003029
ENSG00000226571	AL158850.1	long_non_coding	-1.549164131	5.93E-06	0.000309757
ENSG00000214548	MEG3	long_non_coding	-0.714674895	6.72E-06	0.000341357
ENSG00000223511	AL683807.1	long_non_coding	-0.905966129	6.80E-06	0.000344264
ENSG00000253508	AC004080.2	long_non_coding	1.501752959	7.00E-06	0.000350568
ENSG00000227145	IL21-AS1	long_non_coding	-1.544779126	7.04E-06	0.000351392
ENSG00000236935	AP003774.4	long_non_coding	-0.995056945	7.05E-06	0.000351392
ENSG00000237513	AC007384.1	long_non_coding	-0.992154472	7.24E-06	0.000358913
ENSG00000260101	AC008074.3	long_non_coding	-0.588840382	7.53E-06	0.000367722
ENSG00000251504	LINC01099	long_non_coding	-1.501556368	9.06E-06	0.000422459
ENSG00000250850	AL161781.2	long_non_coding	-2.18398328	9.20E-06	0.00042654
ENSG00000229261	AL596223.1	long_non_coding	0.808188052	1.01E-05	0.000458709
ENSG00000261218	AC099524.1	long_non_coding	-1.112888561	1.03E-05	0.000464154
ENSG00000225938	AL109741.1	long_non_coding	-0.657962964	1.05E-05	0.000472663
ENSG00000225541	AC002480.1	long_non_coding	-1.008970834	1.08E-05	0.000482652
ENSG00000258919	AL049836.1	long_non_coding	0.979385695	1.12E-05	0.000495312
ENSG00000256582	LINC02390	long_non_coding	-1.456873754	1.17E-05	0.000515089
ENSG00000237248	LINC00987	long_non_coding	-0.749427341	1.18E-05	0.00051673
ENSG00000240990	HOXA11-AS	long_non_coding	1.314046519	1.19E-05	0.000520814
ENSG00000234883	MIR155HG	long_non_coding	-0.693064994	1.20E-05	0.000521063
ENSG00000251191	LINC00589	long_non_coding	0.848096822	1.21E-05	0.000524983
ENSG00000230155	FO393401.1	long_non_coding	-0.731911766	1.29E-05	0.000551373
ENSG00000246084	LINC02325	long_non_coding	-0.97313047	1.30E-05	0.000553628
ENSG00000234389	AC007278.1	long_non_coding	-0.978123618	1.32E-05	0.00056037
ENSG00000259070	LINC00639	long_non_coding	-0.695768827	1.33E-05	0.00056179

ENSG00000254281	AP003354.2	long_non_coding	-0.600577296	1.34E-05	0.000564276
ENSG00000254528	AP000757.1	long_non_coding	-0.829979173	1.37E-05	0.000577653
ENSG00000176984	AP000679.1	long_non_coding	0.842893517	1.44E-05	0.000598462
ENSG00000224565	LINC01754	long_non_coding	-1.652712915	1.44E-05	0.000598462
ENSG00000253678	AC104964.1	long_non_coding	0.952738437	1.48E-05	0.000606761
ENSG00000233005	AC018742.1	long_non_coding	-1.061224805	1.52E-05	0.0006192
ENSG00000229393	AL139246.4	long_non_coding	-0.995878965	1.52E-05	0.000620602
ENSG00000259771	AC092756.1	long_non_coding	0.777499399	1.55E-05	0.000628046
ENSG00000222033	LINC01124	long_non_coding	0.823585247	1.64E-05	0.000661787
ENSG00000230138	AC119428.2	long_non_coding	-1.242216272	1.68E-05	0.000671317
ENSG00000238099	LINC01625	long_non_coding	-1.402202546	1.69E-05	0.000674383
ENSG00000259495	AC016705.2	long_non_coding	-0.6782548	1.79E-05	0.000706875
ENSG00000225963	AC009950.1	long_non_coding	-0.629529586	1.82E-05	0.000713923
ENSG00000240710	AL512306.3	long_non_coding	-0.94797891	1.87E-05	0.00072677
ENSG00000237943	PRKCQ-AS1	long_non_coding	-0.727506313	1.91E-05	0.000738252
ENSG00000241158	ADAMTS9-AS1	long_non_coding	-0.878136331	1.92E-05	0.000741268
ENSG00000214894	LINC00243	long_non_coding	-0.729717233	2.06E-05	0.000787421
ENSG00000231105	AL031728.1	long_non_coding	-0.706784012	2.12E-05	0.000799354
ENSG00000256969	AC007207.2	long_non_coding	-1.828445141	2.14E-05	0.000802598
ENSG00000249601	LINC01187	long_non_coding	-1.275532486	2.27E-05	0.000841203
ENSG00000234572	LINC01800	long_non_coding	-1.443619814	2.38E-05	0.000868435
ENSG00000228401	HSPC324	long_non_coding	-0.68531896	2.44E-05	0.000886272
ENSG00000261448	AC109446.3	long_non_coding	-0.973690887	2.47E-05	0.000890684
ENSG00000233308	OSTN-AS1	long_non_coding	-1.191871369	2.49E-05	0.000897751
ENSG00000230698	AC105935.2	long_non_coding	1.07450262	2.55E-05	0.000914267
ENSG00000249476	AC008467.1	long_non_coding	-0.647426614	2.58E-05	0.000922998
ENSG00000226871	AC135178.1	long_non_coding	-1.076345398	2.68E-05	0.000946945
ENSG00000122548	KIAA0087	long_non_coding	-0.806142409	2.74E-05	0.000964217
ENSG00000233093	LINC00892	long_non_coding	-0.915812291	2.76E-05	0.000968017
ENSG00000214851	LINC00612	long_non_coding	-0.725705415	3.02E-05	0.00103449
ENSG00000253552	HOXA-AS2	long_non_coding	0.588810248	3.02E-05	0.00103449
ENSG00000258913	C14orf144	long_non_coding	-0.797650308	3.11E-05	0.001053296
ENSG00000232803	SLCO4A1-AS1	long_non_coding	0.982356749	3.19E-05	0.001072615
ENSG00000253434	LINC02237	long_non_coding	-1.417287653	3.59E-05	0.001177585
ENSG00000250889	LINC01336	long_non_coding	-0.595979948	3.63E-05	0.001186657
ENSG00000181908	AP003774.1	long_non_coding	-0.698878751	3.65E-05	0.001190817
ENSG00000251513	LIX1-AS1	long_non_coding	-1.210146442	3.74E-05	0.00121036
ENSG00000229425	AJ009632.2	long_non_coding	-0.758313344	3.77E-05	0.001218301
ENSG00000260803	Z84723.1	long_non_coding	-1.025212026	3.84E-05	0.001231955
ENSG00000261604	AC114947.2	long_non_coding	0.624578681	3.83E-05	0.001231955
ENSG00000226808	LINC00840	long_non_coding	-0.694340439	3.86E-05	0.001234915
ENSG00000245812	LINC02202	long_non_coding	-0.62857246	4.23E-05	0.001318503
ENSG00000248243	LINC02014	long_non_coding	0.627975849	4.27E-05	0.001328857
ENSG00000181800	CELF2-AS1	long_non_coding	-0.975419161	4.40E-05	0.001362719
ENSG00000231682	LINC01891	long_non_coding	-1.445792481	4.55E-05	0.001401357
ENSG00000231768	LINC01354	long_non_coding	-0.680406889	4.74E-05	0.00143831
ENSG00000256427	AC010175.1	long_non_coding	-0.81771377	4.90E-05	0.001473338
ENSG00000184608	FAM167A-AS1	long_non_coding	-1.664378725	4.92E-05	0.001474593
ENSG00000251129	LINC02506	long_non_coding	-2.325618327	4.92E-05	0.001474593
ENSG00000248015	AC005329.1	long_non_coding	-0.823536951	5.10E-05	0.001516022
ENSG00000241657	TRBV11-2	long_non_coding	-0.933765164	5.17E-05	0.001531405
ENSG00000231290	APCDD1L-AS1	long_non_coding	1.200214136	5.26E-05	0.001550952
ENSG00000254887	AC010247.1	long_non_coding	-0.826758931	5.26E-05	0.001550952
ENSG00000228427	AL590764.1	long_non_coding	-0.726220458	5.33E-05	0.001563902
ENSG00000253404	AC034243.1	long_non_coding	-1.007831978	5.45E-05	0.001589979
ENSG00000248996	AC145098.1	long_non_coding	-0.614113681	5.47E-05	0.001594762
ENSG00000179253	AL162457.1	long_non_coding	-1.233017744	5.75E-05	0.001651886
ENSG00000230266	XXYLT1-AS2	long_non_coding	0.868369003	5.76E-05	0.001653327
ENSG00000217455	AC073316.1	long_non_coding	1.182443624	5.85E-05	0.001669149
ENSG00000255306	AC004923.4	long_non_coding	0.628227876	5.96E-05	0.00169203
ENSG00000230530	LIMD1-AS1	long_non_coding	-0.61181168	5.99E-05	0.001699222
ENSG00000229380	AC147651.1	long_non_coding	-1.148223824	6.14E-05	0.001732903

ENSG00000234184	LINC01781	long_non_coding	-1.276279605	6.16E-05	0.001736708
ENSG00000260542	AL499627.1	long_non_coding	1.772007886	6.33E-05	0.001771046
ENSG00000233577	AL022332.1	long_non_coding	-1.181866473	6.37E-05	0.001780215
ENSG00000226308	AL122058.1	long_non_coding	1.330696099	6.50E-05	0.00181034
ENSG00000226239	AL031658.1	long_non_coding	-0.638557548	6.84E-05	0.001884392
ENSG00000232884	AF127936.2	long_non_coding	-1.015056526	6.83E-05	0.001884392
ENSG00000259905	PWRN1	long_non_coding	-0.983222112	6.90E-05	0.001896618
ENSG00000235419	AC010149.1	long_non_coding	-0.824154838	6.90E-05	0.00189687
ENSG00000223461	AC004471.1	long_non_coding	0.710724506	7.07E-05	0.001929496
ENSG00000227959	AL451042.2	long_non_coding	0.595429168	7.09E-05	0.001930849
ENSG00000235111	Z97192.3	long_non_coding	-0.786911588	7.13E-05	0.001937078
ENSG00000251002	AC244502.1	long_non_coding	-0.652994704	7.16E-05	0.001946319
ENSG00000253686	LINC01484	long_non_coding	-0.651536968	7.33E-05	0.001981145
ENSG00000255474	AC005833.2	long_non_coding	1.024174107	8.02E-05	0.002128675
ENSG00000228689	AL355997.1	long_non_coding	-1.000049091	8.03E-05	0.002130267
ENSG00000226197	AL583785.1	long_non_coding	-1.05764725	8.09E-05	0.002142188
ENSG00000239636	AC004865.2	long_non_coding	-0.609493571	8.21E-05	0.002165623
ENSG00000241490	AC093010.2	long_non_coding	-1.104369451	8.38E-05	0.00220099
ENSG00000253641	LINCR-0001	long_non_coding	1.009424762	8.63E-05	0.002242136
ENSG00000258404	LINC02320	long_non_coding	0.897815007	8.63E-05	0.002242136
ENSG00000228590	AC007381.1	long_non_coding	-1.828340297	8.74E-05	0.002261197
ENSG00000229424	AC007349.1	long_non_coding	-1.353091721	9.02E-05	0.002310419
ENSG00000261617	LINC02177	long_non_coding	-0.789864036	9.91E-05	0.002476776
ENSG00000241475	AL160408.4	long_non_coding	1.634962034	0.000101263	0.002516911
ENSG00000259810	AC002519.1	long_non_coding	-0.727809784	0.000103123	0.002550206
ENSG00000259471	LINC01169	long_non_coding	1.28637876	0.000103311	0.002550564
ENSG00000205231	TTLL10-AS1	long_non_coding	-0.836986161	0.000103487	0.002553493
ENSG00000182366	FAM87A	long_non_coding	-0.813528213	0.000107815	0.002639607
ENSG00000260902	LINC02011	long_non_coding	1.09890542	0.000111513	0.002712078
ENSG00000241666	AL031733.2	long_non_coding	-0.981571506	0.000116994	0.002817431
ENSG00000248441	LINC01197	long_non_coding	-0.617880405	0.000118399	0.002846611
ENSG00000257275	AL139020.1	long_non_coding	-2.118265418	0.000118656	0.002849688
ENSG00000235151	AC131097.4	long_non_coding	-0.634416607	0.000119556	0.002863513
ENSG00000259519	AC051619.4	long_non_coding	0.832202699	0.000123948	0.002942224
ENSG00000236780	LINC01829	long_non_coding	-1.160991305	0.00012991	0.003049447
ENSG00000249667	LINC01259	long_non_coding	-1.567039962	0.000134945	0.003128025
ENSG00000261770	AC006504.1	long_non_coding	-0.596671496	0.000143092	0.003276046
ENSG00000260838	AC022893.2	long_non_coding	-0.970414161	0.000144499	0.003301009
ENSG00000224743	TEX26-AS1	long_non_coding	-0.811143051	0.000145489	0.003315437
ENSG00000226812	AL117382.1	long_non_coding	0.795963095	0.000147155	0.003343051
ENSG00000254988	AP002498.1	long_non_coding	1.056799069	0.000150246	0.003395785
ENSG00000259004	LINC02285	long_non_coding	-0.643133904	0.000163621	0.0036366
ENSG00000248309	MEF2C-AS1	long_non_coding	-0.609765233	0.00016909	0.003720654
ENSG00000225194	LINC00092	long_non_coding	-0.591689926	0.000170676	0.003746202
ENSG00000235576	LINC01871	long_non_coding	-0.619054822	0.000171558	0.003759964
ENSG00000249695	AC026369.1	long_non_coding	-0.656018274	0.000175296	0.003809706
ENSG00000231106	LINC01436	long_non_coding	0.997335401	0.000185316	0.003974607
ENSG00000258926	AL355916.2	long_non_coding	-0.604515171	0.000187106	0.004005219
ENSG00000255146	AP004247.2	long_non_coding	-1.113346564	0.000188288	0.004020765
ENSG00000259881	AC092384.2	long_non_coding	-0.76392048	0.000192734	0.004093905
ENSG00000223669	AL357033.2	long_non_coding	1.097090877	0.000193747	0.004103567
ENSG00000237990	CNTN4-AS1	long_non_coding	-1.174472941	0.000195793	0.004136978
ENSG00000240922	LSAMP-AS1	long_non_coding	1.023202125	0.000197586	0.004166879
ENSG00000231605	LINC01363	long_non_coding	2.182851201	0.000209263	0.004334406
ENSG00000225746	MEG8	long_non_coding	-0.601879218	0.00021114	0.004358115
ENSG00000231769	AL035701.1	long_non_coding	-0.590345594	0.000211249	0.004358115
ENSG00000224387	AL139246.1	long_non_coding	-0.883420374	0.000213736	0.004389949
ENSG00000259153	AC004816.1	long_non_coding	0.599747566	0.000215804	0.004411872
ENSG00000228857	AC104653.1	long_non_coding	-0.5947625	0.000224015	0.004539786
ENSG00000122043	LINC00544	long_non_coding	-0.992877254	0.000226721	0.004584077
ENSG00000237181	AC147651.3	long_non_coding	-0.593571233	0.000242225	0.00482242
ENSG00000259238	AC092755.2	long_non_coding	0.708096907	0.000249208	0.004915178

ENSG00000230970	HHATL-AS1	long_non_coding	-1.166141805	0.000256717	0.005013653
ENSG00000257345	LINC02413	long_non_coding	-1.064890935	0.00026395	0.005120899
ENSG00000261471	AC092145.1	long_non_coding	-0.781340145	0.000273097	0.005247228
ENSG00000229922	LINC02528	long_non_coding	-1.482433661	0.000275132	0.005275224
ENSG00000259954	IL21R-AS1	long_non_coding	-0.718322086	0.000279798	0.005345313
ENSG00000255733	IFNG-AS1	long_non_coding	-1.032632366	0.000281286	0.005355958
ENSG00000254369	HOXA-AS3	long_non_coding	0.847424946	0.000287564	0.005454324
ENSG00000230489	VAV3-AS1	long_non_coding	-1.519576794	0.000297333	0.005605879
ENSG00000261266	AC008870.3	long_non_coding	0.760148788	0.000297755	0.005611421
ENSG00000229646	AL109914.1	long_non_coding	-1.29211768	0.000301486	0.0056648
ENSG00000249395	CASC9	long_non_coding	1.404428834	0.000318102	0.005906585
ENSG00000246430	LINC00968	long_non_coding	-0.724476095	0.000320574	0.005939976
ENSG00000257551	HLX-AS1	long_non_coding	-0.657845651	0.00032633	0.006018416
ENSG00000205562	AL049775.1	long_non_coding	-0.609136734	0.0003324	0.006100278
ENSG00000230316	FEZF1-AS1	long_non_coding	1.10178758	0.000356808	0.006430422
ENSG00000233461	AL445524.1	long_non_coding	0.590789253	0.000357541	0.006438365
ENSG00000246528	AC079089.1	long_non_coding	-0.727959211	0.000367286	0.006562942
ENSG00000204110	LINC02520	long_non_coding	-1.307390017	0.000367518	0.006564432
ENSG00000233760	AC004947.1	long_non_coding	-0.700547337	0.000371575	0.006604821
ENSG00000255197	AC090559.1	long_non_coding	-0.587501472	0.000385691	0.006810452
ENSG00000258175	LINC02300	long_non_coding	1.693343781	0.000387095	0.006825685
ENSG00000228835	AC012123.1	long_non_coding	-1.352338277	0.000392747	0.006892283
ENSG00000253690	AC021678.2	long_non_coding	-1.086170683	0.000422687	0.007273049
ENSG00000225783	MIAT	long_non_coding	-0.663386589	0.000434042	0.007419245
ENSG00000232692	AP001596.1	long_non_coding	-0.655338603	0.000433871	0.007419245
ENSG00000261222	AC064805.1	long_non_coding	-0.719968381	0.000436872	0.007459402
ENSG00000225077	LINC00337	long_non_coding	0.630996611	0.000438577	0.007479381
ENSG00000236095	AL807757.2	long_non_coding	0.770836986	0.000441805	0.007525691
ENSG00000237978	KCNMB2-AS1	long_non_coding	1.204421147	0.000445739	0.007578079
ENSG00000228055	LINC00864	long_non_coding	-1.28187741	0.000450512	0.007632751
ENSG00000233791	LINC01136	long_non_coding	-0.753660071	0.000453417	0.007665277
ENSG00000224322	AC004009.1	long_non_coding	0.925838638	0.000457792	0.007722874
ENSG00000258331	LINC02461	long_non_coding	-0.911214569	0.00046107	0.007769864
ENSG00000187185	AC092118.1	long_non_coding	-0.751267865	0.000467581	0.007852755
ENSG00000224652	LINC00885	long_non_coding	-0.847298861	0.000468177	0.007859608
ENSG00000228962	HCG23	long_non_coding	-0.653978225	0.000488667	0.00811405
ENSG00000233776	LINC01251	long_non_coding	-0.980070059	0.000491291	0.008154537
ENSG00000254041	AC021546.1	long_non_coding	-0.761990502	0.00049736	0.00823977
ENSG00000250415	AC022113.2	long_non_coding	-1.155402306	0.000507422	0.008356267
ENSG00000251165	F11-AS1	long_non_coding	-0.877695274	0.000522214	0.008552269
ENSG00000231079	AC105402.3	long_non_coding	-0.95835933	0.000525789	0.008594582
ENSG00000226751	AF127936.1	long_non_coding	-0.691689993	0.000544986	0.008820139
ENSG00000235688	AC116614.1	long_non_coding	-0.916209082	0.000559039	0.008984952
ENSG00000261357	AC099518.2	long_non_coding	0.660080432	0.000565118	0.009056253
ENSG00000249684	AC106795.2	long_non_coding	-0.795641408	0.000568698	0.009100398
ENSG00000251301	LINC02384	long_non_coding	-0.754783099	0.000571232	0.009127717
ENSG00000230289	AL358781.2	long_non_coding	-0.615045107	0.000572137	0.009133125
ENSG00000258483	LINC02251	long_non_coding	-0.997026823	0.000572355	0.009133125
ENSG00000228262	LINC01320	long_non_coding	-1.12012483	0.000576955	0.009189203
ENSG00000236711	SMAD9-IT1	long_non_coding	-0.765658972	0.000585729	0.009307376
ENSG00000231943	PGM5P4-AS1	long_non_coding	-0.784168284	0.000597581	0.009432584
ENSG00000227131	AL139275.2	long_non_coding	-1.523340255	0.000616656	0.009650803
ENSG00000258876	TGFB3-AS1	long_non_coding	-0.835087411	0.000646056	0.009993929
ENSG00000244310	AC093904.3	long_non_coding	0.970742916	0.000651259	0.010046235
ENSG00000228643	AC079779.2	long_non_coding	-0.825721246	0.000670387	0.010277321
ENSG00000256706	AC005342.2	long_non_coding	-0.830573396	0.000677738	0.010360516
ENSG00000249926	AC117500.2	long_non_coding	-1.02930539	0.000687624	0.01048289
ENSG00000237352	LINC01358	long_non_coding	-0.707436711	0.000688733	0.010492818
ENSG00000241269	AC093620.1	long_non_coding	0.651944111	0.000699973	0.010593971
ENSG00000248115	AC023154.1	long_non_coding	-0.626900832	0.000702911	0.010634782
ENSG00000235237	Z82188.2	long_non_coding	-0.63670344	0.000709084	0.010718585
ENSG00000233110	AC093797.1	long_non_coding	-0.620153773	0.000721234	0.010848759

ENSG00000099869	IGF2-AS	long_non_coding	-0.826194144	0.000734796	0.01100318
ENSG00000256633	AP005019.1	long_non_coding	-0.604902136	0.000747951	0.011126662
ENSG00000254981	AC087623.1	long_non_coding	-0.693221917	0.000754293	0.011185853
ENSG00000253308	AC004080.1	long_non_coding	1.531826596	0.000754592	0.011186518
ENSG00000226702	MIR217HG	long_non_coding	-1.653611473	0.000757454	0.011225181
ENSG00000228044	AL160408.1	long_non_coding	1.016599133	0.000767132	0.011327521
ENSG00000231465	AL359636.2	long_non_coding	1.155312357	0.000768309	0.01134033
ENSG00000237928	NFIA-AS2	long_non_coding	-0.626723282	0.000772609	0.011373354
ENSG00000225350	FREM2-AS1	long_non_coding	-2.148669024	0.000776749	0.011422865
ENSG00000225472	AL136366.1	long_non_coding	-0.691223791	0.00078583	0.011521844
ENSG00000251138	AC090502.1	long_non_coding	0.976697188	0.000816492	0.011884522
ENSG00000258867	LINC01146	long_non_coding	-0.74431556	0.000844511	0.012138947
ENSG00000228549	BX284668.2	long_non_coding	-0.735675773	0.0008473	0.01216835
ENSG00000231405	AL008638.1	long_non_coding	-1.250671629	0.000863503	0.012324815
ENSG00000257495	KRT73-AS1	long_non_coding	-1.710347771	0.000864172	0.012330386
ENSG00000229151	AC233976.1	long_non_coding	-0.71826957	0.000869143	0.012361337
ENSG00000242147	AL365356.5	long_non_coding	0.691540071	0.00090966	0.012809565
ENSG00000250385	AC106772.2	long_non_coding	1.210268635	0.000920619	0.012943237
ENSG00000253405	EVX1-AS	long_non_coding	1.478492602	0.000929387	0.012996804
ENSG00000236532	LINC01695	long_non_coding	-0.794730876	0.000936421	0.013048962
ENSG00000255650	FAM222A-AS1	long_non_coding	0.624605738	0.000963096	0.013323887
ENSG00000243766	HOTTIP	long_non_coding	1.208110288	0.000973595	0.013402314
ENSG00000234211	AL451067.1	long_non_coding	-1.220768349	0.000977825	0.0134266
ENSG00000248991	AC097375.1	long_non_coding	-1.539666496	0.00098005	0.013440964
ENSG00000258413	AL158801.2	long_non_coding	0.661938894	0.000988379	0.013537811
ENSG00000235939	AL138760.1	long_non_coding	1.089802886	0.00099374	0.013587941
ENSG00000259235	AC066612.1	long_non_coding	-0.956446662	0.001029564	0.013933226
ENSG00000241525	AC141424.1	long_non_coding	-0.959452647	0.001043239	0.014075114
ENSG00000260592	AC130456.3	long_non_coding	0.614168908	0.001043159	0.014075114
ENSG00000180458	AC022148.1	long_non_coding	0.804945417	0.001052059	0.01416858
ENSG00000232759	AC002480.2	long_non_coding	-0.638461293	0.0011047	0.014631391
ENSG00000250413	AC005674.1	long_non_coding	-0.589783876	0.001154173	0.015154704
ENSG00000235142	LINC02532	long_non_coding	-0.899183297	0.001175078	0.01529265
ENSG00000259793	AC013726.1	long_non_coding	-0.775352377	0.001218575	0.015660091
ENSG00000250682	LINC00491	long_non_coding	2.162842679	0.001233003	0.015817865
ENSG00000258260	AC073896.5	long_non_coding	-0.998582802	0.001243574	0.015925686
ENSG00000250579	AC022424.1	long_non_coding	-0.820972174	0.001244762	0.015936271
ENSG00000236975	AL137793.1	long_non_coding	-0.68678741	0.001265615	0.016123711
ENSG00000250155	AC008957.1	long_non_coding	-0.652687353	0.001266881	0.016130515
ENSG00000256232	LINC02387	long_non_coding	0.723847182	0.001297875	0.01641619
ENSG00000222001	AC106876.1	long_non_coding	0.734114216	0.001306597	0.016507572
ENSG00000260372	AQP4-AS1	long_non_coding	-0.614892495	0.001308438	0.016526096
ENSG00000236671	PRKG1-AS1	long_non_coding	0.593386341	0.001322124	0.016675086
ENSG00000250969	AC097658.2	long_non_coding	-1.137348017	0.001333545	0.01677119
ENSG00000224875	AC083949.1	long_non_coding	-0.684763767	0.001335187	0.016776073
ENSG00000249201	CTD-3080P12.3	long_non_coding	-0.828821064	0.001335292	0.016776073
ENSG00000238279	BX470102.1	long_non_coding	0.717766296	0.001374201	0.017116605
ENSG00000241163	LINC00877	long_non_coding	-0.621741795	0.00137606	0.017130577
ENSG00000258476	LINC02207	long_non_coding	-0.642760865	0.0013921	0.017250829
ENSG00000204971	AP000812.1	long_non_coding	-0.762699813	0.001418741	0.017474044
ENSG00000249993	BFSP2-AS1	long_non_coding	-0.990152487	0.001429894	0.01759014
ENSG00000233423	AC005482.1	long_non_coding	-1.182397094	0.001443081	0.017724346
ENSG00000256862	AC005842.1	long_non_coding	-0.850723529	0.001449849	0.01778272
ENSG00000231023	LINC00326	long_non_coding	1.57912797	0.001478667	0.018025928
ENSG00000254192	AC011365.2	long_non_coding	-0.669691495	0.001540697	0.018541321
ENSG00000223783	LINC01983	long_non_coding	0.964668743	0.001563941	0.018754538
ENSG00000249650	AC106772.1	long_non_coding	0.912828563	0.001569096	0.018785725
ENSG00000255605	AP000820.1	long_non_coding	-0.936413415	0.001651054	0.019563603
ENSG00000260271	AL132996.1	long_non_coding	-1.120237645	0.001652909	0.019563603
ENSG00000183242	WT1-AS	long_non_coding	-0.587347771	0.001729768	0.020269301
ENSG00000171889	MIR31HG	long_non_coding	0.88142784	0.001786481	0.020784804
ENSG00000228403	AC035139.1	long_non_coding	-0.829015568	0.001831193	0.021198781

ENSG00000259293	LIPC-AS1	long_non_coding	-0.789197136	0.001840403	0.02127408
ENSG00000261839	AL358933.1	long_non_coding	-0.620186492	0.001848064	0.021332521
ENSG00000242795	AC007849.1	long_non_coding	0.885650807	0.001879433	0.021587298
ENSG00000258092	AC005841.1	long_non_coding	0.701800272	0.001886649	0.021650087
ENSG00000260769	AC007614.4	long_non_coding	-1.107198366	0.001913333	0.021832951
ENSG00000258572	AL133467.1	long_non_coding	-0.71480921	0.001946577	0.022102526
ENSG00000247095	MIR210HG	long_non_coding	0.658708074	0.001990601	0.022466445
ENSG00000225420	AC104134.1	long_non_coding	0.73486929	0.002002791	0.02256938
ENSG00000231324	AP000696.1	long_non_coding	0.768239742	0.002049464	0.022937146
ENSG00000258449	AC023510.1	long_non_coding	-0.876398866	0.002093161	0.023319834
ENSG00000229896	AL157373.2	long_non_coding	0.647562205	0.002140247	0.023671314
ENSG00000250696	AC111000.4	long_non_coding	-0.960594407	0.002148052	0.023721592
ENSG00000239219	AC008040.1	long_non_coding	-0.681530351	0.002187628	0.024044283
ENSG00000233850	AC103563.7	long_non_coding	-1.22611943	0.002190051	0.02405294
ENSG00000250891	LINC02208	long_non_coding	-1.128734133	0.002199623	0.024125588
ENSG00000227920	AL353597.1	long_non_coding	-1.472375538	0.002248113	0.024495492
ENSG00000218018	AL109955.1	long_non_coding	-0.593245813	0.002254491	0.024540745
ENSG00000260989	AL133297.2	long_non_coding	0.881068315	0.002282302	0.024764039
ENSG00000230960	AL109910.1	long_non_coding	-1.948571887	0.002322038	0.025071871
ENSG00000230836	LINC01293	long_non_coding	0.815388111	0.002342694	0.025251641
ENSG00000224879	AC011754.1	long_non_coding	-1.366718075	0.00237946	0.025541292
ENSG00000231013	AC013275.1	long_non_coding	-0.841049964	0.002408618	0.025754582
ENSG00000253618	GRPEL2-AS1	long_non_coding	-0.59080479	0.002421166	0.025869947
ENSG00000259439	LINC01833	long_non_coding	0.99485385	0.002524637	0.026684702
ENSG00000253852	AC011383.1	long_non_coding	-0.60613961	0.002548417	0.026906385
ENSG00000226806	AC011893.1	long_non_coding	-0.684212603	0.002579463	0.027098246
ENSG00000256725	AC095350.2	long_non_coding	-1.133951349	0.002599287	0.027232333
ENSG00000250564	AC109454.3	long_non_coding	1.319703104	0.002630018	0.027443499
ENSG00000224220	AC104699.1	long_non_coding	-0.744498742	0.002651704	0.027637107
ENSG00000230676	AL353803.2	long_non_coding	-0.822436698	0.002654815	0.027656462
ENSG00000253406	AC012613.2	long_non_coding	0.671386926	0.002678971	0.027822015
ENSG00000177335	C8orf31	long_non_coding	0.771805178	0.002709675	0.028003149
ENSG00000230696	AC011753.2	long_non_coding	1.189851532	0.00276138	0.028416262
ENSG00000235934	AC007405.2	long_non_coding	0.771619904	0.002761326	0.028416262
ENSG00000230258	AC005208.1	long_non_coding	-0.888574622	0.002801313	0.028656081
ENSG00000250056	LINC01018	long_non_coding	-0.673934858	0.002804982	0.028665786
ENSG00000249988	AC092546.1	long_non_coding	-1.050750712	0.002866431	0.029118402
ENSG00000259398	AC090970.1	long_non_coding	0.778931	0.002937692	0.02963733
ENSG00000230126	FGF12-AS2	long_non_coding	-0.84478178	0.002954361	0.029724168
ENSG00000229876	CASC20	long_non_coding	1.572141182	0.002963872	0.029792683
ENSG00000232715	LINC01022	long_non_coding	1.098361595	0.002993002	0.030040788
ENSG00000230506	AL354824.1	long_non_coding	-0.690621341	0.003005608	0.030132872
ENSG00000249534	LINC01258	long_non_coding	0.799969364	0.003059424	0.030523673
ENSG00000228625	AL451047.1	long_non_coding	0.876353062	0.003104474	0.030802755
ENSG00000186235	AC016757.1	long_non_coding	-0.592312305	0.003138964	0.03106434
ENSG00000254100	AC069120.3	long_non_coding	-0.805681749	0.003145203	0.031105147
ENSG00000244620	AC246787.2	long_non_coding	-1.182475528	0.00315214	0.031159781
ENSG00000238178	AC078993.1	long_non_coding	-0.791327754	0.003265997	0.032048326
ENSG00000226828	AL591885.1	long_non_coding	-0.909124339	0.003272732	0.032107268
ENSG00000213468	FIRRE	long_non_coding	0.690317891	0.003280088	0.032151856
ENSG00000225126	AL365194.1	long_non_coding	-0.871547847	0.00328696	0.032189589
ENSG00000256124	LINC01152	long_non_coding	-0.632078852	0.003371713	0.032821026
ENSG00000230121	AL445070.1	long_non_coding	-1.090107485	0.003374085	0.032824227
ENSG00000206129	AC006305.1	long_non_coding	1.078665007	0.003386004	0.032930671
ENSG00000248112	AC108174.1	long_non_coding	1.116243624	0.003387958	0.032937398
ENSG00000227744	LINC01940	long_non_coding	1.099128258	0.003454031	0.033445309
ENSG00000226423	AC093642.1	long_non_coding	-1.412745144	0.003457996	0.03346345
ENSG00000238133	MAP3K20-AS1	long_non_coding	0.933150248	0.003546055	0.034024548
ENSG00000214039	LINC02418	long_non_coding	1.213417016	0.00358998	0.034321181
ENSG00000260710	AC120498.4	long_non_coding	0.677097644	0.003598503	0.034353429
ENSG00000234156	AL162254.1	long_non_coding	1.017356574	0.003631139	0.034577723
ENSG00000228382	ITPKB-IT1	long_non_coding	-0.908823137	0.003659753	0.034795617

ENSG00000233515	LINC01518	long_non_coding	1.626610081	0.00366033	0.034795617
ENSG00000230061	TRPM2-AS	long_non_coding	0.853254829	0.003694523	0.035060223
ENSG00000250387	LINC02197	long_non_coding	-0.878380225	0.003733	0.035294932
ENSG00000228683	AC016820.1	long_non_coding	-0.981896806	0.003751354	0.035399894
ENSG00000253944	AC027117.2	long_non_coding	-0.686380401	0.003748964	0.035399894
ENSG00000224272	AC131097.3	long_non_coding	0.754283426	0.003759701	0.035465148
ENSG00000228944	AC004485.1	long_non_coding	-1.35484107	0.003813826	0.035822515
ENSG00000261462	AC004023.1	long_non_coding	-1.090127007	0.0038323	0.035919572
ENSG00000249790	AC092490.1	long_non_coding	0.783679536	0.003841837	0.03596312
ENSG00000240707	LINC01168	long_non_coding	0.823263941	0.003877101	0.036139859
ENSG00000260209	AP000842.3	long_non_coding	0.88150214	0.003971205	0.036814797
ENSG00000232949	AC002480.3	long_non_coding	-0.721705132	0.004011844	0.03709022
ENSG00000232079	LINC01697	long_non_coding	-0.600278735	0.00404217	0.037300238
ENSG00000242048	AC093583.1	long_non_coding	-0.705014071	0.004074644	0.037513591
ENSG00000242686	AC107464.1	long_non_coding	-0.771446845	0.004085131	0.037560969
ENSG00000253361	AC069120.1	long_non_coding	-0.719432684	0.004171214	0.038187515
ENSG00000253420	AC103853.1	long_non_coding	1.083758665	0.004176759	0.038214485
ENSG00000233387	AL121748.1	long_non_coding	-0.890264448	0.00420277	0.038380833
ENSG00000261634	AC026992.2	long_non_coding	-0.705229077	0.004224827	0.038501655
ENSG00000227392	HPN-AS1	long_non_coding	0.612417444	0.004287983	0.038925361
ENSG00000233421	LINC01783	long_non_coding	-0.667695983	0.004374655	0.039468511
ENSG00000189229	AC069277.1	long_non_coding	1.237254185	0.004376313	0.039475399
ENSG00000259142	LINC00644	long_non_coding	-1.196659535	0.004429013	0.03977997
ENSG00000236213	AC006369.1	long_non_coding	-0.734560839	0.004431342	0.039784684
ENSG00000258654	AC026495.1	long_non_coding	-0.826314606	0.004506867	0.04033143
ENSG00000249906	AC006487.1	long_non_coding	-0.780044938	0.004535318	0.040536703
ENSG00000228035	AL512638.2	long_non_coding	-0.797386832	0.00457858	0.040742417
ENSG00000259234	ANKRD34C-AS1	long_non_coding	-1.151804145	0.004590588	0.040823942
ENSG00000250431	AC105914.2	long_non_coding	0.914225908	0.004610744	0.040929339
ENSG00000234215	AC006012.1	long_non_coding	-0.767534321	0.004622822	0.041005817
ENSG00000254319	AC246817.2	long_non_coding	-0.586063193	0.004713458	0.041564726
ENSG00000257226	AC079584.2	long_non_coding	-0.890719991	0.004726842	0.041641134
ENSG00000260412	AL353746.1	long_non_coding	-0.777284875	0.004778633	0.041994259
ENSG00000244383	FAM3D-AS1	long_non_coding	0.85268288	0.004834498	0.042335928
ENSG00000229609	LINC01079	long_non_coding	-1.216138806	0.00494597	0.043055702
ENSG00000237346	AL080313.2	long_non_coding	-1.290866787	0.004951439	0.043077816
ENSG00000179840	PIK3CD-AS1	long_non_coding	-0.586376839	0.004992045	0.043311515
ENSG00000179676	LINC00305	long_non_coding	-1.036436658	0.005061353	0.043792265
ENSG00000228705	LINC00659	long_non_coding	0.780083157	0.005080484	0.043888931
ENSG00000258807	AL359237.1	long_non_coding	1.078160516	0.005125266	0.044215188
ENSG00000224177	LINC00570	long_non_coding	0.615456155	0.005293191	0.045291891
ENSG00000254275	LINC00824	long_non_coding	-0.751978097	0.005347385	0.045640538
ENSG00000248801	C8orf34-AS1	long_non_coding	-0.889321226	0.005480549	0.046471431
ENSG00000229228	LINC00582	long_non_coding	-0.651754704	0.005496773	0.046555311
ENSG00000259219	AC084855.2	long_non_coding	-0.594870255	0.005567899	0.046995326
ENSG00000260913	LINC01254	long_non_coding	1.511864889	0.005644208	0.047461121
ENSG00000256151	ADGRD1-AS1	long_non_coding	-0.806973696	0.005692347	0.047735217
ENSG00000229719	MIR194-2HG	long_non_coding	0.644253189	0.005739532	0.047975475
ENSG00000236842	AC010997.2	long_non_coding	-0.87496656	0.005786845	0.048243388
ENSG00000236525	AC007278.2	long_non_coding	-0.812472492	0.005792518	0.048253641
ENSG00000227906	SNAP25-AS1	long_non_coding	-0.602535186	0.005858315	0.048560314
ENSG00000225916	AC007879.2	long_non_coding	0.655131064	0.005901605	0.048766999
ENSG00000232606	LINC01412	long_non_coding	-0.679755269	0.005904663	0.048783133
ENSG00000253891	AC023202.1	long_non_coding	-0.82222167	0.005906997	0.048793273

Supplementary Table 2. prognostic DElncRNAs in the TCGA-PAAD cohort

ENSEMBL	symbol	HR	upper95	lower95	p-value
ENSG00000122043	LINC00544	0.496	0.769	0.32	0.0014
ENSG00000177335	C8orf31	1.68	2.595	1.086	0.0183
ENSG00000197291	RAMP2-AS1	0.621	0.961	0.402	0.031
ENSG00000204110	LINC02520	0.62	0.957	0.402	0.0292
ENSG00000205056	LINC02397	0.619	0.958	0.399	0.0296
ENSG00000213468	FIRRE	1.8	2.794	1.16	0.0078
ENSG00000223511	AL683807.1	0.617	0.962	0.395	0.0316
ENSG00000224387	AL139246.1	0.612	0.948	0.395	0.0265
ENSG00000225472	AL136366.1	0.483	0.753	0.31	0.00104
ENSG00000225756	DBH-AS1	0.629	0.975	0.405	0.0367
ENSG00000227155	AL161725.1	0.624	0.964	0.404	0.0321
ENSG00000227744	LINC01940	2.05	3.203	1.316	0.00122
ENSG00000228835	AC012123.1	0.625	0.97	0.403	0.0345
ENSG00000229393	AL139246.4	0.592	0.922	0.38	0.0192
ENSG00000229425	AJ009632.2	0.637	0.987	0.411	0.0418
ENSG00000230061	TRPM2-AS	0.639	0.992	0.411	0.0442
ENSG00000231465	AL359636.2	1.97	3.083	1.258	0.00258
ENSG00000233515	LINC01518	1.67	2.597	1.07	0.0225
ENSG00000236081	ELFN1-AS1	1.68	2.61	1.079	0.0203
ENSG00000236901	MIR600HG	0.543	0.846	0.348	0.00611
ENSG00000237914	SIRPG-AS1	0.639	0.989	0.413	0.0429
ENSG00000237928	NFIA-AS2	0.638	0.985	0.413	0.0408
ENSG00000241475	AL160408.4	1.82	2.829	1.165	0.00755
ENSG00000241666	AL031733.2	0.461	0.72	0.295	5.00E-04
ENSG00000245164	LINC00861	0.622	0.96	0.402	0.0305
ENSG00000247095	MIR210HG	1.57	2.435	1.016	0.0408
ENSG00000247982	LINC00926	0.581	0.903	0.374	0.0146
ENSG00000249906	AC006487.1	0.559	0.868	0.359	0.00871

ENSG00000253420	AC103853.1	1.64	2.532	1.063	0.0238
ENSG00000253522	MIR3142HG	0.498	0.777	0.319	0.00172
ENSG00000253552	HOXA-AS2	0.632	0.983	0.406	0.0403
ENSG00000256427	AC010175.1	0.544	0.844	0.35	0.00588
ENSG00000258876	TGFB3-AS1	1.54	2.366	0.998	0.0498
ENSG00000259070	LINC00639	0.545	0.844	0.352	0.00576
ENSG00000259238	AC092755.2	1.6	2.466	1.039	0.0314
ENSG00000259771	AC092756.1	1.89	2.93	1.221	0.00374
ENSG00000259881	AC092384.2	0.596	0.925	0.384	0.0196
ENSG00000259954	IL21R-AS1	0.592	0.918	0.382	0.0179
ENSG00000260719	AC009133.3	0.628	0.972	0.406	0.0354
ENSG00000260902	LINC02011	1.77	2.778	1.131	0.0113
ENSG00000261416	AC012645.3	0.541	0.84	0.349	0.0054
ENSG00000261604	AC114947.2	0.625	0.967	0.404	0.0333
ENSG00000261839	AL358933.1	0.579	0.896	0.375	0.0132

Supplementary Table 3. The clinical features of the training set, the testing set, and the TCGA-PAAD cohort

Covariates	Type	Total(n=176)	Training set(n=88)	Testing set(n=88)	P value
Age(n/%)					1
	＜60	54(60.68)	27(30.68)	27(30.68)	
	≥60	122(69.32)	61(69.32)	61(69.32)	
Gender(n/%)					0.173
	Female	80(45.45)	45(51.14)	35(39.77)	
	Male	96(54.55)	43(48.86)	53(60.23)	
Tumor Stage(n/%)					0.135
	Stage Ⅰ-Ⅱ	166(94.32)	81(92.05)	85(96.59)	
	Stage Ⅲ-Ⅳ	7(3.98)	6(6.82)	1(1.14)	
	Unknown	3(1.70)	1(1.14)	2(2.27)	
T Stage(n/%)					0.981
	T1-2	31(17.61)	16(18.18)	15(17.05)	
	T3-4	143(81.25)	71(80.68)	72(81.82)	
	Unknown	2(1.14)	1(1.14)	1(1.14)	
M Stage(n/%)					0.043
	M0	79(44.89)	43(48.86)	36(40.91)	
	M1	4(2.27)	4(4.55)	0(0.00)	
	Unknown	93(52.84)	41(46.59)	52(59.09)	
N Stage(n/%)					0.231
	N0	49(27.84)	21(23.86)	28(31.82)	
	N1	122(69.32)	63(71.59)	59(67.05)	
	Unknown	5(2.84)	4(4.55)	1(1.14)	
family_history_of_cancer (n/%)					0.248
	YES	62(35.23)	26(29.55)	36(40.91)	
	NO	47(26.70)	27(30.68)	20(22.73)	
	Unknown	67(38.07)	35(39.77)	32(36.36)	
history_of_chronic_pancreatitis (n/%)					0.414
	YES	13(7.39)	8(9.09)	5(5.68)	
	NO	127(72.16)	65(73.86)	62(70.45)	
	Unknown	36(20.45)	15(17.05)	21(23.86)	
history_of_diabetes (n/%)					0.608
	YES	38(21.59)	20(22.73)	18(20.45)	
	NO	107(60.80)	55(62.50)	52(59.09)	
	Unknown	31(17.61)	13(14.77)	18(20.45)	
radiation_therapy (n/%)					0.862
	YES	37(21.02)	18(20.45)	19(21.59)	
	NO	100(56.82)	49(55.68)	51(57.95)	
	Unknown	39(22.16)	21(23.86)	18(20.45)	
alcohol_history.exposures (n/%)					0.63
	YES	100(56.82)	47(53.41)	53(60.23)	
	NO	64(36.36)	35(39.77)	29(32.95)	
	Unknown	12(6.82)	6(6.82)	6(6.82)	
neoplasm_histologic_grade (n/%)					0.353
	G1-G2	124(70.45)	62(70.45)	62(70.45)	
	G3-G4	50(28.41)	26(29.55)	24(27.27)	
	Unknown	2(1.14)	0(0.00)	2(2.27)	

Supplementary Table 4. Mulvariate analysis of fourteen selected lncRNAs in the training set

Gene	lncipediaTranscriptID	coef	HR	upper95	lower95	p-value
ENSG00000177335	C8orf31	0.38941	1.68	2.595	1.086	0.0183
ENSG00000213468	FIRRE	0.12915	1.8	2.794	1.16	0.0078
ENSG00000227744	LINC01940	0.16976	2.05	3.203	1.316	0.00122
ENSG00000229393	AL139246.4	0.1125	0.592	0.922	0.38	0.0192
ENSG00000230061	TRPM2-AS	-0.0176	0.639	0.992	0.411	0.0442
ENSG00000233515	LINC01518	0.1546	1.67	2.597	1.07	0.0225
ENSG00000236081	ELFN1-AS1	0.08236	1.68	2.61	1.079	0.0203
ENSG00000236901	MIR600HG	-0.2317	0.543	0.846	0.348	0.00611
ENSG00000253420	AC103853.1	0.23194	1.64	2.532	1.063	0.0238
ENSG00000253522	MIR3142HG	-0.1095	0.498	0.777	0.319	0.00172
ENSG00000253552	HOXA-AS2	-0.1466	0.632	0.983	0.406	0.0403
ENSG00000256427	AC010175.1	-0.0509	0.544	0.844	0.35	0.00588
ENSG00000259771	AC092756.1	0.26826	1.89	2.93	1.221	0.00374
ENSG00000261604	AC114947.2	-0.2521	0.625	0.967	0.404	0.0333

pplementary Table 5. Differentially expressed genes between high-risk and low-risk groups in the TCGA-PAAD coh

symbol	group	logFC	PValue	FDR	threshold
C8orf31	long_non_coding	2.205594779	3.30E-25	1.00E-21	Up
IL20RB	protein_coding	2.474346677	1.10E-20	5.20E-18	Up
TNNT1	protein_coding	2.520687026	1.59E-19	5.26E-17	Up
LINC00941	long_non_coding	1.621192988	1.91E-16	2.16E-14	Up
AP1S3	protein_coding	0.990205199	2.40E-16	2.66E-14	Up
MBOAT2	protein_coding	0.954680636	4.01E-16	4.22E-14	Up
SEMA4B	protein_coding	0.984080409	4.27E-16	4.41E-14	Up
PCDH1	protein_coding	0.830730321	4.40E-16	4.48E-14	Up
KRT6A	protein_coding	3.011562707	6.97E-16	6.45E-14	Up
LAMA3	protein_coding	1.321910005	1.27E-15	1.10E-13	Up
PTPRR	protein_coding	1.522667636	1.96E-15	1.60E-13	Up
FLNB	protein_coding	0.997510245	4.38E-15	3.23E-13	Up
ITGA6	protein_coding	0.87961586	6.48E-15	4.51E-13	Up
PERP	protein_coding	0.807384158	1.98E-14	1.22E-12	Up
SLK	protein_coding	0.61685928	2.07E-14	1.26E-12	Up
S100A2	protein_coding	2.537762566	2.55E-14	1.53E-12	Up
ASPH	protein_coding	0.879545214	4.59E-14	2.56E-12	Up
TLDC1	protein_coding	0.587059429	7.06E-14	3.82E-12	Up
LDHA	protein_coding	0.75693214	7.81E-14	4.16E-12	Up
TMBIM1	protein_coding	0.703036894	8.16E-14	4.33E-12	Up
SMURF1	protein_coding	0.638788227	9.28E-14	4.82E-12	Up
FAM83A	protein_coding	2.215510653	1.00E-13	5.09E-12	Up
EFNB1	protein_coding	0.92855557	1.07E-13	5.39E-12	Up
PDE4C	protein_coding	1.518769026	1.24E-13	6.11E-12	Up
MET	protein_coding	1.08117744	1.60E-13	7.68E-12	Up
CNNM4	protein_coding	0.68108839	1.80E-13	8.43E-12	Up
DSG3	protein_coding	2.372579669	2.32E-13	1.06E-11	Up
CDCP1	protein_coding	0.852519898	2.52E-13	1.13E-11	Up
EPB41L1	protein_coding	0.652804742	2.66E-13	1.18E-11	Up
ABHD17C	protein_coding	1.061142869	2.82E-13	1.24E-11	Up
MGLL	protein_coding	0.832075083	4.21E-13	1.74E-11	Up
LMO7	protein_coding	0.825272952	4.41E-13	1.80E-11	Up
PLCD3	protein_coding	0.980914735	6.37E-13	2.49E-11	Up
SLC2A1	protein_coding	1.298930097	6.46E-13	2.52E-11	Up
SERPINB5	protein_coding	1.530772889	1.08E-12	3.94E-11	Up
FAM3C	protein_coding	0.656007797	1.17E-12	4.22E-11	Up
HIST1H2BD	protein_coding	1.081871458	1.25E-12	4.49E-11	Up
ERO1A	protein_coding	0.863699531	1.40E-12	4.95E-11	Up
PGK1	protein_coding	0.600884479	1.40E-12	4.95E-11	Up
MALL	protein_coding	1.211027379	1.63E-12	5.69E-11	Up
NET1	protein_coding	0.769999674	1.66E-12	5.77E-11	Up
LPCAT4	protein_coding	0.993965252	1.68E-12	5.79E-11	Up
RHOF	protein_coding	0.917463212	1.96E-12	6.72E-11	Up
FOXL1	protein_coding	1.314514497	2.51E-12	8.37E-11	Up
DKK1	protein_coding	1.819209342	3.09E-12	1.01E-10	Up
PKM	protein_coding	0.613784498	3.16E-12	1.03E-10	Up
SOWAHC	protein_coding	0.622113855	3.34E-12	1.08E-10	Up
APCDD1L	protein_coding	1.732577792	3.56E-12	1.14E-10	Up
FAM83H	protein_coding	0.865749199	3.91E-12	1.24E-10	Up

PLEC	protein_coding	0.865187637	4.23E-12	1.33E-10	Up
BCL2L1	protein_coding	0.588095716	4.53E-12	1.40E-10	Up
IFI27	protein_coding	1.229291895	4.72E-12	1.45E-10	Up
AFAP1L2	protein_coding	0.782443194	4.75E-12	1.46E-10	Up
COL17A1	protein_coding	1.776790662	4.89E-12	1.50E-10	Up
CA12	protein_coding	1.42560505	5.30E-12	1.60E-10	Up
PRKCI	protein_coding	0.715099363	7.05E-12	2.09E-10	Up
CMTM1	protein_coding	0.82014666	7.51E-12	2.21E-10	Up
ITGB6	protein_coding	1.375789862	8.36E-12	2.43E-10	Up
CHMP4C	protein_coding	0.735032584	1.06E-11	3.00E-10	Up
ECT2	protein_coding	0.811554102	1.11E-11	3.13E-10	Up
AHNAK2	protein_coding	1.28368002	1.29E-11	3.52E-10	Up
HIST1H2BK	protein_coding	0.910851344	1.31E-11	3.59E-10	Up
PKP3	protein_coding	0.981314856	1.37E-11	3.72E-10	Up
PPARG	protein_coding	1.111208724	1.55E-11	4.13E-10	Up
MMP28	protein_coding	1.131178822	1.69E-11	4.46E-10	Up
ITGA3	protein_coding	0.926057911	1.85E-11	4.85E-10	Up
SLC35F2	protein_coding	0.819773537	1.98E-11	5.15E-10	Up
HAS3	protein_coding	1.407321306	2.03E-11	5.26E-10	Up
HMGA2	protein_coding	1.781271648	2.09E-11	5.40E-10	Up
P2RY2	protein_coding	1.040454784	2.43E-11	6.13E-10	Up
PYGB	protein_coding	0.79037353	2.68E-11	6.67E-10	Up
ARHGAP32	protein_coding	0.640578113	3.01E-11	7.39E-10	Up
PITX1	protein_coding	1.42894632	3.09E-11	7.54E-10	Up
TNFRSF21	protein_coding	0.683742957	3.63E-11	8.69E-10	Up
FAM3C2	pseudogene	0.78566605	5.84E-11	1.34E-09	Up
CASC8	long_non_coding	1.339960815	6.10E-11	1.40E-09	Up
ANXA2P2	pseudogene	0.756605641	6.36E-11	1.45E-09	Up
LY6E	protein_coding	1.018976819	7.22E-11	1.63E-09	Up
LAMB3	protein_coding	1.207428917	7.53E-11	1.68E-09	Up
EPHX4	protein_coding	1.042835842	7.76E-11	1.72E-09	Up
HK2	protein_coding	1.056958709	7.85E-11	1.74E-09	Up
TM4SF1	protein_coding	0.929512673	8.24E-11	1.81E-09	Up
PLAC8	protein_coding	1.241202536	8.76E-11	1.90E-09	Up
SAMD9	protein_coding	0.818543982	9.32E-11	2.00E-09	Up
TRIP10	protein_coding	0.715849059	9.72E-11	2.08E-09	Up
IRAK2	protein_coding	0.84465217	1.10E-10	2.33E-09	Up
SMAD3	protein_coding	0.684639389	1.13E-10	2.39E-09	Up
ANLN	protein_coding	1.062239539	1.26E-10	2.65E-09	Up
TMC7	protein_coding	0.963499897	1.28E-10	2.67E-09	Up
BAIAP2L1	protein_coding	0.830228546	1.28E-10	2.67E-09	Up
INF2	protein_coding	0.743432831	1.28E-10	2.67E-09	Up
B3GNT5	protein_coding	0.698584729	1.66E-10	3.36E-09	Up
FGD6	protein_coding	0.740923761	1.66E-10	3.36E-09	Up
MCU	protein_coding	0.691394005	1.76E-10	3.52E-09	Up
H1F0	protein_coding	0.626949308	1.80E-10	3.60E-09	Up
AC002384.1	long_non_coding	1.579961878	1.83E-10	3.65E-09	Up
ABTB2	protein_coding	0.787045726	1.97E-10	3.92E-09	Up
KCNK1	protein_coding	0.873194267	2.44E-10	4.70E-09	Up
RIOK3	protein_coding	0.607890688	2.46E-10	4.73E-09	Up
CD58	protein_coding	0.810024703	2.61E-10	4.98E-09	Up
ZNF488	protein_coding	1.258009817	2.79E-10	5.27E-09	Up

ANXA2	protein_coding	0.727245741	3.00E-10	5.63E-09	Up
E2F7	protein_coding	1.058431641	3.28E-10	6.09E-09	Up
OAS1	protein_coding	0.903465252	3.31E-10	6.12E-09	Up
ESRP1	protein_coding	0.746188146	3.69E-10	6.78E-09	Up
EZR	protein_coding	0.679838578	4.10E-10	7.44E-09	Up
ITGA2	protein_coding	0.861433166	4.12E-10	7.46E-09	Up
EPHA2	protein_coding	1.020780243	4.43E-10	7.95E-09	Up
EREG	protein_coding	1.778325558	4.66E-10	8.30E-09	Up
AMIGO2	protein_coding	0.93732201	5.74E-10	1.00E-08	Up
TCF7L2	protein_coding	0.630018449	5.80E-10	1.01E-08	Up
PARD6B	protein_coding	0.728704282	6.76E-10	1.16E-08	Up
RARG	protein_coding	0.612713437	7.04E-10	1.21E-08	Up
GREB1L	protein_coding	1.169602625	7.06E-10	1.21E-08	Up
DDX60	protein_coding	0.804583088	7.45E-10	1.27E-08	Up
MYOF	protein_coding	0.743168992	8.15E-10	1.37E-08	Up
SLC5A3	protein_coding	0.658116283	8.41E-10	1.42E-08	Up
XDH	protein_coding	1.228952935	9.22E-10	1.53E-08	Up
ADAM9	protein_coding	0.739418792	1.02E-09	1.67E-08	Up
ASAP2	protein_coding	0.80181349	1.16E-09	1.88E-08	Up
CDH3	protein_coding	1.089349372	1.22E-09	1.97E-08	Up
JUP	protein_coding	0.685082909	1.23E-09	1.98E-08	Up
MROH6	protein_coding	1.120703036	1.25E-09	2.01E-08	Up
TUFT1	protein_coding	0.675523227	1.32E-09	2.10E-08	Up
PTK6	protein_coding	1.107381937	1.51E-09	2.38E-08	Up
HIST1H3H	protein_coding	1.304830943	1.56E-09	2.44E-08	Up
MYEOV	protein_coding	1.368876848	1.60E-09	2.50E-08	Up
LFNG	protein_coding	0.856935668	1.63E-09	2.54E-08	Up
FSCN1	protein_coding	0.844469179	1.77E-09	2.73E-08	Up
DOK4	protein_coding	0.600421931	1.83E-09	2.82E-08	Up
BIK	protein_coding	0.939056789	1.85E-09	2.84E-08	Up
ANO1	protein_coding	0.816996069	2.02E-09	3.06E-08	Up
HIST1H4I	protein_coding	0.839202564	2.16E-09	3.25E-08	Up
SDCBP2	protein_coding	0.956572279	2.22E-09	3.33E-08	Up
LAMC2	protein_coding	1.127452519	2.33E-09	3.47E-08	Up
INPP4B	protein_coding	0.734765101	2.33E-09	3.47E-08	Up
OCIAD2	protein_coding	0.687190416	2.41E-09	3.58E-08	Up
ERBB2	protein_coding	0.667032034	2.47E-09	3.66E-08	Up
PLEK2	protein_coding	1.027460416	2.54E-09	3.75E-08	Up
KRT16	protein_coding	1.8223879	2.57E-09	3.78E-08	Up
RNF39	protein_coding	1.066742023	2.60E-09	3.82E-08	Up
PPP1R3G	protein_coding	0.900125285	2.91E-09	4.22E-08	Up
GJB3	protein_coding	1.143452719	2.98E-09	4.31E-08	Up
SH2D3A	protein_coding	0.913693846	3.06E-09	4.40E-08	Up
HS3ST1	protein_coding	0.979612187	3.25E-09	4.66E-08	Up
ARNTL2	protein_coding	0.736093862	3.30E-09	4.72E-08	Up
OASL	protein_coding	1.157221197	3.47E-09	4.92E-08	Up
B3GNT3	protein_coding	0.882552354	3.76E-09	5.26E-08	Up
TMEM159	protein_coding	0.598246969	3.81E-09	5.32E-08	Up
CEP55	protein_coding	0.85529999	3.92E-09	5.46E-08	Up
BCAR3	protein_coding	0.679630019	3.96E-09	5.50E-08	Up
LINC00857	long_non_coding	0.843672252	4.12E-09	5.72E-08	Up
NT5E	protein_coding	0.906607769	4.21E-09	5.84E-08	Up

FRMD5	protein_coding	0.801185278	4.34E-09	5.96E-08	Up
SEC14L2	protein_coding	0.727707567	4.38E-09	6.00E-08	Up
PLCB3	protein_coding	0.637926401	4.71E-09	6.41E-08	Up
DNAH3	protein_coding	1.027884749	4.93E-09	6.70E-08	Up
P4HA1	protein_coding	0.693140847	4.98E-09	6.75E-08	Up
TBX6	protein_coding	1.038764654	5.35E-09	7.19E-08	Up
LINC01133	long_non_coding	1.364146719	5.35E-09	7.19E-08	Up
SPIN4	protein_coding	0.647715962	5.37E-09	7.20E-08	Up
UGT1A10	protein_coding	1.587085263	5.60E-09	7.48E-08	Up
F2RL1	protein_coding	0.766051732	5.69E-09	7.58E-08	Up
CDK1	protein_coding	0.742583233	6.15E-09	8.10E-08	Up
KPNA7	protein_coding	1.131249715	6.31E-09	8.27E-08	Up
PRELID2	protein_coding	0.681518777	6.53E-09	8.53E-08	Up
IQANK1	protein_coding	0.874954669	6.96E-09	9.03E-08	Up
GJB5	protein_coding	1.482337289	7.14E-09	9.22E-08	Up
CTSV	protein_coding	1.114755559	7.53E-09	9.69E-08	Up
KLF5	protein_coding	0.874675612	7.55E-09	9.71E-08	Up
LDLR	protein_coding	0.808673165	8.34E-09	1.07E-07	Up
WNT7B	protein_coding	1.01557988	8.63E-09	1.10E-07	Up
RHOD	protein_coding	0.942229205	8.72E-09	1.11E-07	Up
EGLN3	protein_coding	1.164152444	8.85E-09	1.13E-07	Up
IL1R2	protein_coding	1.413272974	9.09E-09	1.15E-07	Up
KCP	protein_coding	1.210540533	9.30E-09	1.18E-07	Up
HIST1H2BC	protein_coding	1.003430951	9.33E-09	1.18E-07	Up
CKLF	protein_coding	0.603940269	9.33E-09	1.18E-07	Up
IGF2BP2	protein_coding	0.85313997	9.51E-09	1.20E-07	Up
CD55	protein_coding	0.885042305	1.09E-08	1.36E-07	Up
SLC6A8	protein_coding	0.977209843	1.17E-08	1.44E-07	Up
NOG	protein_coding	1.058323419	1.31E-08	1.60E-07	Up
NGEF	protein_coding	0.896946213	1.32E-08	1.60E-07	Up
ADAMTSL5	protein_coding	0.914387652	1.37E-08	1.66E-07	Up
LGALS3	protein_coding	0.751347674	1.39E-08	1.68E-07	Up
GPRC5A	protein_coding	1.146320437	1.43E-08	1.73E-07	Up
DDIT4	protein_coding	0.798513632	1.47E-08	1.77E-07	Up
PLS1	protein_coding	0.778858735	1.48E-08	1.78E-07	Up
KRT7	protein_coding	1.133566243	1.52E-08	1.82E-07	Up
VILL	protein_coding	1.055582198	1.54E-08	1.84E-07	Up
CLTB	protein_coding	0.686253863	1.58E-08	1.89E-07	Up
KRT19	protein_coding	0.981410482	1.60E-08	1.91E-07	Up
RRAS	protein_coding	0.61250193	1.63E-08	1.94E-07	Up
HIST1H2AC	protein_coding	0.747945732	1.76E-08	2.07E-07	Up
HSPA6	protein_coding	1.342483033	1.84E-08	2.16E-07	Up
HELZ2	protein_coding	0.592637256	1.91E-08	2.24E-07	Up
FOXQ1	protein_coding	0.922393885	2.00E-08	2.34E-07	Up
P3H2	protein_coding	0.873608991	2.10E-08	2.44E-07	Up
MST1R	protein_coding	0.982509598	2.11E-08	2.45E-07	Up
LETM2	protein_coding	0.757967012	2.13E-08	2.47E-07	Up
S100A4	protein_coding	1.186785063	2.23E-08	2.58E-07	Up
GPR39	protein_coding	0.685590405	2.29E-08	2.63E-07	Up
IRF6	protein_coding	0.667233802	2.33E-08	2.67E-07	Up
SLC13A5	protein_coding	1.702697157	2.44E-08	2.79E-07	Up
IGF2BP3	protein_coding	1.381106021	2.45E-08	2.79E-07	Up

TMPRSS4	protein_coding	1.159233915	2.50E-08	2.84E-07	Up
LPCAT2	protein_coding	0.700042225	2.50E-08	2.85E-07	Up
AC025594.2	protein_coding	1.039791488	2.74E-08	3.07E-07	Up
CSTB	protein_coding	0.662051308	2.86E-08	3.19E-07	Up
SH3PXD2A-AS1	long_non_coding	1.28535481	2.87E-08	3.19E-07	Up
ZNF185	protein_coding	0.860229899	2.96E-08	3.27E-07	Up
WNT7A	protein_coding	1.453554426	3.38E-08	3.68E-07	Up
BEAN1	protein_coding	1.087482925	3.45E-08	3.75E-07	Up
ASPHD2	protein_coding	0.811027237	3.51E-08	3.80E-07	Up
KRT7-AS	long_non_coding	1.111879013	3.52E-08	3.81E-07	Up
FAM213B	protein_coding	0.587954522	3.52E-08	3.81E-07	Up
EFNA3	protein_coding	0.993442687	3.72E-08	4.00E-07	Up
TP63	protein_coding	1.404578066	3.75E-08	4.02E-07	Up
KLHL30	protein_coding	0.982780588	3.82E-08	4.09E-07	Up
S100A10	protein_coding	0.682034961	3.99E-08	4.25E-07	Up
MECOM	protein_coding	0.751353966	4.03E-08	4.28E-07	Up
LY6D	protein_coding	2.160965859	4.04E-08	4.30E-07	Up
FA2H	protein_coding	0.953351572	4.40E-08	4.64E-07	Up
ALDH3B1	protein_coding	0.760126177	4.50E-08	4.73E-07	Up
TRIM7	protein_coding	1.033486153	4.60E-08	4.83E-07	Up
OSBPL3	protein_coding	0.617893858	4.81E-08	5.04E-07	Up
RNF223	protein_coding	1.095743739	4.81E-08	5.04E-07	Up
ITGB4	protein_coding	0.912741908	4.86E-08	5.08E-07	Up
PLK1	protein_coding	0.716701189	4.94E-08	5.15E-07	Up
CDA	protein_coding	1.122534556	5.02E-08	5.21E-07	Up
AP005233.2	long_non_coding	1.44642012	5.04E-08	5.23E-07	Up
POU2F3	protein_coding	1.159388029	5.12E-08	5.28E-07	Up
APOL1	protein_coding	0.780837498	5.19E-08	5.35E-07	Up
KLHDC7B	protein_coding	1.530123319	5.30E-08	5.46E-07	Up
PRC1	protein_coding	0.649406487	5.37E-08	5.52E-07	Up
OR7E14P	pseudogene	0.74756563	5.40E-08	5.54E-07	Up
XRCC4	protein_coding	0.734411606	5.91E-08	6.01E-07	Up
REPS2	protein_coding	0.625253311	6.00E-08	6.08E-07	Up
PROM2	protein_coding	1.1075151	6.01E-08	6.09E-07	Up
LRRC8A	protein_coding	0.670632556	6.16E-08	6.22E-07	Up
RAPGEFL1	protein_coding	1.001677068	6.42E-08	6.45E-07	Up
CAV2	protein_coding	0.657385388	6.81E-08	6.80E-07	Up
ARHGAP27	protein_coding	0.652889294	6.86E-08	6.84E-07	Up
PTGES	protein_coding	1.146503744	6.88E-08	6.86E-07	Up
KRT13	protein_coding	1.901193487	7.19E-08	7.13E-07	Up
MAFF	protein_coding	0.616079027	7.25E-08	7.18E-07	Up
STEAP3	protein_coding	0.771352663	7.31E-08	7.22E-07	Up
UHRF1	protein_coding	0.694390197	7.47E-08	7.36E-07	Up
HES2	protein_coding	1.33104628	7.56E-08	7.45E-07	Up
ABLIM3	protein_coding	0.676222844	7.76E-08	7.63E-07	Up
TMEM105	protein_coding	0.994625279	7.77E-08	7.63E-07	Up
NFE2L3	protein_coding	0.612346563	7.88E-08	7.74E-07	Up
SDC1	protein_coding	0.773098052	8.43E-08	8.19E-07	Up
OAS3	protein_coding	0.722807562	8.47E-08	8.21E-07	Up
SRD5A3	protein_coding	0.628527978	8.97E-08	8.67E-07	Up
C1orf116	protein_coding	0.673191255	9.06E-08	8.72E-07	Up
HRH1	protein_coding	0.633336886	9.71E-08	9.29E-07	Up

ADORA2B	protein_coding	0.875324971	9.97E-08	9.53E-07	Up
TSPAN1	protein_coding	0.908774488	1.03E-07	9.84E-07	Up
SMOX	protein_coding	0.608948332	1.07E-07	1.02E-06	Up
PKP1	protein_coding	1.317936655	1.08E-07	1.03E-06	Up
CAPRIN2	protein_coding	0.71859962	1.32E-07	1.23E-06	Up
ABHD2	protein_coding	0.699493775	1.39E-07	1.28E-06	Up
TGFA	protein_coding	0.727188813	1.43E-07	1.31E-06	Up
RHBDL2	protein_coding	0.806067411	1.43E-07	1.31E-06	Up
SDC4	protein_coding	0.635077871	1.44E-07	1.32E-06	Up
PPP1R13L	protein_coding	0.73222895	1.45E-07	1.33E-06	Up
MCM10	protein_coding	0.730196463	1.47E-07	1.34E-06	Up
PLEKHA7	protein_coding	0.618615624	1.57E-07	1.42E-06	Up
PHLDA1	protein_coding	0.599485943	1.60E-07	1.44E-06	Up
MXD1	protein_coding	0.724001324	1.60E-07	1.44E-06	Up
AC245041.2	long_non_coding	1.041534011	1.65E-07	1.48E-06	Up
DUSP4	protein_coding	0.63865993	1.66E-07	1.49E-06	Up
HSPB1	protein_coding	0.637008013	1.79E-07	1.59E-06	Up
MICALCL	protein_coding	0.773871636	1.79E-07	1.59E-06	Up
DSG2	protein_coding	0.635009828	1.79E-07	1.60E-06	Up
NHS	protein_coding	0.668496781	2.04E-07	1.78E-06	Up
MPZL2	protein_coding	0.769210576	2.13E-07	1.85E-06	Up
LIPH	protein_coding	0.823587101	2.17E-07	1.87E-06	Up
CRNDE	long_non_coding	0.825929719	2.22E-07	1.91E-06	Up
ANXA3	protein_coding	0.768182772	2.30E-07	1.97E-06	Up
FERMT1	protein_coding	0.833762927	2.31E-07	1.98E-06	Up
RHPN2	protein_coding	0.718805117	2.45E-07	2.08E-06	Up
GJB4	protein_coding	1.142070587	2.57E-07	2.18E-06	Up
SDR16C5	protein_coding	1.131959783	2.70E-07	2.28E-06	Up
PLAU	protein_coding	0.927435735	2.74E-07	2.31E-06	Up
GRB7	protein_coding	0.69217021	2.74E-07	2.31E-06	Up
HLA-F-AS1	long_non_coding	0.672008581	2.80E-07	2.35E-06	Up
KIF23	protein_coding	0.67578299	3.18E-07	2.62E-06	Up
MAL2	protein_coding	0.715244892	3.26E-07	2.68E-06	Up
PLA2G16	protein_coding	0.663093524	3.29E-07	2.70E-06	Up
GALNT5	protein_coding	1.043825027	3.31E-07	2.72E-06	Up
HKDC1	protein_coding	0.889785117	3.55E-07	2.90E-06	Up
SLC20A1	protein_coding	0.625408632	3.58E-07	2.92E-06	Up
NMB	protein_coding	0.649702825	3.60E-07	2.93E-06	Up
GNA15	protein_coding	0.707195557	3.75E-07	3.03E-06	Up
ITPR3	protein_coding	0.668098797	3.80E-07	3.06E-06	Up
FLNB-AS1	long_non_coding	0.942292155	3.83E-07	3.08E-06	Up
HLA-V	pseudogene	0.927978317	3.91E-07	3.14E-06	Up
ANKRD22	protein_coding	0.89337111	3.94E-07	3.16E-06	Up
HIST1H1C	protein_coding	0.856615494	4.11E-07	3.29E-06	Up
BUB1B	protein_coding	0.617502654	4.18E-07	3.33E-06	Up
CEMIP	protein_coding	1.006189423	4.24E-07	3.37E-06	Up
KCNN4	protein_coding	0.969691313	4.35E-07	3.45E-06	Up
PPP1R1C	protein_coding	0.729040013	4.56E-07	3.59E-06	Up
PRR15	protein_coding	0.799761494	4.64E-07	3.64E-06	Up
KRT17	protein_coding	1.360867057	4.84E-07	3.77E-06	Up
AP002761.4	long_non_coding	0.929743743	4.97E-07	3.87E-06	Up
AFAP1-AS1	long_non_coding	1.302509959	4.98E-07	3.88E-06	Up

CREG2	protein_coding	0.961911451	5.29E-07	4.09E-06	Up
S100A16	protein_coding	0.701454175	5.68E-07	4.36E-06	Up
ALS2CL	protein_coding	0.821707555	6.02E-07	4.60E-06	Up
DLGAP5	protein_coding	0.751659519	6.07E-07	4.63E-06	Up
CDC6	protein_coding	0.66458713	6.23E-07	4.74E-06	Up
HPDL	protein_coding	0.978991809	6.32E-07	4.81E-06	Up
FGFBP1	protein_coding	1.399963113	6.38E-07	4.84E-06	Up
IGFBP3	protein_coding	0.75058738	6.70E-07	5.05E-06	Up
SCUBE3	protein_coding	1.133816266	7.03E-07	5.28E-06	Up
HJURP	protein_coding	0.73976674	7.03E-07	5.28E-06	Up
SH2D4A	protein_coding	0.657555958	7.53E-07	5.59E-06	Up
SLC16A3	protein_coding	0.802138498	7.68E-07	5.69E-06	Up
UNC93B1	protein_coding	0.612728217	7.87E-07	5.80E-06	Up
CGN	protein_coding	0.654133303	7.96E-07	5.86E-06	Up
SLC39A4	protein_coding	0.901414152	8.06E-07	5.92E-06	Up
LY75	protein_coding	0.632447974	8.09E-07	5.93E-06	Up
S100A11	protein_coding	0.66643759	8.33E-07	6.08E-06	Up
PLEKHN1	protein_coding	0.906626305	8.44E-07	6.14E-06	Up
SPTBN2	protein_coding	0.628405591	8.56E-07	6.23E-06	Up
JPT1	protein_coding	0.608052699	8.58E-07	6.24E-06	Up
TNFRSF10A	protein_coding	0.633892309	8.71E-07	6.33E-06	Up
FER1L4	pseudogene	1.200935567	8.76E-07	6.35E-06	Up
LINC00346	long_non_coding	0.763165161	8.91E-07	6.45E-06	Up
TINAGL1	protein_coding	0.685774968	9.01E-07	6.50E-06	Up
TRIM54	protein_coding	1.129808805	9.09E-07	6.56E-06	Up
KIF20A	protein_coding	0.677625537	9.19E-07	6.61E-06	Up
CKAP2L	protein_coding	0.692684957	9.36E-07	6.71E-06	Up
TONSL	protein_coding	0.632493978	9.59E-07	6.85E-06	Up
ESPN	protein_coding	1.126208556	9.71E-07	6.92E-06	Up
CABP4	protein_coding	0.739606878	1.03E-06	7.33E-06	Up
EFNA5	protein_coding	0.649155318	1.06E-06	7.52E-06	Up
ANGPTL4	protein_coding	1.122095359	1.10E-06	7.72E-06	Up
MYH14	protein_coding	0.642195868	1.12E-06	7.84E-06	Up
BHLHE40	protein_coding	0.63347789	1.14E-06	7.96E-06	Up
SERINC2	protein_coding	0.739878909	1.15E-06	8.05E-06	Up
NPR3	protein_coding	0.966353918	1.19E-06	8.31E-06	Up
LINC01559	long_non_coding	1.306840174	1.20E-06	8.37E-06	Up
SMAGP	protein_coding	0.607948249	1.23E-06	8.56E-06	Up
KIF18A	protein_coding	0.697456968	1.23E-06	8.57E-06	Up
SLC16A1	protein_coding	0.617123196	1.28E-06	8.85E-06	Up
LOXL2	protein_coding	0.837049172	1.29E-06	8.89E-06	Up
PRSS22	protein_coding	0.812378889	1.37E-06	9.43E-06	Up
IL1RAP	protein_coding	0.648425202	1.37E-06	9.43E-06	Up
AC090515.4	long_non_coding	0.764232704	1.46E-06	9.98E-06	Up
TOR4A	protein_coding	0.60404243	1.47E-06	1.00E-05	Up
DCBLD2	protein_coding	0.868709625	1.54E-06	1.05E-05	Up
PLBD1	protein_coding	0.693121115	1.56E-06	1.06E-05	Up
PLAT	protein_coding	0.965975677	1.65E-06	1.11E-05	Up
TMEM92	protein_coding	0.84146401	1.66E-06	1.12E-05	Up
CAPN5	protein_coding	0.734542046	1.66E-06	1.12E-05	Up
IL1RN	protein_coding	0.904449086	1.67E-06	1.13E-05	Up
AC068580.3	long_non_coding	0.705027052	1.68E-06	1.13E-05	Up

HIST3H2A	protein_coding	0.70419415	1.70E-06	1.14E-05	Up
AK4	protein_coding	0.762685309	1.76E-06	1.18E-05	Up
FOSL1	protein_coding	0.914316799	1.78E-06	1.19E-05	Up
TRIM29	protein_coding	1.195312491	1.81E-06	1.21E-05	Up
PTPRU	protein_coding	0.677615829	1.87E-06	1.24E-05	Up
NR1I2	protein_coding	1.231267168	1.90E-06	1.26E-05	Up
B4GALNT3	protein_coding	0.762933362	1.90E-06	1.26E-05	Up
RTN4R	protein_coding	0.724295382	1.92E-06	1.27E-05	Up
HMGA1	protein_coding	0.740038333	1.94E-06	1.28E-05	Up
MISP	protein_coding	0.777374787	1.97E-06	1.30E-05	Up
ARL14	protein_coding	1.099416566	2.06E-06	1.35E-05	Up
SLC45A3	protein_coding	0.772126416	2.09E-06	1.37E-05	Up
NR1D1	protein_coding	0.647968319	2.14E-06	1.39E-05	Up
NAGS	protein_coding	0.684681806	2.17E-06	1.41E-05	Up
BMP4	protein_coding	0.792677986	2.22E-06	1.44E-05	Up
PTPRH	protein_coding	0.678509888	2.24E-06	1.45E-05	Up
AC093162.2	pseudogene	0.705716609	2.27E-06	1.47E-05	Up
MKI67	protein_coding	0.70584078	2.32E-06	1.50E-05	Up
CELSR1	protein_coding	0.651270352	2.33E-06	1.50E-05	Up
TPX2	protein_coding	0.713001103	2.46E-06	1.58E-05	Up
AGRN	protein_coding	0.625482999	2.52E-06	1.61E-05	Up
KNL1	protein_coding	0.60795727	2.53E-06	1.62E-05	Up
WTAPP1	pseudogene	0.992735761	2.53E-06	1.62E-05	Up
TFAP2A	protein_coding	0.853738978	2.58E-06	1.65E-05	Up
ACSL5	protein_coding	0.767626479	2.64E-06	1.68E-05	Up
MTMR11	protein_coding	0.729737719	2.72E-06	1.72E-05	Up
TNFSF9	protein_coding	1.019593523	2.72E-06	1.72E-05	Up
GPR87	protein_coding	1.405451783	2.72E-06	1.73E-05	Up
OAS2	protein_coding	0.74532421	2.74E-06	1.74E-05	Up
CMPK2	protein_coding	0.706677857	2.77E-06	1.75E-05	Up
ANXA8L1	protein_coding	1.197332035	2.92E-06	1.84E-05	Up
DHRS9	protein_coding	1.415294584	2.98E-06	1.87E-05	Up
DIAPH3	protein_coding	0.636506071	3.02E-06	1.89E-05	Up
C15orf48	protein_coding	0.800120609	3.24E-06	2.02E-05	Up
TSPO	protein_coding	0.682846336	3.25E-06	2.02E-05	Up
SLC22A18AS	protein_coding	0.730054833	3.29E-06	2.04E-05	Up
IGSF9	protein_coding	0.920785638	3.39E-06	2.09E-05	Up
HRASLS2	protein_coding	1.148945667	3.44E-06	2.12E-05	Up
POLQ	protein_coding	0.627972265	3.48E-06	2.15E-05	Up
TP73	protein_coding	0.747482153	3.54E-06	2.17E-05	Up
PHLDA2	protein_coding	0.903845459	3.57E-06	2.20E-05	Up
PMAIP1	protein_coding	0.622352489	3.61E-06	2.21E-05	Up
SLCO4A1	protein_coding	0.886800595	3.93E-06	2.39E-05	Up
TJP3	protein_coding	0.785041893	4.13E-06	2.50E-05	Up
KIF14	protein_coding	0.667299148	4.16E-06	2.52E-05	Up
PLLP	protein_coding	0.695983381	4.21E-06	2.54E-05	Up
TNFRSF12A	protein_coding	0.601582729	4.25E-06	2.56E-05	Up
OVOL1	protein_coding	0.935859453	4.28E-06	2.58E-05	Up
PPM1N	protein_coding	0.637337759	4.35E-06	2.62E-05	Up
CORO2A	protein_coding	0.667287748	4.45E-06	2.67E-05	Up
COL7A1	protein_coding	0.991180435	4.60E-06	2.75E-05	Up
C3orf52	protein_coding	0.654248649	4.69E-06	2.80E-05	Up

DNAH2	protein_coding	0.955587622	4.70E-06	2.80E-05	Up
VPS9D1-AS1	long_non_coding	0.642663715	4.76E-06	2.83E-05	Up
BST2	protein_coding	0.66485449	4.79E-06	2.85E-05	Up
GSDMB	protein_coding	0.789374493	4.87E-06	2.89E-05	Up
ADM	protein_coding	0.899051365	4.97E-06	2.94E-05	Up
TRIM16	protein_coding	0.638926061	4.98E-06	2.94E-05	Up
CYP2B7P	pseudogene	1.044174964	4.99E-06	2.95E-05	Up
LYPD3	protein_coding	1.077013562	5.29E-06	3.11E-05	Up
SFTA2	protein_coding	1.089047707	5.30E-06	3.12E-05	Up
ADGRG6	protein_coding	0.725189543	5.36E-06	3.15E-05	Up
PRR11	protein_coding	0.626355388	5.49E-06	3.22E-05	Up
ANXA10	protein_coding	1.262250277	5.57E-06	3.25E-05	Up
PPFIA4	protein_coding	0.934344474	5.69E-06	3.32E-05	Up
CD109	protein_coding	0.764575157	6.09E-06	3.52E-05	Up
PPP1R14D	protein_coding	1.026735407	6.12E-06	3.53E-05	Up
IRX5	protein_coding	0.880224107	6.23E-06	3.59E-05	Up
ADAM8	protein_coding	0.719184856	6.26E-06	3.61E-05	Up
VSTM5	protein_coding	0.683744737	6.58E-06	3.77E-05	Up
SPRR1B	protein_coding	1.673492289	6.58E-06	3.77E-05	Up
F3	protein_coding	0.721368231	6.63E-06	3.79E-05	Up
SOX21	protein_coding	1.304188795	7.23E-06	4.10E-05	Up
MELTF	protein_coding	0.895264464	7.40E-06	4.18E-05	Up
ACY1	protein_coding	0.599812272	7.44E-06	4.20E-05	Up
IL17RE	protein_coding	0.648820536	7.54E-06	4.25E-05	Up
SLC9B2	protein_coding	0.641872948	7.85E-06	4.41E-05	Up
DPCR1	protein_coding	1.611732244	8.19E-06	4.56E-05	Up
RFLNA	protein_coding	0.848858388	8.42E-06	4.68E-05	Up
MUC1	protein_coding	0.868331289	8.71E-06	4.83E-05	Up
SLC52A3	protein_coding	0.668430325	8.75E-06	4.85E-05	Up
LINC01819	long_non_coding	1.504374605	9.28E-06	5.11E-05	Up
APOBEC1	protein_coding	1.295102949	9.42E-06	5.18E-05	Up
RARRES3	protein_coding	0.692781332	9.45E-06	5.19E-05	Up
MUC16	protein_coding	1.502415935	9.51E-06	5.22E-05	Up
FUT3	protein_coding	0.854603781	9.55E-06	5.23E-05	Up
TMEM38A	protein_coding	0.655762526	9.57E-06	5.24E-05	Up
SOX21-AS1	long_non_coding	1.170006094	9.63E-06	5.26E-05	Up
PBK	protein_coding	0.65697086	9.83E-06	5.36E-05	Up
SH3D21	protein_coding	0.749822162	9.85E-06	5.37E-05	Up
CENPF	protein_coding	0.62252157	1.02E-05	5.51E-05	Up
GCKR	protein_coding	0.968189494	1.02E-05	5.53E-05	Up
IHH	protein_coding	1.150250602	1.03E-05	5.57E-05	Up
TNS4	protein_coding	1.173743697	1.05E-05	5.68E-05	Up
HILPDA	protein_coding	0.694160967	1.06E-05	5.70E-05	Up
TSPAN8	protein_coding	0.902368579	1.09E-05	5.84E-05	Up
CBLC	protein_coding	0.717735663	1.13E-05	6.04E-05	Up
TBX15	protein_coding	1.026136555	1.15E-05	6.13E-05	Up
PCSK6	protein_coding	0.619395266	1.19E-05	6.35E-05	Up
IGFBP6	protein_coding	0.713627664	1.21E-05	6.42E-05	Up
KRT5	protein_coding	1.683401416	1.24E-05	6.59E-05	Up
OPLAH	protein_coding	0.588374539	1.27E-05	6.71E-05	Up
ANXA1	protein_coding	0.670745263	1.31E-05	6.89E-05	Up
PPL	protein_coding	0.646914808	1.36E-05	7.14E-05	Up

CDC20	protein_coding	0.727670159	1.39E-05	7.29E-05	Up
TOP2A	protein_coding	0.625864841	1.40E-05	7.33E-05	Up
SIM2	protein_coding	0.831563278	1.41E-05	7.40E-05	Up
MMP1	protein_coding	1.280605284	1.42E-05	7.41E-05	Up
STYK1	protein_coding	0.730551479	1.45E-05	7.54E-05	Up
PLAUR	protein_coding	0.637550761	1.50E-05	7.78E-05	Up
MKRN2OS	protein_coding	0.587528436	1.51E-05	7.81E-05	Up
CYP27C1	protein_coding	0.948959236	1.51E-05	7.85E-05	Up
ADAM28	protein_coding	0.773645928	1.52E-05	7.85E-05	Up
UBE2C	protein_coding	0.749034674	1.55E-05	8.00E-05	Up
ATP8B3	protein_coding	0.678015	1.55E-05	8.00E-05	Up
CLDN23	protein_coding	0.743143963	1.55E-05	8.01E-05	Up
RAPH1	protein_coding	0.58781594	1.57E-05	8.07E-05	Up
FOXC2	protein_coding	0.969914404	1.58E-05	8.14E-05	Up
ELF3	protein_coding	0.664182877	1.61E-05	8.25E-05	Up
TRNP1	protein_coding	0.689299132	1.67E-05	8.56E-05	Up
ABHD11-AS1	pseudogene	0.920788119	1.75E-05	8.92E-05	Up
CRABP2	protein_coding	1.027809593	1.81E-05	9.18E-05	Up
LRFN4	protein_coding	0.652178979	1.84E-05	9.34E-05	Up
PLOD2	protein_coding	0.620695515	1.85E-05	9.37E-05	Up
PLA2G10	protein_coding	0.964152363	1.91E-05	9.64E-05	Up
PODXL	protein_coding	0.601670506	2.01E-05	0.000100603	Up
PSCA	protein_coding	1.442188682	2.12E-05	0.000105302	Up
CLIC3	protein_coding	1.017208477	2.18E-05	0.000108411	Up
TK1	protein_coding	0.659595536	2.30E-05	0.00011331	Up
LINC01232	long_non_coding	0.591138321	2.40E-05	0.000117744	Up
VSIG10L	protein_coding	0.620330903	2.57E-05	0.000125563	Up
STK31	protein_coding	0.875648675	2.58E-05	0.000125914	Up
NEK2	protein_coding	0.654541771	2.67E-05	0.000130018	Up
CARD11	protein_coding	0.614067108	2.78E-05	0.000134651	Up
NIPAL1	protein_coding	0.609079768	2.81E-05	0.000135791	Up
UCA1	long_non_coding	1.259820388	2.85E-05	0.000137205	Up
SEMA3B	protein_coding	0.652047526	2.87E-05	0.000138353	Up
CAPN8	protein_coding	0.95404301	2.99E-05	0.000143343	Up
SULT1C2	protein_coding	1.247731041	3.04E-05	0.000145352	Up
MLPH	protein_coding	0.647983749	3.08E-05	0.000147032	Up
GCNT3	protein_coding	0.886579045	3.09E-05	0.000147669	Up
PNPLA3	protein_coding	0.713386088	3.10E-05	0.000147805	Up
IQGAP3	protein_coding	0.718305543	3.13E-05	0.000149438	Up
EPPK1	protein_coding	0.752555057	3.17E-05	0.000150792	Up
CYP2S1	protein_coding	0.971190067	3.21E-05	0.000152429	Up
C19orf33	protein_coding	0.878919741	3.22E-05	0.000152749	Up
AL590666.2	long_non_coding	0.725846701	3.22E-05	0.000153001	Up
LEMD1	protein_coding	0.966512372	3.23E-05	0.00015337	Up
PRSS21	protein_coding	1.27101205	3.24E-05	0.000153507	Up
PAQR5	protein_coding	0.665763941	3.28E-05	0.000155042	Up
AC007318.1	pseudogene	0.606338597	3.29E-05	0.000155627	Up
WFDC3	protein_coding	0.90904298	3.32E-05	0.000156736	Up
PRDM8	protein_coding	0.606054123	3.45E-05	0.000162183	Up
MELK	protein_coding	0.664195181	3.61E-05	0.000169113	Up
CLDN1	protein_coding	0.687913311	3.70E-05	0.000172502	Up
HLA-K	pseudogene	0.734544492	3.78E-05	0.000175732	Up

CATSPER1	protein_coding	0.752544388	3.82E-05	0.000177382	Up
MX2	protein_coding	0.608531937	3.91E-05	0.000181549	Up
AL121761.2	long_non_coding	1.000851361	3.99E-05	0.000184882	Up
TFCP2L1	protein_coding	0.70082376	4.26E-05	0.000195794	Up
MSLN	protein_coding	1.049266159	4.32E-05	0.000198185	Up
TMC5	protein_coding	0.744340628	4.39E-05	0.000201204	Up
INAVA	protein_coding	0.731139159	4.41E-05	0.000201768	Up
ZNF365	protein_coding	0.703040895	4.55E-05	0.000207597	Up
FRRS1	protein_coding	0.609131272	4.72E-05	0.000214769	Up
SCEL	protein_coding	1.033910735	4.81E-05	0.000217952	Up
SCNN1A	protein_coding	0.782097834	4.84E-05	0.000219257	Up
GABRE	protein_coding	0.773769048	4.91E-05	0.000222267	Up
LINC02432	long_non_coding	0.861847385	4.92E-05	0.000222615	Up
CCNB2	protein_coding	0.606186683	5.03E-05	0.000227006	Up
PCDH7	protein_coding	0.701919408	5.09E-05	0.00022904	Up
CDKN2A	protein_coding	1.140425435	5.16E-05	0.000231898	Up
ACKR4	protein_coding	0.641513771	5.32E-05	0.000238297	Up
ID1	protein_coding	0.642038195	5.49E-05	0.000245293	Up
GJB2	protein_coding	0.802950769	5.65E-05	0.000251489	Up
TGFBI	protein_coding	0.722061529	5.72E-05	0.000253954	Up
ASPM	protein_coding	0.601776174	5.77E-05	0.000256156	Up
IFIT3	protein_coding	0.6000366	5.92E-05	0.000261984	Up
CYP2C18	protein_coding	0.936877031	5.97E-05	0.000263878	Up
FOXM1	protein_coding	0.663224201	6.11E-05	0.000269288	Up
SLC7A11	protein_coding	0.730545728	6.19E-05	0.000272421	Up
EFNA1	protein_coding	0.608549824	6.23E-05	0.000274105	Up
RTKN2	protein_coding	0.667897234	6.31E-05	0.00027744	Up
SH3RF2	protein_coding	0.667150323	6.33E-05	0.00027802	Up
GJC2	protein_coding	0.833712337	6.54E-05	0.000286345	Up
PKMYT1	protein_coding	0.632143745	6.55E-05	0.000286491	Up
RASAL1	protein_coding	0.690544515	6.57E-05	0.000287121	Up
MMEL1	protein_coding	0.671339186	6.69E-05	0.000291727	Up
MYOM3	protein_coding	0.716202488	7.03E-05	0.000304666	Up
SLC7A5	protein_coding	0.661830935	7.36E-05	0.000317417	Up
OTX1	protein_coding	0.650490419	7.62E-05	0.00032724	Up
SPINK5	protein_coding	0.92136475	7.70E-05	0.000329881	Up
ITPKA	protein_coding	0.870756782	7.81E-05	0.000333826	Up
FSIP2	protein_coding	0.870252968	7.82E-05	0.000334267	Up
AC009065.2	long_non_coding	0.729350981	7.88E-05	0.00033651	Up
MUC4	protein_coding	1.06077991	8.04E-05	0.000342204	Up
SULT1E1	protein_coding	1.1944347	8.06E-05	0.000342929	Up
ADAP1	protein_coding	0.772662462	8.44E-05	0.000357101	Up
GDPD2	protein_coding	0.895116486	8.50E-05	0.000359147	Up
AREG	protein_coding	0.83538719	8.71E-05	0.000366936	Up
BLACAT1	long_non_coding	0.823784563	8.78E-05	0.000369282	Up
PRSS12	protein_coding	0.608155265	9.14E-05	0.000382431	Up
CEACAM1	protein_coding	0.622759235	9.45E-05	0.000393857	Up
VSIG2	protein_coding	0.863195339	9.83E-05	0.00040686	Up
NMU	protein_coding	0.980945348	9.87E-05	0.000408688	Up
UPK3B	protein_coding	1.107972808	9.97E-05	0.00041202	Up
BAIAP2L2	protein_coding	0.772960746	0.000100081	0.000413425	Up
BX470102.1	long_non_coding	0.88239391	0.000100421	0.000414717	Up

IFIT1	protein_coding	0.718200993	0.000103356	0.000424874	Up
STX19	protein_coding	0.686731201	0.000107201	0.000438898	Up
MFSD4A	protein_coding	0.748963554	0.000107578	0.000440322	Up
CLDN4	protein_coding	0.638791167	0.000108342	0.000442732	Up
CA9	protein_coding	1.153529069	0.000108476	0.000442804	Up
CRYBG2	protein_coding	0.763161179	0.000109053	0.00044432	Up
ARL4D	protein_coding	0.603403992	0.000110774	0.000450367	Up
S100A6	protein_coding	0.60850017	0.000114095	0.000462507	Up
MIR222HG	long_non_coding	0.655965131	0.000116953	0.000472953	Up
LIPM	protein_coding	0.710192733	0.000118032	0.000476049	Up
GRHL1	protein_coding	0.598880556	0.000119658	0.000481457	Up
MIR210HG	long_non_coding	0.810807236	0.000122396	0.00049143	Up
NQO1	protein_coding	0.627973678	0.000123477	0.000494984	Up
FOXD2	protein_coding	0.624016418	0.000126673	0.00050503	Up
STRIP2	protein_coding	0.636322615	0.000130367	0.000517142	Up
SLC16A4	protein_coding	0.611977166	0.000133315	0.00052677	Up
C6orf223	protein_coding	0.826739628	0.000134103	0.000529056	Up
TFAP2C	protein_coding	0.8819257	0.000141092	0.00055218	Up
PVT1	long_non_coding	0.626571448	0.000147858	0.000574806	Up
ABCC3	protein_coding	0.610186504	0.000147966	0.000575077	Up
SP6	protein_coding	0.610723373	0.000149511	0.000580488	Up
EVPL	protein_coding	0.725718148	0.000152839	0.000592952	Up
CLDN18	protein_coding	1.327450131	0.000153106	0.000593554	Up
XKR9	protein_coding	0.704688031	0.00015332	0.000594059	Up
MUC17	protein_coding	1.392991183	0.000159911	0.00061645	Up
HIST2H2BE	protein_coding	0.595232591	0.00016245	0.000625042	Up
SEMA7A	protein_coding	0.706968506	0.00016669	0.000640144	Up
WNT5A	protein_coding	0.595277281	0.000167524	0.000642696	Up
LINC02188	long_non_coding	0.984038555	0.000167621	0.000642906	Up
EPS8L1	protein_coding	0.761270687	0.000175765	0.000670923	Up
MAPK15	protein_coding	0.774917009	0.000179333	0.000682654	Up
FAM83B	protein_coding	0.63372087	0.0001862	0.000704942	Up
GPT	protein_coding	0.790242274	0.000187951	0.000710291	Up
MX1	protein_coding	0.625749605	0.000189121	0.000714182	Up
POF1B	protein_coding	0.652692897	0.000190073	0.000716529	Up
LINC00482	long_non_coding	0.733238368	0.000197494	0.000742659	Up
AMN	protein_coding	0.852559466	0.000198764	0.000745953	Up
ZBED2	protein_coding	1.039889839	0.000209632	0.000780947	Up
CALB2	protein_coding	0.906577813	0.000216458	0.000803811	Up
GRHL2	protein_coding	0.589958958	0.000217022	0.000805318	Up
COX6B2	protein_coding	0.960039944	0.000221936	0.000821344	Up
AC136475.3	long_non_coding	0.937669627	0.000227815	0.000840436	Up
SFN	protein_coding	0.782772745	0.000229686	0.000846718	Up
CEACAM5	protein_coding	1.21572588	0.000231233	0.000851516	Up
CHAC1	protein_coding	0.603684681	0.000232696	0.00085615	Up
ANK1	protein_coding	0.686604298	0.000239457	0.000878896	Up
VSIG1	protein_coding	1.132993208	0.000241373	0.000885502	Up
KLK10	protein_coding	0.934149587	0.000244622	0.000895687	Up
RPL39L	protein_coding	0.599124115	0.000250278	0.000913752	Up
CXCL14	protein_coding	0.789246373	0.000255365	0.000929417	Up
EPHB6	protein_coding	0.654925492	0.00026329	0.000955055	Up
ANXA8	protein_coding	1.047885323	0.000268551	0.000971354	Up

SLC22A3	protein_coding	0.628225733	0.000270983	0.000978283	Up
AC108134.1	long_non_coding	0.637960221	0.000277147	0.000997215	Up
KRT80	protein_coding	0.638047191	0.000278792	0.001002424	Up
AC009065.5	long_non_coding	0.672324189	0.000279078	0.001003213	Up
POU5F1	protein_coding	0.818293057	0.000279477	0.001004136	Up
IGSF9B	protein_coding	0.626357181	0.000284929	0.001021343	Up
SIGLEC15	protein_coding	0.87189292	0.000288164	0.001029338	Up
CTSE	protein_coding	0.897141139	0.000299664	0.001064857	Up
HHIP	protein_coding	0.755480586	0.000319593	0.001123834	Up
ACER2	protein_coding	0.687822974	0.000327346	0.001147376	Up
PERM1	protein_coding	0.688906381	0.000329369	0.001152866	Up
SNAI2	protein_coding	0.628573146	0.000335513	0.00117167	Up
PCDHGB2	protein_coding	0.764255222	0.000348271	0.001210097	Up
AC128688.2	long_non_coding	0.588356009	0.000363283	0.001254504	Up
PGGHG	protein_coding	0.728878175	0.000369368	0.001273201	Up
SLC23A3	protein_coding	0.616903246	0.000376625	0.001295273	Up
TMEM45B	protein_coding	0.631469021	0.000386706	0.001324132	Up
TRIM15	protein_coding	0.810761181	0.000407896	0.001388346	Up
WNT11	protein_coding	1.007097027	0.000423675	0.001434974	Up
ADGRF1	protein_coding	0.792008657	0.000427373	0.001446206	Up
ADGRF4	protein_coding	0.647835057	0.000434063	0.001466557	Up
ISG15	protein_coding	0.733253544	0.000446391	0.001503858	Up
SNCG	protein_coding	0.833019433	0.000457486	0.001536631	Up
SLC6A14	protein_coding	0.930018993	0.000459907	0.001543907	Up
NECTIN4	protein_coding	0.707258414	0.000488314	0.001629539	Up
FER1L6	protein_coding	1.15693722	0.000497497	0.001656903	Up
LYNX1	protein_coding	0.649964677	0.000519154	0.001717723	Up
ALDH3A1	protein_coding	0.944767914	0.000544205	0.001789638	Up
SLC9A3	protein_coding	0.927071919	0.000548334	0.001799756	Up
SLC44A4	protein_coding	0.585821405	0.000565595	0.001850408	Up
AL390719.1	pseudogene	0.595256617	0.000622421	0.002014175	Up
PRSS8	protein_coding	0.626476766	0.000629984	0.002037346	Up
CHST6	protein_coding	0.726203309	0.000645091	0.002079114	Up
BARX2	protein_coding	0.616565724	0.000645476	0.002079913	Up
B3GNT7	protein_coding	0.648699304	0.000649484	0.00209051	Up
LRRN1	protein_coding	0.629045866	0.000656068	0.002108225	Up
CYP2W1	protein_coding	0.881216636	0.000669882	0.002148066	Up
SLPI	protein_coding	0.668425483	0.000673387	0.002157483	Up
PSORS1C1	protein_coding	0.656086972	0.000697869	0.002226053	Up
COL11A1	protein_coding	0.961203533	0.000698619	0.002227507	Up
AC103702.2	long_non_coding	0.991152185	0.000710946	0.002261587	Up
LGR6	protein_coding	0.828591964	0.000725739	0.002304296	Up
HSD17B2	protein_coding	0.696091292	0.000771929	0.002428601	Up
TNNC2	protein_coding	0.730832724	0.000773314	0.002432115	Up
SYTL5	protein_coding	0.830076226	0.000776217	0.002439563	Up
CEACAM6	protein_coding	0.782370947	0.000784386	0.002460652	Up
EPS8L3	protein_coding	0.72521668	0.000798826	0.002499748	Up
TRIM31	protein_coding	0.815001469	0.000809702	0.002527011	Up
ATG9B	protein_coding	0.706605457	0.000852867	0.002645404	Up
G0S2	protein_coding	0.660908789	0.000902581	0.002779724	Up
UPK1B	protein_coding	1.165292216	0.000914217	0.002808151	Up
SLC17A9	protein_coding	0.588033294	0.000915156	0.002810466	Up

SUGCT	protein_coding	0.610454034	0.000928434	0.00284491	Up
HCAR1	protein_coding	0.806095526	0.000932035	0.002853641	Up
PI3	protein_coding	0.901175335	0.000938165	0.002868937	Up
HYAL1	protein_coding	0.62256389	0.000947944	0.002896507	Up
ADAMTS12	protein_coding	0.605580376	0.000952309	0.002907503	Up
ALG1L	protein_coding	0.729528103	0.000962959	0.002938836	Up
GPR35	protein_coding	0.609081709	0.000974448	0.002969718	Up
SLC6A20	protein_coding	0.66307306	0.000985719	0.00299865	Up
SLC38A5	protein_coding	0.753622622	0.001011467	0.003069594	Up
PAX8	protein_coding	0.630813141	0.001053316	0.003178166	Up
TNFSF11	protein_coding	0.623624306	0.001071877	0.003223916	Up
MT1L	pseudogene	0.585353776	0.001111156	0.003326235	Up
CSTA	protein_coding	0.669918106	0.00118716	0.003525936	Up
HSPA1A	protein_coding	0.627287102	0.001195852	0.003550361	Up
AC019117.1	long_non_coding	0.698373336	0.001209272	0.003584595	Up
EDAR	protein_coding	0.712195954	0.001244226	0.003673855	Up
FAM83E	protein_coding	0.612815518	0.001294136	0.00379966	Up
DUOXA1	protein_coding	0.724510249	0.001309774	0.003837536	Up
CYP4F11	protein_coding	0.680631832	0.001333632	0.003899158	Up
INHBA	protein_coding	0.621324066	0.001347819	0.003936089	Up
PTHLH	protein_coding	0.639586404	0.001383444	0.004024638	Up
IFI6	protein_coding	0.589993207	0.001389297	0.00403934	Up
AC021218.1	long_non_coding	0.830724328	0.001435381	0.004162961	Up
FEZF1-AS1	long_non_coding	1.039741354	0.001446413	0.004191752	Up
HOXA13	protein_coding	1.100739283	0.001469147	0.004245478	Up
SULT1B1	protein_coding	0.869161439	0.001475599	0.004263309	Up
FN1	protein_coding	0.627650018	0.001524452	0.004385257	Up
DQX1	protein_coding	0.892175719	0.001541695	0.004432337	Up
COL12A1	protein_coding	0.628770605	0.001559937	0.004477995	Up
GRIN2D	protein_coding	0.643736977	0.001569767	0.004501957	Up
S100P	protein_coding	0.842581238	0.00160751	0.004601506	Up
SIX1	protein_coding	0.740405624	0.001648569	0.004704838	Up
ULBP2	protein_coding	0.613563774	0.001680379	0.004780341	Up
HTR1D	protein_coding	0.607419755	0.001696711	0.004816871	Up
RHOV	protein_coding	0.686828707	0.001782268	0.005024983	Up
SERPINB2	protein_coding	0.791887286	0.001841289	0.005173129	Up
C4BPB	protein_coding	0.710982533	0.001866776	0.005231176	Up
MMP11	protein_coding	0.730849353	0.001879529	0.005261087	Up
Z98257.1	long_non_coding	0.929198971	0.001984176	0.005518386	Up
ALDH3B2	protein_coding	0.808800682	0.002018807	0.005605458	Up
AL365181.3	long_non_coding	0.63987852	0.002019993	0.005607727	Up
CYP3A5	protein_coding	0.608088956	0.002427328	0.006600889	Up
MACROD2	protein_coding	0.600679093	0.002583134	0.0069746	Up
FIBCD1	protein_coding	0.897259904	0.002730997	0.007334691	Up
AL355312.3	long_non_coding	0.663472488	0.002770211	0.007421622	Up
SUCNR1	protein_coding	0.764535763	0.003128302	0.008247046	Up
HP	protein_coding	0.832565464	0.003150226	0.008296197	Up
KANK4	protein_coding	0.653598995	0.003407212	0.008885095	Up
LINC02086	long_non_coding	0.973023842	0.003582155	0.009277536	Up
AGMO	protein_coding	0.709811822	0.003608317	0.009337805	Up
NXF3	protein_coding	0.896064792	0.003757275	0.00967331	Up
ABCA12	protein_coding	0.633463327	0.003810048	0.009787593	Up

WNT10A	protein_coding	0.654935411	0.003977607	0.010156147	Up
PADI1	protein_coding	0.954799697	0.004087203	0.010414957	Up
WFDC2	protein_coding	0.615532427	0.004129178	0.010498855	Up
IFI44L	protein_coding	0.605998401	0.004177887	0.010605082	Up
SYT12	protein_coding	0.605933133	0.004275435	0.01083096	Up
GRP	protein_coding	0.613174515	0.004311048	0.01091025	Up
CP	protein_coding	0.739772225	0.004796121	0.011964289	Up
CNTNAP2	protein_coding	0.716319234	0.005220237	0.012884621	Up
COL10A1	protein_coding	0.646676723	0.005466914	0.013408429	Up
PCSK9	protein_coding	0.586219812	0.005660058	0.013817731	Up
SRMS	protein_coding	0.637048416	0.005890547	0.014292699	Up
MUC5AC	protein_coding	0.956896798	0.005928622	0.014364414	Up
PTGS2	protein_coding	0.672190559	0.006100276	0.014735618	Up
PLPP4	protein_coding	0.592213813	0.006167045	0.01487775	Up
CARNS1	protein_coding	0.596776302	0.00616826	0.01487775	Up
CDHR2	protein_coding	0.663184203	0.006184013	0.014909415	Up
LRRC66	protein_coding	0.614756086	0.006334667	0.015226678	Up
ALDH1L1	protein_coding	0.637158599	0.006599165	0.0157502	Up
LRRC15	protein_coding	0.729712245	0.006640662	0.015836788	Up
AGR3	protein_coding	0.635699761	0.006801023	0.016173475	Up
SHH	protein_coding	0.60257703	0.006804673	0.01617962	Up
SLC24A2	protein_coding	0.611637611	0.006921907	0.016409525	Up
CYP2C9	protein_coding	0.738188009	0.007164275	0.016894146	Up
DUOXA2	protein_coding	0.799781856	0.007356578	0.017256689	Up
ST6GALNAC1	protein_coding	0.595588363	0.008122912	0.018790037	Up
PHGR1	protein_coding	0.908240853	0.008468703	0.019485978	Up
C16orf74	protein_coding	0.613124792	0.009138267	0.020852726	Up
HOXA10	protein_coding	0.698906215	0.009501349	0.021587123	Up
TFF1	protein_coding	0.828383525	0.010756772	0.024061242	Up
GABRP	protein_coding	0.778759636	0.011532096	0.025558211	Up
NRG3	protein_coding	0.591106175	0.01207941	0.026588718	Up
SLC14A1	protein_coding	0.690257043	0.012601554	0.027582028	Up
UGT2B7	protein_coding	0.681671897	0.012848673	0.028017861	Up
AL365181.2	long_non_coding	0.627379884	0.013895953	0.029931716	Up
VSTM2L	protein_coding	0.607750014	0.014093577	0.030284329	Up
IGFL2	protein_coding	0.631828331	0.014480645	0.030993196	Up
BIRC7	protein_coding	0.632015907	0.015458263	0.032817184	Up
KBTBD12	protein_coding	0.616146458	0.015784553	0.033402421	Up
VGLL1	protein_coding	0.65664495	0.016159663	0.034082157	Up
CES3	protein_coding	0.593543579	0.016192954	0.034136878	Up
AC018629.1	TEC	0.60634267	0.0164587	0.034621439	Up
TFF2	protein_coding	0.794987373	0.018054184	0.037485999	Up
TNNI2	protein_coding	0.595375565	0.019823153	0.040689631	Up
CAPN9	protein_coding	0.663676283	0.020328898	0.041553089	Up
NLRP2	protein_coding	0.696324546	0.021417475	0.043462096	Up
SYT8	protein_coding	0.661111397	0.021533307	0.043644587	Up
DUOX2	protein_coding	0.631809613	0.022784802	0.045905342	Up
FGG	protein_coding	0.770649436	0.025044779	0.049823648	Up
BTNL8	protein_coding	0.687359513	0.026516143	0.052298311	Up
SLC26A9	protein_coding	0.636149868	0.029653141	0.057411115	Up
LINC00675	protein_coding	0.644823438	0.034522962	0.065298479	Up
CXCL17	protein_coding	0.586952899	0.038701693	0.071913537	Up

CST6	protein_coding	0.591884002	0.040872628	0.075346032	Up
PIK3C2G	protein_coding	0.586165047	0.044472809	0.080979463	Up
RUNDC3A	protein_coding	-3.044886886	1.75E-26	2.66E-22	Down
NCAM1	protein_coding	-2.39847484	4.00E-26	3.03E-22	Down
KCNB1	protein_coding	-2.68519562	1.68E-25	8.51E-22	Down
TCEAL2	protein_coding	-2.916010116	2.39E-25	9.07E-22	Down
TMEM179	protein_coding	-3.443481094	1.18E-24	2.98E-21	Down
TMEM63C	protein_coding	-3.15110658	1.55E-24	3.36E-21	Down
SYP	protein_coding	-2.574666846	4.08E-24	7.74E-21	Down
SNAP25	protein_coding	-2.429839826	7.40E-24	1.25E-20	Down
NOVA1	protein_coding	-2.076313017	8.37E-24	1.27E-20	Down
RIMBP2	protein_coding	-2.877835817	9.21E-24	1.27E-20	Down
UNC13A	protein_coding	-2.547167492	2.21E-23	2.79E-20	Down
SPTBN4	protein_coding	-2.344291893	2.71E-23	3.16E-20	Down
MSI1	protein_coding	-2.045196667	5.43E-23	5.89E-20	Down
IQSEC3	protein_coding	-2.172976886	6.22E-23	6.29E-20	Down
TSPAN7	protein_coding	-1.890998765	1.05E-22	9.94E-20	Down
FAM222A	protein_coding	-2.085726436	1.33E-22	1.19E-19	Down
CACNA1A	protein_coding	-2.37753985	3.56E-22	2.93E-19	Down
CACNA2D2	protein_coding	-2.265179517	3.69E-22	2.93E-19	Down
UNC79	protein_coding	-2.43095013	3.86E-22	2.93E-19	Down
SYT4	protein_coding	-3.114092019	5.00E-22	3.49E-19	Down
ATP2B1-AS1	long_non_coding	-1.453094533	5.06E-22	3.49E-19	Down
ATP1A2	protein_coding	-2.313600445	8.27E-22	5.46E-19	Down
REEP2	protein_coding	-2.138299538	8.69E-22	5.49E-19	Down
PGM5	protein_coding	-1.851311172	1.58E-21	9.58E-19	Down
VWA5B2	protein_coding	-2.902067817	2.04E-21	1.19E-18	Down
CXorf57	protein_coding	-1.933928104	4.05E-21	2.27E-18	Down
GPX3	protein_coding	-1.930511876	5.69E-21	3.00E-18	Down
SCN3B	protein_coding	-1.956596411	5.74E-21	3.00E-18	Down
APLP1	protein_coding	-2.474806033	8.92E-21	4.46E-18	Down
SNAP91	protein_coding	-2.246069414	9.10E-21	4.46E-18	Down
MIR600HG	long_non_coding	-1.351520905	1.37E-20	6.31E-18	Down
BSN	protein_coding	-2.044646545	2.14E-20	9.45E-18	Down
CACNB2	protein_coding	-1.620827782	2.18E-20	9.45E-18	Down
SESN1	protein_coding	-0.886884994	3.45E-20	1.46E-17	Down
ANK2	protein_coding	-1.670630061	4.41E-20	1.81E-17	Down
KL	protein_coding	-2.061876522	4.77E-20	1.90E-17	Down
AP3B2	protein_coding	-2.114406317	1.06E-19	4.13E-17	Down
RTN1	protein_coding	-2.077647281	1.16E-19	4.40E-17	Down
KIF5C	protein_coding	-2.021527871	1.19E-19	4.40E-17	Down
SFRP1	protein_coding	-2.129729287	1.29E-19	4.66E-17	Down
CELF3	protein_coding	-2.831218407	1.39E-19	4.91E-17	Down
NAP1L5	protein_coding	-1.29207561	1.49E-19	5.14E-17	Down
INA	protein_coding	-2.483016115	1.57E-19	5.26E-17	Down
NAP1L2	protein_coding	-1.6872544	2.26E-19	7.18E-17	Down
AFF3	protein_coding	-1.789768328	2.27E-19	7.18E-17	Down
PPP1R1A	protein_coding	-2.704675405	2.85E-19	8.82E-17	Down
CHRDL2	protein_coding	-2.281202715	3.60E-19	1.09E-16	Down
CLGN	protein_coding	-2.295851166	3.74E-19	1.11E-16	Down
CNIH2	protein_coding	-2.258468783	4.63E-19	1.35E-16	Down
DPP6	protein_coding	-2.538369962	4.87E-19	1.40E-16	Down

FCMR	protein_coding	-1.947157367	5.04E-19	1.40E-16	Down
PACSIN1	protein_coding	-1.881772631	5.10E-19	1.40E-16	Down
GRIK5	protein_coding	-1.92681699	5.16E-19	1.40E-16	Down
DUSP26	protein_coding	-2.364171766	5.92E-19	1.58E-16	Down
UNC80	protein_coding	-2.642567073	6.16E-19	1.61E-16	Down
ATOH8	protein_coding	-1.447150343	6.24E-19	1.61E-16	Down
PDZD4	protein_coding	-1.681165337	6.69E-19	1.68E-16	Down
MPP2	protein_coding	-1.764884318	6.84E-19	1.68E-16	Down
MTMR7	protein_coding	-1.707729116	6.87E-19	1.68E-16	Down
SEZ6L	protein_coding	-2.69484306	7.19E-19	1.73E-16	Down
CDO1	protein_coding	-1.614803117	8.24E-19	1.95E-16	Down
ZNF540	protein_coding	-1.247669798	8.45E-19	1.96E-16	Down
GPRASP1	protein_coding	-1.510429421	8.52E-19	1.96E-16	Down
SVOP	protein_coding	-2.734838256	8.74E-19	1.98E-16	Down
PHACTR1	protein_coding	-1.206252125	1.02E-18	2.27E-16	Down
CXXC4	protein_coding	-1.835213568	1.13E-18	2.48E-16	Down
CDK5R2	protein_coding	-2.475362455	1.19E-18	2.58E-16	Down
NANOS1	protein_coding	-1.619946805	1.27E-18	2.71E-16	Down
GPR162	protein_coding	-1.434973475	1.31E-18	2.76E-16	Down
FAM184A	protein_coding	-1.246547012	2.22E-18	4.61E-16	Down
AC010478.1	long_non_coding	-1.867383443	2.25E-18	4.61E-16	Down
OTUD7A	protein_coding	-1.009121935	2.51E-18	5.07E-16	Down
UCHL1	protein_coding	-1.60047608	2.57E-18	5.11E-16	Down
ADGRG5	protein_coding	-1.732541048	2.59E-18	5.11E-16	Down
SSTR2	protein_coding	-1.907915034	3.01E-18	5.85E-16	Down
EML6	protein_coding	-1.281776486	3.39E-18	6.50E-16	Down
SNPH	protein_coding	-1.309773558	3.84E-18	7.29E-16	Down
SCG2	protein_coding	-2.323468673	4.79E-18	8.95E-16	Down
DIRAS1	protein_coding	-1.895523693	4.84E-18	8.95E-16	Down
PAK3	protein_coding	-2.096930284	4.97E-18	9.10E-16	Down
INSM1	protein_coding	-2.699963175	5.81E-18	1.05E-15	Down
FMN2	protein_coding	-2.171312338	6.32E-18	1.13E-15	Down
KIF5A	protein_coding	-1.80120371	7.25E-18	1.28E-15	Down
CAMK2B	protein_coding	-2.506806531	7.97E-18	1.39E-15	Down
GNAO1	protein_coding	-1.675613958	8.44E-18	1.46E-15	Down
SOCS2-AS1	long_non_coding	-1.124276964	8.64E-18	1.47E-15	Down
GNG2	protein_coding	-1.378213501	8.69E-18	1.47E-15	Down
OGDHL	protein_coding	-2.167623589	9.67E-18	1.61E-15	Down
CPLX2	protein_coding	-2.964732173	9.80E-18	1.62E-15	Down
SPTB	protein_coding	-1.664775502	1.13E-17	1.84E-15	Down
GKAP1	protein_coding	-1.018124184	1.20E-17	1.93E-15	Down
GIMAP7	protein_coding	-1.282492344	1.21E-17	1.93E-15	Down
IL24	protein_coding	-1.871088821	1.28E-17	2.02E-15	Down
BEX1	protein_coding	-2.484264841	1.30E-17	2.03E-15	Down
SOCS2	protein_coding	-0.939190955	1.34E-17	2.07E-15	Down
SERPINF2	protein_coding	-1.517001141	1.69E-17	2.59E-15	Down
PSMG3-AS1	long_non_coding	-1.06552148	1.75E-17	2.65E-15	Down
NFASC	protein_coding	-1.577427636	1.89E-17	2.84E-15	Down
ARNT2	protein_coding	-1.214436972	1.98E-17	2.94E-15	Down
PTPRN	protein_coding	-2.528137076	2.17E-17	3.20E-15	Down
ACACB	protein_coding	-1.056286539	2.81E-17	4.10E-15	Down
RIC3	protein_coding	-1.795424516	3.12E-17	4.52E-15	Down

GNG7	protein_coding	-1.274568311	3.26E-17	4.66E-15	Down
CALY	protein_coding	-2.659054879	3.31E-17	4.70E-15	Down
NOVA2	protein_coding	-1.177317191	3.54E-17	4.97E-15	Down
PRLR	protein_coding	-1.855921362	3.86E-17	5.37E-15	Down
RND2	protein_coding	-1.456040872	4.46E-17	6.15E-15	Down
TRAF3IP2-AS1	long_non_coding	-0.727217645	4.69E-17	6.38E-15	Down
44440	protein_coding	-1.379874409	4.71E-17	6.38E-15	Down
ZFP3	protein_coding	-0.770432221	4.77E-17	6.41E-15	Down
EPM2A	protein_coding	-0.788741262	4.97E-17	6.62E-15	Down
SGSM1	protein_coding	-1.680997681	5.17E-17	6.83E-15	Down
PPP1R16B	protein_coding	-1.312835522	5.33E-17	6.97E-15	Down
PCSK1	protein_coding	-2.306545806	5.42E-17	7.03E-15	Down
STAT4	protein_coding	-1.131697675	6.73E-17	8.66E-15	Down
RASGRP2	protein_coding	-1.677268945	6.80E-17	8.68E-15	Down
NECAB2	protein_coding	-2.019784141	8.21E-17	1.04E-14	Down
PTX3	protein_coding	-2.173059119	9.10E-17	1.14E-14	Down
MIR7-3HG	long_non_coding	-2.689223416	1.02E-16	1.27E-14	Down
GALNT8	protein_coding	-1.846486443	1.05E-16	1.29E-14	Down
NUAK2	protein_coding	-0.926890655	1.20E-16	1.46E-14	Down
SLC4A8	protein_coding	-1.669317929	1.27E-16	1.53E-14	Down
ICA1L	protein_coding	-1.189932088	1.31E-16	1.57E-14	Down
CD79B	protein_coding	-2.13791188	1.34E-16	1.57E-14	Down
ARHGEF26	protein_coding	-1.278094318	1.34E-16	1.57E-14	Down
RIPOR2	protein_coding	-1.636721707	1.35E-16	1.58E-14	Down
CCR7	protein_coding	-2.001957058	1.54E-16	1.78E-14	Down
RFXAP	protein_coding	-0.661112579	1.66E-16	1.91E-14	Down
ELMO1	protein_coding	-1.153566481	1.76E-16	2.01E-14	Down
LINC00926	long_non_coding	-1.988107319	2.09E-16	2.33E-14	Down
CYFIP2	protein_coding	-1.074031391	2.09E-16	2.33E-14	Down
TMEM170B	protein_coding	-0.897773888	2.80E-16	3.08E-14	Down
PRKCB	protein_coding	-1.675004734	2.82E-16	3.08E-14	Down
MMRN1	protein_coding	-1.735936559	3.14E-16	3.41E-14	Down
ZNF667	protein_coding	-1.101535299	3.38E-16	3.64E-14	Down
BEX4	protein_coding	-1.150460135	3.45E-16	3.69E-14	Down
CD22	protein_coding	-2.054901421	3.76E-16	3.99E-14	Down
RFX6	protein_coding	-2.554749726	4.08E-16	4.27E-14	Down
DLG2	protein_coding	-1.308785305	4.22E-16	4.38E-14	Down
SLCO5A1	protein_coding	-1.340151367	4.35E-16	4.46E-14	Down
RAPGEF4	protein_coding	-1.109473065	4.55E-16	4.60E-14	Down
ST18	protein_coding	-2.088074136	4.65E-16	4.68E-14	Down
SCML4	protein_coding	-1.397666232	4.75E-16	4.71E-14	Down
AC233723.2	TEC	-1.1080272	4.75E-16	4.71E-14	Down
ZNF667-AS1	long_non_coding	-1.053353035	5.19E-16	5.09E-14	Down
GNAZ	protein_coding	-1.579135181	5.20E-16	5.09E-14	Down
AP001972.5	TEC	-0.883113867	5.23E-16	5.09E-14	Down
VAMP2	protein_coding	-0.852708951	5.56E-16	5.38E-14	Down
NPR1	protein_coding	-1.014948961	5.66E-16	5.44E-14	Down
RTL5	protein_coding	-1.409264998	5.97E-16	5.70E-14	Down
PPM1K	protein_coding	-0.942121339	6.29E-16	5.97E-14	Down
MAPK8IP1	protein_coding	-1.422384609	6.41E-16	6.05E-14	Down
ZNF671	protein_coding	-0.853650112	6.84E-16	6.41E-14	Down
MTURN	protein_coding	-0.881954684	6.94E-16	6.45E-14	Down

FAM107A	protein_coding	-1.334475744	7.38E-16	6.79E-14	Down
MAPT	protein_coding	-1.79550551	7.76E-16	7.09E-14	Down
CARMIL2	protein_coding	-1.501253432	7.83E-16	7.12E-14	Down
SLC38A4	protein_coding	-2.31078973	8.16E-16	7.37E-14	Down
GALNT16	protein_coding	-1.445813902	8.79E-16	7.90E-14	Down
LRRC10B	protein_coding	-1.992899711	8.93E-16	7.97E-14	Down
SELL	protein_coding	-1.694603486	9.03E-16	8.01E-14	Down
SLC26A11	protein_coding	-0.99931733	9.99E-16	8.81E-14	Down
TNFRSF13C	protein_coding	-2.271367189	1.12E-15	9.84E-14	Down
LHFPL4	protein_coding	-2.361621051	1.22E-15	1.07E-13	Down
ATP1A3	protein_coding	-1.684033321	1.28E-15	1.11E-13	Down
ARSG	protein_coding	-0.688666376	1.31E-15	1.13E-13	Down
SCG3	protein_coding	-2.511408411	1.42E-15	1.21E-13	Down
SLC29A4	protein_coding	-1.890853853	1.44E-15	1.22E-13	Down
FAM69B	protein_coding	-1.232516978	1.50E-15	1.27E-13	Down
CBX7	protein_coding	-0.779390675	1.57E-15	1.32E-13	Down
GPRIN3	protein_coding	-1.002785636	1.63E-15	1.36E-13	Down
ERO1B	protein_coding	-1.915480669	1.75E-15	1.45E-13	Down
AL357060.2	long_non_coding	-0.986083036	1.89E-15	1.56E-13	Down
ZNF582	protein_coding	-0.903881331	1.90E-15	1.56E-13	Down
NKX2-2	protein_coding	-2.593212866	2.00E-15	1.62E-13	Down
CDIP1	protein_coding	-0.788062225	2.03E-15	1.63E-13	Down
SCG5	protein_coding	-2.138200116	2.44E-15	1.96E-13	Down
GHR	protein_coding	-1.309065543	2.56E-15	2.04E-13	Down
CHRDL1	protein_coding	-1.936599982	2.73E-15	2.17E-13	Down
DTNA	protein_coding	-1.294820501	2.77E-15	2.19E-13	Down
GLCCI1	protein_coding	-0.786556669	2.98E-15	2.35E-13	Down
MVB12B	protein_coding	-0.851271735	3.06E-15	2.39E-13	Down
ADCY1	protein_coding	-1.740711172	3.14E-15	2.45E-13	Down
EFR3B	protein_coding	-1.368587529	3.19E-15	2.45E-13	Down
FXYD6	protein_coding	-1.230599985	3.20E-15	2.45E-13	Down
TMOD1	protein_coding	-1.735616563	3.32E-15	2.53E-13	Down
KCNJ3	protein_coding	-2.096558787	3.35E-15	2.55E-13	Down
TMEM178B	protein_coding	-1.691349328	3.42E-15	2.58E-13	Down
ABCC8	protein_coding	-2.535016551	3.45E-15	2.59E-13	Down
GRIA3	protein_coding	-1.849872261	3.46E-15	2.59E-13	Down
AMPH	protein_coding	-1.490804669	3.85E-15	2.86E-13	Down
IGF1	protein_coding	-1.964481693	4.24E-15	3.14E-13	Down
MAPK10	protein_coding	-1.257007209	4.44E-15	3.25E-13	Down
ISL1	protein_coding	-1.717339801	4.46E-15	3.26E-13	Down
SORCS1	protein_coding	-1.719032656	5.00E-15	3.63E-13	Down
FAM212B	protein_coding	-0.799320934	5.14E-15	3.72E-13	Down
TSPAN33	protein_coding	-1.169343154	5.25E-15	3.78E-13	Down
RASD1	protein_coding	-1.66992681	5.27E-15	3.78E-13	Down
ARX	protein_coding	-2.424723722	5.34E-15	3.80E-13	Down
RAB3C	protein_coding	-1.743470816	5.38E-15	3.82E-13	Down
ELAVL4	protein_coding	-1.930375794	5.68E-15	4.01E-13	Down
NEURL1	protein_coding	-1.931894486	5.76E-15	4.05E-13	Down
SRSF12	protein_coding	-1.120576721	6.30E-15	4.41E-13	Down
SMAP2	protein_coding	-0.913199117	7.22E-15	4.98E-13	Down
BCL11A	protein_coding	-1.326160525	7.73E-15	5.31E-13	Down
GIMAP1	protein_coding	-1.012168328	8.06E-15	5.51E-13	Down

ACAP1	protein_coding	-1.231653297	9.38E-15	6.38E-13	Down
ANKRD44	protein_coding	-0.937136474	9.77E-15	6.62E-13	Down
P2RY8	protein_coding	-1.283621837	1.00E-14	6.77E-13	Down
ITK	protein_coding	-1.661165003	1.17E-14	7.79E-13	Down
MTUS2	protein_coding	-1.851585117	1.17E-14	7.79E-13	Down
LINC00909	long_non_coding	-0.645526123	1.18E-14	7.79E-13	Down
CD99L2	protein_coding	-0.931111803	1.26E-14	8.27E-13	Down
MARK1	protein_coding	-1.001770923	1.26E-14	8.27E-13	Down
APBB1	protein_coding	-1.090546252	1.34E-14	8.73E-13	Down
RGS9	protein_coding	-1.645789574	1.35E-14	8.76E-13	Down
AL691432.2	long_non_coding	-0.997259584	1.43E-14	9.26E-13	Down
FBXL16	protein_coding	-1.74256013	1.51E-14	9.64E-13	Down
SCARA5	protein_coding	-2.246023875	1.59E-14	1.02E-12	Down
SLC25A53	protein_coding	-0.935482374	1.61E-14	1.02E-12	Down
ZNF710-AS1	long_non_coding	-0.907528503	1.65E-14	1.04E-12	Down
CELF2	protein_coding	-1.115013832	1.66E-14	1.05E-12	Down
C21orf58	protein_coding	-1.314840842	1.71E-14	1.07E-12	Down
ADGRV1	protein_coding	-1.944532818	1.73E-14	1.08E-12	Down
KIAA0319	protein_coding	-1.460307667	1.93E-14	1.20E-12	Down
CD36	protein_coding	-1.474090242	1.93E-14	1.20E-12	Down
PAX6	protein_coding	-1.715125761	2.01E-14	1.23E-12	Down
NRXN1	protein_coding	-1.828002009	2.06E-14	1.26E-12	Down
MAMLD1	protein_coding	-1.281493551	2.08E-14	1.26E-12	Down
AL353743.1	pseudogene	-0.686799586	2.33E-14	1.40E-12	Down
BCL2	protein_coding	-1.033958693	2.72E-14	1.63E-12	Down
GIMAP8	protein_coding	-0.973295757	2.79E-14	1.66E-12	Down
NFATC2	protein_coding	-0.967104875	2.87E-14	1.70E-12	Down
EPB41L3	protein_coding	-1.04707065	2.94E-14	1.73E-12	Down
ATRNL1	protein_coding	-1.850917387	2.94E-14	1.73E-12	Down
SLC8A2	protein_coding	-2.193869586	3.14E-14	1.84E-12	Down
RET	protein_coding	-1.466027362	3.19E-14	1.86E-12	Down
AL596244.1	long_non_coding	-0.846821148	3.20E-14	1.86E-12	Down
EVL	protein_coding	-0.834538434	3.35E-14	1.94E-12	Down
FAM53B	protein_coding	-0.727282851	3.51E-14	2.02E-12	Down
SEC11C	protein_coding	-1.16338596	3.70E-14	2.13E-12	Down
CORO2B	protein_coding	-1.06260856	3.94E-14	2.26E-12	Down
RHOH	protein_coding	-1.403084569	4.04E-14	2.31E-12	Down
PEMT	protein_coding	-1.361697058	4.11E-14	2.33E-12	Down
TAL1	protein_coding	-0.919931002	4.26E-14	2.41E-12	Down
DRC3	protein_coding	-0.880680022	4.46E-14	2.52E-12	Down
AC004656.1	long_non_coding	-0.858430214	4.51E-14	2.53E-12	Down
FBXO10	protein_coding	-0.785404901	4.59E-14	2.56E-12	Down
KIF1A	protein_coding	-2.081911303	4.60E-14	2.56E-12	Down
USP51	protein_coding	-0.905631248	4.75E-14	2.63E-12	Down
PIPOX	protein_coding	-1.416249185	5.08E-14	2.80E-12	Down
HLF	protein_coding	-1.363567883	5.79E-14	3.18E-12	Down
PDK4	protein_coding	-1.483006177	5.83E-14	3.20E-12	Down
CPE	protein_coding	-1.614613473	6.05E-14	3.30E-12	Down
TRAF3IP3	protein_coding	-1.350100807	6.79E-14	3.70E-12	Down
BEX2	protein_coding	-1.548676473	6.99E-14	3.79E-12	Down
TSPYL2	protein_coding	-0.934417564	7.13E-14	3.84E-12	Down
FGF14	protein_coding	-1.532840778	7.53E-14	4.04E-12	Down

LINC00957	long_non_coding	-1.019615828	7.66E-14	4.10E-12	Down
AC005332.7	long_non_coding	-0.839562108	8.56E-14	4.52E-12	Down
TMCC2	protein_coding	-1.120622842	8.90E-14	4.69E-12	Down
RAB9B	protein_coding	-1.11023825	9.00E-14	4.73E-12	Down
LINC00476	long_non_coding	-0.74698285	9.10E-14	4.76E-12	Down
ZMAT1	protein_coding	-1.116385966	9.29E-14	4.82E-12	Down
KCNN3	protein_coding	-1.074268155	9.31E-14	4.82E-12	Down
CD48	protein_coding	-1.499998601	9.42E-14	4.86E-12	Down
FAM46C	protein_coding	-1.210322177	9.52E-14	4.88E-12	Down
CYB5D2	protein_coding	-0.76102835	9.73E-14	4.97E-12	Down
GRAP2	protein_coding	-1.053347408	9.81E-14	5.00E-12	Down
SALL2	protein_coding	-1.136067279	1.02E-13	5.16E-12	Down
RIMS2	protein_coding	-1.92955227	1.14E-13	5.69E-12	Down
DNAJC6	protein_coding	-1.525676689	1.16E-13	5.80E-12	Down
CPEB1	protein_coding	-1.111956681	1.18E-13	5.87E-12	Down
LINC00643	pseudogene	-2.405250193	1.21E-13	5.99E-12	Down
ABCA8	protein_coding	-1.418094203	1.41E-13	6.91E-12	Down
UNC119B	protein_coding	-0.717480965	1.42E-13	6.94E-12	Down
MPZ	protein_coding	-1.142886917	1.44E-13	7.01E-12	Down
AC068473.5	long_non_coding	-0.914923799	1.46E-13	7.08E-12	Down
TMEM59L	protein_coding	-1.703092978	1.46E-13	7.08E-12	Down
KCNJ11	protein_coding	-1.627915834	1.46E-13	7.08E-12	Down
SOBP	protein_coding	-0.911198806	1.60E-13	7.68E-12	Down
SDK1	protein_coding	-1.203859215	1.62E-13	7.75E-12	Down
MAGEE1	protein_coding	-1.145128984	1.63E-13	7.80E-12	Down
MAPK8IP2	protein_coding	-1.698752745	1.64E-13	7.82E-12	Down
SEMA3G	protein_coding	-1.02752686	1.69E-13	8.00E-12	Down
DYNC1I1	protein_coding	-1.395795378	1.70E-13	8.05E-12	Down
RCAN2	protein_coding	-1.00295354	1.71E-13	8.06E-12	Down
ABCA5	protein_coding	-0.898539204	1.74E-13	8.18E-12	Down
DNAJC18	protein_coding	-0.737377422	1.82E-13	8.48E-12	Down
DPH1	protein_coding	-0.61515205	1.83E-13	8.51E-12	Down
SLC25A27	protein_coding	-1.140643649	1.85E-13	8.61E-12	Down
AC073857.1	TEC	-0.881973534	1.87E-13	8.65E-12	Down
CYB561D1	protein_coding	-0.585504901	2.00E-13	9.25E-12	Down
TMOD2	protein_coding	-0.839882267	2.06E-13	9.46E-12	Down
C7	protein_coding	-1.656564471	2.25E-13	1.03E-11	Down
ARHGEF15	protein_coding	-0.843642264	2.43E-13	1.10E-11	Down
MAGEH1	protein_coding	-0.850424619	2.49E-13	1.12E-11	Down
CAMKK1	protein_coding	-0.710787055	2.57E-13	1.14E-11	Down
CHL1	protein_coding	-1.465565082	2.57E-13	1.14E-11	Down
CCND2	protein_coding	-1.079937234	2.74E-13	1.21E-11	Down
GIMAP6	protein_coding	-1.017918476	2.81E-13	1.24E-11	Down
WSCD1	protein_coding	-1.114679169	2.81E-13	1.24E-11	Down
TSPAN11	protein_coding	-1.41282075	2.83E-13	1.24E-11	Down
AKNA	protein_coding	-0.878661163	2.91E-13	1.27E-11	Down
GPR135	protein_coding	-0.989830067	2.99E-13	1.30E-11	Down
ARHGAP15	protein_coding	-1.111369002	3.00E-13	1.30E-11	Down
PNMA8A	protein_coding	-1.430407896	3.03E-13	1.31E-11	Down
TMEM88	protein_coding	-0.884751701	3.07E-13	1.32E-11	Down
NLRC3	protein_coding	-1.025060222	3.08E-13	1.32E-11	Down
SNX22	protein_coding	-1.093661785	3.28E-13	1.41E-11	Down

LY9	protein_coding	-1.532576141	3.30E-13	1.41E-11	Down
IKZF1	protein_coding	-1.360377256	3.39E-13	1.45E-11	Down
AC016705.2	long_non_coding	-1.182765252	3.46E-13	1.47E-11	Down
CAND2	protein_coding	-1.164918071	3.48E-13	1.48E-11	Down
FAM110D	protein_coding	-1.074666405	3.61E-13	1.52E-11	Down
SLC7A2	protein_coding	-1.523612468	3.62E-13	1.53E-11	Down
CERKL	protein_coding	-0.922663869	3.64E-13	1.53E-11	Down
GPR171	protein_coding	-1.426323744	3.67E-13	1.54E-11	Down
USP27X	protein_coding	-1.039846048	4.03E-13	1.68E-11	Down
AP001486.2	long_non_coding	-0.881631253	4.08E-13	1.70E-11	Down
FAM117A	protein_coding	-0.858002514	4.11E-13	1.70E-11	Down
NEXMIF	protein_coding	-1.40971283	4.16E-13	1.72E-11	Down
ZNF594	protein_coding	-0.735342349	4.23E-13	1.74E-11	Down
KATNAL2	protein_coding	-0.842903019	4.32E-13	1.77E-11	Down
SH2D3C	protein_coding	-0.786116837	4.49E-13	1.83E-11	Down
NRCAM	protein_coding	-1.51948067	4.49E-13	1.83E-11	Down
KSR2	protein_coding	-1.372842143	4.61E-13	1.86E-11	Down
TDRP	protein_coding	-0.977471949	4.61E-13	1.86E-11	Down
ELOVL4	protein_coding	-1.222335856	4.69E-13	1.88E-11	Down
CNR1	protein_coding	-1.427940651	5.11E-13	2.04E-11	Down
SYT7	protein_coding	-1.657326509	5.37E-13	2.14E-11	Down
ZC3H12D	protein_coding	-1.212160291	5.59E-13	2.21E-11	Down
NAT8L	protein_coding	-1.681116132	5.65E-13	2.23E-11	Down
RELN	protein_coding	-1.813353377	5.79E-13	2.28E-11	Down
GPRASP2	protein_coding	-0.944732562	6.03E-13	2.37E-11	Down
RCSD1	protein_coding	-1.22480592	6.57E-13	2.55E-11	Down
ADH1B	protein_coding	-1.813904731	6.58E-13	2.55E-11	Down
IPO5P1	pseudogene	-0.959049667	6.81E-13	2.63E-11	Down
KCNJ6	protein_coding	-1.923838217	6.86E-13	2.64E-11	Down
TUB	protein_coding	-0.992966035	6.86E-13	2.64E-11	Down
OSR1	protein_coding	-1.523490801	6.96E-13	2.67E-11	Down
CD200	protein_coding	-1.08881184	7.25E-13	2.78E-11	Down
MAP4K1	protein_coding	-1.303542227	7.29E-13	2.79E-11	Down
CD79A	protein_coding	-2.126078703	7.31E-13	2.79E-11	Down
CERS4	protein_coding	-1.001759155	8.35E-13	3.15E-11	Down
ASPA	protein_coding	-1.137962729	8.74E-13	3.29E-11	Down
WASF3	protein_coding	-0.814650363	8.75E-13	3.29E-11	Down
FAM78A	protein_coding	-0.996128741	8.85E-13	3.31E-11	Down
AC005332.6	long_non_coding	-0.602096761	9.05E-13	3.38E-11	Down
ENPP2	protein_coding	-1.34427728	9.14E-13	3.41E-11	Down
KCNH2	protein_coding	-1.582461955	9.17E-13	3.41E-11	Down
CBX6	protein_coding	-0.782466736	9.30E-13	3.45E-11	Down
ZAP70	protein_coding	-1.375424213	9.49E-13	3.51E-11	Down
ICAM3	protein_coding	-1.368600306	9.54E-13	3.52E-11	Down
AP001528.3	TEC	-0.972416777	9.80E-13	3.61E-11	Down
NLRP1	protein_coding	-1.083509787	9.90E-13	3.64E-11	Down
CLEC10A	protein_coding	-1.402590732	1.06E-12	3.89E-11	Down
SETBP1	protein_coding	-0.871307157	1.10E-12	4.00E-11	Down
COQ10A	protein_coding	-0.676169049	1.11E-12	4.04E-11	Down
GRASP	protein_coding	-0.844822538	1.24E-12	4.45E-11	Down
S1PR1	protein_coding	-0.909184326	1.28E-12	4.59E-11	Down
TP53INP1	protein_coding	-0.682881722	1.30E-12	4.65E-11	Down

TMX4	protein_coding	-0.757339673	1.34E-12	4.77E-11	Down
CACNA1H	protein_coding	-1.073967661	1.37E-12	4.89E-11	Down
KLKB1	protein_coding	-1.511348057	1.50E-12	5.29E-11	Down
MAP6	protein_coding	-1.340578582	1.53E-12	5.37E-11	Down
SLIT3	protein_coding	-1.264341047	1.54E-12	5.40E-11	Down
CLEC1A	protein_coding	-0.888601458	1.59E-12	5.57E-11	Down
44259	protein_coding	-1.854281232	1.62E-12	5.65E-11	Down
TBC1D10C	protein_coding	-1.331713021	1.66E-12	5.77E-11	Down
NT5M	protein_coding	-0.993753519	1.78E-12	6.14E-11	Down
SLC22A17	protein_coding	-1.233132599	1.84E-12	6.32E-11	Down
F10	protein_coding	-1.481821095	1.89E-12	6.49E-11	Down
AL662844.4	long_non_coding	-0.840455888	2.02E-12	6.90E-11	Down
CADM3	protein_coding	-1.591199892	2.03E-12	6.91E-11	Down
TESPA1	protein_coding	-1.31834451	2.04E-12	6.91E-11	Down
ZNF542P	pseudogene	-0.818560341	2.04E-12	6.91E-11	Down
NLGN4X	protein_coding	-1.411705836	2.06E-12	6.96E-11	Down
AC084033.3	long_non_coding	-1.060956191	2.16E-12	7.27E-11	Down
MS4A1	protein_coding	-2.641848961	2.20E-12	7.40E-11	Down
SSTR3	protein_coding	-2.079514548	2.50E-12	8.36E-11	Down
CD1D	protein_coding	-1.003080763	2.63E-12	8.76E-11	Down
TDRD6	protein_coding	-1.024315415	2.63E-12	8.76E-11	Down
SLC9A9	protein_coding	-0.958405998	2.67E-12	8.86E-11	Down
PRKN	protein_coding	-0.692046734	2.83E-12	9.38E-11	Down
CD37	protein_coding	-1.329475007	2.84E-12	9.38E-11	Down
KLRB1	protein_coding	-1.202970277	2.97E-12	9.75E-11	Down
PRKAR2B	protein_coding	-1.000413027	3.26E-12	1.07E-10	Down
WDR17	protein_coding	-1.482837476	3.27E-12	1.07E-10	Down
CD5	protein_coding	-1.230505294	3.30E-12	1.07E-10	Down
ANGEL1	protein_coding	-0.669019756	3.36E-12	1.09E-10	Down
KIAA1324	protein_coding	-1.83265404	3.45E-12	1.11E-10	Down
MAP7D2	protein_coding	-1.49258765	3.45E-12	1.11E-10	Down
SLC46A1	protein_coding	-0.866647583	3.54E-12	1.14E-10	Down
IKZF3	protein_coding	-1.36905013	3.55E-12	1.14E-10	Down
ITM2A	protein_coding	-1.158707773	3.58E-12	1.14E-10	Down
RASAL3	protein_coding	-1.088516628	3.61E-12	1.15E-10	Down
POU2F2	protein_coding	-1.099904592	3.64E-12	1.16E-10	Down
WNT4	protein_coding	-1.700851863	3.67E-12	1.17E-10	Down
DSCAML1	protein_coding	-1.553242176	3.92E-12	1.24E-10	Down
PDE2A	protein_coding	-0.927508395	4.03E-12	1.27E-10	Down
GGTA1P	pseudogene	-1.011701217	4.19E-12	1.32E-10	Down
GNAL	protein_coding	-0.851513078	4.29E-12	1.34E-10	Down
FBLN5	protein_coding	-0.961405124	4.29E-12	1.34E-10	Down
EFNB3	protein_coding	-1.423174825	4.32E-12	1.35E-10	Down
IGHD	IG	-2.441367018	4.39E-12	1.37E-10	Down
PWAR6	long_non_coding	-1.09959803	4.45E-12	1.38E-10	Down
CCDC69	protein_coding	-1.063819598	4.47E-12	1.39E-10	Down
LINC01128	long_non_coding	-0.65967743	4.54E-12	1.40E-10	Down
GVINP1	pseudogene	-1.342323873	4.68E-12	1.44E-10	Down
ADA2	protein_coding	-1.0379383	4.84E-12	1.48E-10	Down
PTPRS	protein_coding	-0.887770662	4.93E-12	1.51E-10	Down
ITGA7	protein_coding	-0.981551537	5.03E-12	1.53E-10	Down
SCAMP5	protein_coding	-1.245819082	5.28E-12	1.60E-10	Down

PCSK2	protein_coding	-2.382833915	5.66E-12	1.71E-10	Down
PIM2	protein_coding	-0.830174593	5.73E-12	1.72E-10	Down
SIT1	protein_coding	-1.442439125	5.73E-12	1.72E-10	Down
CFAP70	protein_coding	-1.190408681	5.73E-12	1.72E-10	Down
LIFR	protein_coding	-0.982792704	6.19E-12	1.86E-10	Down
GSTA4	protein_coding	-0.680078914	6.74E-12	2.01E-10	Down
LRRC4B	protein_coding	-1.334783181	7.04E-12	2.09E-10	Down
FAM120C	protein_coding	-0.636099661	7.04E-12	2.09E-10	Down
SRPX	protein_coding	-1.092868393	7.28E-12	2.16E-10	Down
NIPSNAP3B	protein_coding	-0.659736179	7.32E-12	2.17E-10	Down
CIRBP	protein_coding	-0.702321581	7.44E-12	2.20E-10	Down
SLA2	protein_coding	-1.017523365	7.56E-12	2.22E-10	Down
ELAC1	protein_coding	-0.672221418	7.81E-12	2.29E-10	Down
EID2B	protein_coding	-0.942822545	8.14E-12	2.38E-10	Down
SDCBP2-AS1	long_non_coding	-0.714839858	8.20E-12	2.39E-10	Down
CHGA	protein_coding	-2.225706373	8.22E-12	2.39E-10	Down
RASGRF1	protein_coding	-1.655449003	8.32E-12	2.42E-10	Down
PRRT3	protein_coding	-1.106311279	8.39E-12	2.43E-10	Down
GCH1	protein_coding	-1.064847402	8.65E-12	2.50E-10	Down
IRF4	protein_coding	-1.454947816	8.69E-12	2.51E-10	Down
TMEM150C	protein_coding	-0.855444072	8.73E-12	2.51E-10	Down
GNG4	protein_coding	-1.628540737	8.78E-12	2.53E-10	Down
TRIM9	protein_coding	-1.368886419	9.52E-12	2.73E-10	Down
NRROS	protein_coding	-0.866001175	1.01E-11	2.89E-10	Down
CAVIN2	protein_coding	-1.04340352	1.01E-11	2.89E-10	Down
ATP2A3	protein_coding	-1.217206999	1.02E-11	2.90E-10	Down
44444	protein_coding	-1.00088361	1.03E-11	2.92E-10	Down
CEP126	protein_coding	-1.044610481	1.04E-11	2.95E-10	Down
WDFY4	protein_coding	-1.311072954	1.07E-11	3.02E-10	Down
CD247	protein_coding	-1.157703433	1.07E-11	3.02E-10	Down
TNFSF8	protein_coding	-1.36214476	1.09E-11	3.08E-10	Down
GC	protein_coding	-2.022362046	1.16E-11	3.24E-10	Down
SCN1B	protein_coding	-0.829609615	1.16E-11	3.25E-10	Down
SCGN	protein_coding	-2.11978892	1.18E-11	3.30E-10	Down
RUBCNL	protein_coding	-1.096147363	1.18E-11	3.30E-10	Down
AKAP6	protein_coding	-1.273703832	1.20E-11	3.35E-10	Down
RAB26	protein_coding	-1.505459983	1.22E-11	3.39E-10	Down
EMCN	protein_coding	-0.964075946	1.24E-11	3.42E-10	Down
PREX1	protein_coding	-0.777718445	1.24E-11	3.43E-10	Down
B4GAT1	protein_coding	-0.733302129	1.25E-11	3.45E-10	Down
PACRG	protein_coding	-1.345274095	1.26E-11	3.46E-10	Down
USP2	protein_coding	-1.242692008	1.28E-11	3.52E-10	Down
C15orf59	protein_coding	-1.152800506	1.32E-11	3.61E-10	Down
IL33	protein_coding	-1.199652097	1.33E-11	3.63E-10	Down
ATP1B2	protein_coding	-0.987135005	1.34E-11	3.65E-10	Down
TRBV28	TR	-1.298128709	1.38E-11	3.73E-10	Down
SLC43A2	protein_coding	-0.693541224	1.38E-11	3.73E-10	Down
AQP7	protein_coding	-1.119645938	1.42E-11	3.82E-10	Down
JAKMIP2	protein_coding	-1.273578031	1.44E-11	3.86E-10	Down
UBE2QL1	protein_coding	-1.38260406	1.46E-11	3.92E-10	Down
AGTR1	protein_coding	-1.200407552	1.48E-11	3.96E-10	Down
ZNF471	protein_coding	-0.883996032	1.50E-11	4.01E-10	Down

LONRF2	protein_coding	-1.137846127	1.59E-11	4.20E-10	Down
ZNF674-AS1	long_non_coding	-0.767627818	1.60E-11	4.22E-10	Down
MCOLN3	protein_coding	-1.428196572	1.61E-11	4.25E-10	Down
GPM6B	protein_coding	-1.077530827	1.68E-11	4.43E-10	Down
MEG3	long_non_coding	-1.283043089	1.75E-11	4.59E-10	Down
FABP4	protein_coding	-2.044251105	1.77E-11	4.64E-10	Down
HCLS1	protein_coding	-0.961344733	1.91E-11	4.98E-10	Down
BAIAP3	protein_coding	-1.560768119	1.92E-11	5.01E-10	Down
TMC8	protein_coding	-1.119056684	1.99E-11	5.18E-10	Down
AL645608.1	long_non_coding	-1.035178407	2.06E-11	5.32E-10	Down
CXCL12	protein_coding	-1.239767458	2.10E-11	5.42E-10	Down
RASSF8-AS1	long_non_coding	-0.729710489	2.20E-11	5.64E-10	Down
KBTBD8	protein_coding	-1.001524338	2.21E-11	5.66E-10	Down
CLEC3B	protein_coding	-1.044717471	2.23E-11	5.68E-10	Down
RASIP1	protein_coding	-0.708528346	2.24E-11	5.68E-10	Down
FLI1	protein_coding	-0.861356456	2.27E-11	5.76E-10	Down
SYNGR1	protein_coding	-0.847418516	2.28E-11	5.78E-10	Down
LGI2	protein_coding	-1.041221001	2.39E-11	6.04E-10	Down
ATP6V0E2	protein_coding	-1.011834085	2.49E-11	6.27E-10	Down
BEND5	protein_coding	-0.840464875	2.52E-11	6.33E-10	Down
METTL7A	protein_coding	-0.846425185	2.53E-11	6.35E-10	Down
SGSM2	protein_coding	-0.717218569	2.62E-11	6.54E-10	Down
SLC8A1	protein_coding	-1.054700637	2.68E-11	6.67E-10	Down
TUNAR	protein_coding	-1.969529677	2.69E-11	6.67E-10	Down
RBP7	protein_coding	-1.047411102	2.90E-11	7.16E-10	Down
KLHDC1	protein_coding	-0.709613684	2.96E-11	7.31E-10	Down
CD27	protein_coding	-1.352567444	2.98E-11	7.35E-10	Down
ARHGAP9	protein_coding	-1.049696321	3.03E-11	7.43E-10	Down
AP002004.1	pseudogene	-0.818759773	3.04E-11	7.44E-10	Down
AGT	protein_coding	-1.352867231	3.11E-11	7.58E-10	Down
CCL21	protein_coding	-1.782727949	3.13E-11	7.62E-10	Down
STXBP1	protein_coding	-0.953561971	3.20E-11	7.78E-10	Down
KCNMA1	protein_coding	-1.22500235	3.22E-11	7.80E-10	Down
ZSCAN18	protein_coding	-0.851260117	3.30E-11	7.98E-10	Down
FHL1	protein_coding	-1.079042331	3.48E-11	8.37E-10	Down
SH2D1A	protein_coding	-1.380049875	3.49E-11	8.38E-10	Down
SYNE1	protein_coding	-0.797977974	3.63E-11	8.69E-10	Down
ASTN2	protein_coding	-0.787590101	3.72E-11	8.89E-10	Down
SLC25A34	protein_coding	-1.070097314	3.75E-11	8.94E-10	Down
NBEA	protein_coding	-1.111597087	3.91E-11	9.30E-10	Down
SHE	protein_coding	-0.824028009	3.92E-11	9.32E-10	Down
NACAD	protein_coding	-1.214448023	3.95E-11	9.38E-10	Down
ZNF660	protein_coding	-0.716014713	4.19E-11	9.91E-10	Down
ABCA10	protein_coding	-0.875074695	4.21E-11	9.96E-10	Down
BACH2	protein_coding	-1.006046868	4.48E-11	1.06E-09	Down
FLJ37453	long_non_coding	-0.605900958	4.52E-11	1.06E-09	Down
PBX3	protein_coding	-0.680671932	4.54E-11	1.07E-09	Down
PEG3	protein_coding	-1.111523972	4.89E-11	1.15E-09	Down
RPH3AL	protein_coding	-0.994593702	4.97E-11	1.16E-09	Down
EBF1	protein_coding	-0.835701511	4.97E-11	1.16E-09	Down
LYVE1	protein_coding	-1.30253473	5.01E-11	1.17E-09	Down
IL10RA	protein_coding	-0.9096779	5.13E-11	1.19E-09	Down

ATP8A1	protein_coding	-1.054426656	5.25E-11	1.22E-09	Down
PCSK1N	protein_coding	-2.043373894	5.30E-11	1.23E-09	Down
SYNE3	protein_coding	-0.82847867	5.40E-11	1.25E-09	Down
GAD2	protein_coding	-2.195535625	5.78E-11	1.33E-09	Down
FZD4	protein_coding	-0.66611047	5.92E-11	1.36E-09	Down
NCALD	protein_coding	-0.829793654	6.10E-11	1.40E-09	Down
GAPT	protein_coding	-1.257495554	6.14E-11	1.40E-09	Down
BEX5	protein_coding	-1.232580793	6.25E-11	1.42E-09	Down
HSPB6	protein_coding	-1.216869388	6.38E-11	1.45E-09	Down
CORO1A	protein_coding	-1.111662922	6.60E-11	1.49E-09	Down
LINC01963	long_non_coding	-0.72919656	6.69E-11	1.51E-09	Down
ZNF441	protein_coding	-0.643137629	6.97E-11	1.57E-09	Down
GNB3	protein_coding	-0.994166059	7.28E-11	1.64E-09	Down
GIMAP4	protein_coding	-0.835243773	7.40E-11	1.66E-09	Down
ABCC9	protein_coding	-1.094833507	7.44E-11	1.67E-09	Down
AL035071.1	long_non_coding	-0.893954839	7.49E-11	1.68E-09	Down
ABCA9	protein_coding	-1.028255081	7.53E-11	1.68E-09	Down
SLC6A17	protein_coding	-1.723948319	7.59E-11	1.69E-09	Down
CD28	protein_coding	-1.283900721	7.77E-11	1.72E-09	Down
LINC00663	long_non_coding	-0.606558218	7.96E-11	1.76E-09	Down
SLC7A8	protein_coding	-1.111630085	8.08E-11	1.78E-09	Down
NGFR	protein_coding	-1.376726166	8.38E-11	1.83E-09	Down
RFX2	protein_coding	-0.879408154	8.39E-11	1.83E-09	Down
SNHG14	long_non_coding	-0.882883564	8.44E-11	1.84E-09	Down
KPNA5	protein_coding	-0.596944839	8.67E-11	1.88E-09	Down
C17orf51	protein_coding	-0.791184245	9.11E-11	1.97E-09	Down
FOXP2	protein_coding	-1.148999009	9.23E-11	1.99E-09	Down
CHGB	protein_coding	-2.122479893	9.24E-11	1.99E-09	Down
CADPS	protein_coding	-1.411164413	9.30E-11	2.00E-09	Down
IRAK1BP1	protein_coding	-0.59146586	1.04E-10	2.21E-09	Down
SMIM27	protein_coding	-0.719595401	1.05E-10	2.25E-09	Down
FSD1L	protein_coding	-0.734971022	1.08E-10	2.31E-09	Down
PCBP3	protein_coding	-0.962001549	1.09E-10	2.33E-09	Down
CRMP1	protein_coding	-1.009681945	1.10E-10	2.35E-09	Down
VGF	protein_coding	-1.846518807	1.11E-10	2.36E-09	Down
NRSN2	protein_coding	-0.784026226	1.15E-10	2.43E-09	Down
DLG4	protein_coding	-0.919049246	1.21E-10	2.55E-09	Down
EDNRB	protein_coding	-0.924880654	1.26E-10	2.64E-09	Down
ADGRL1	protein_coding	-0.89933703	1.27E-10	2.65E-09	Down
EDA	protein_coding	-0.952642173	1.28E-10	2.66E-09	Down
PPP1R3E	protein_coding	-0.719414382	1.28E-10	2.67E-09	Down
SASH3	protein_coding	-1.082432082	1.30E-10	2.69E-09	Down
PLIN4	protein_coding	-1.909082362	1.30E-10	2.70E-09	Down
SLC23A2	protein_coding	-0.597280828	1.36E-10	2.82E-09	Down
APOLD1	protein_coding	-0.870688547	1.38E-10	2.85E-09	Down
C16orf89	protein_coding	-1.379622312	1.43E-10	2.95E-09	Down
DES	protein_coding	-2.094078179	1.44E-10	2.96E-09	Down
AC104825.2	long_non_coding	-0.63825704	1.45E-10	2.98E-09	Down
LMO3	protein_coding	-1.270290214	1.46E-10	2.99E-09	Down
CLIP3	protein_coding	-0.886963282	1.48E-10	3.03E-09	Down
C1QL1	protein_coding	-1.550278719	1.58E-10	3.22E-09	Down
CNKSR2	protein_coding	-1.092990412	1.59E-10	3.23E-09	Down

U91328.2	long_non_coding	-0.63892336	1.61E-10	3.26E-09	Down
LIMD2	protein_coding	-1.019586341	1.68E-10	3.39E-09	Down
LRP2BP	protein_coding	-0.885070385	1.69E-10	3.42E-09	Down
TOM1L2	protein_coding	-0.598590101	1.72E-10	3.47E-09	Down
NXPE3	protein_coding	-0.705670385	1.75E-10	3.52E-09	Down
TTLL7	protein_coding	-0.976110825	1.76E-10	3.53E-09	Down
STAG3	protein_coding	-0.912665828	1.80E-10	3.60E-09	Down
FGFBP3	protein_coding	-0.906929066	1.92E-10	3.82E-09	Down
ZIK1	protein_coding	-0.819046842	1.95E-10	3.88E-09	Down
AC002546.1	long_non_coding	-0.930416823	1.96E-10	3.91E-09	Down
CD3E	protein_coding	-1.207447504	2.00E-10	3.96E-09	Down
QDPR	protein_coding	-0.853243	2.04E-10	4.03E-09	Down
LYRM9	protein_coding	-0.634089352	2.06E-10	4.06E-09	Down
NAP1L3	protein_coding	-0.985686833	2.07E-10	4.07E-09	Down
PSD	protein_coding	-1.084965532	2.07E-10	4.07E-09	Down
RGL1	protein_coding	-0.673467901	2.07E-10	4.07E-09	Down
PDE3B	protein_coding	-1.087753863	2.07E-10	4.07E-09	Down
DOCK10	protein_coding	-0.909752429	2.11E-10	4.14E-09	Down
AC093278.2	long_non_coding	-0.712242324	2.13E-10	4.16E-09	Down
CA8	protein_coding	-1.18998783	2.14E-10	4.19E-09	Down
GAB3	protein_coding	-0.774286343	2.15E-10	4.20E-09	Down
ANGPTL1	protein_coding	-1.291558402	2.17E-10	4.23E-09	Down
SELP	protein_coding	-1.137916252	2.27E-10	4.41E-09	Down
C14orf132	protein_coding	-1.025033676	2.27E-10	4.41E-09	Down
SLAMF1	protein_coding	-1.185227104	2.36E-10	4.57E-09	Down
TRAF1	protein_coding	-0.652208102	2.39E-10	4.61E-09	Down
AC097639.1	long_non_coding	-1.018034929	2.44E-10	4.70E-09	Down
TSPOAP1	protein_coding	-0.923571886	2.48E-10	4.76E-09	Down
ZSCAN16-AS1	long_non_coding	-0.756291358	2.54E-10	4.87E-09	Down
NCF1	protein_coding	-1.137434373	2.55E-10	4.87E-09	Down
MAN1C1	protein_coding	-0.778954481	2.64E-10	5.03E-09	Down
CCDC3	protein_coding	-0.754493555	2.65E-10	5.05E-09	Down
SAT2	protein_coding	-0.58732761	2.69E-10	5.10E-09	Down
AL162377.1	long_non_coding	-0.770114894	2.71E-10	5.14E-09	Down
RAMP2	protein_coding	-0.71582192	2.73E-10	5.17E-09	Down
RAMP3	protein_coding	-0.771492157	2.77E-10	5.24E-09	Down
KCTD12	protein_coding	-0.746780973	2.89E-10	5.43E-09	Down
JAM2	protein_coding	-0.782576011	3.03E-10	5.66E-09	Down
REV3L	protein_coding	-0.626311698	3.03E-10	5.66E-09	Down
IL16	protein_coding	-1.035408406	3.05E-10	5.70E-09	Down
ZNF10	protein_coding	-0.631291151	3.12E-10	5.81E-09	Down
KCNK3	protein_coding	-1.600958947	3.28E-10	6.08E-09	Down
PALMD	protein_coding	-0.824306095	3.35E-10	6.19E-09	Down
PITPNA-AS1	long_non_coding	-0.666797769	3.41E-10	6.28E-09	Down
GPAM	protein_coding	-0.860104971	3.45E-10	6.35E-09	Down
AC022916.1	long_non_coding	-0.618272997	3.50E-10	6.42E-09	Down
RASSF2	protein_coding	-0.897457451	3.78E-10	6.92E-09	Down
IGKV1-16	IG	-1.758273671	3.97E-10	7.23E-09	Down
GJD2	protein_coding	-2.151436258	4.03E-10	7.32E-09	Down
CCM2L	protein_coding	-0.704091388	4.19E-10	7.56E-09	Down
RNASE6	protein_coding	-0.934546218	4.20E-10	7.57E-09	Down
AC245297.1	pseudogene	-1.062241897	4.30E-10	7.73E-09	Down

GPC3	protein_coding	-0.923995864	4.32E-10	7.76E-09	Down
ZNF382	protein_coding	-0.729293911	4.42E-10	7.93E-09	Down
WNK4	protein_coding	-1.653762731	4.45E-10	7.97E-09	Down
SCRG1	protein_coding	-1.379075579	4.46E-10	7.97E-09	Down
CCL2	protein_coding	-1.114929504	4.48E-10	8.00E-09	Down
PPP2R2B	protein_coding	-1.09057931	4.63E-10	8.26E-09	Down
IL11RA	protein_coding	-0.630894111	4.85E-10	8.62E-09	Down
TLE2	protein_coding	-0.70733272	5.00E-10	8.87E-09	Down
GPM6A	protein_coding	-1.461983336	5.09E-10	9.01E-09	Down
AMIGO1	protein_coding	-0.872174143	5.15E-10	9.11E-09	Down
FAM110B	protein_coding	-0.715293529	5.21E-10	9.19E-09	Down
RAP1GAP2	protein_coding	-0.932616558	5.27E-10	9.28E-09	Down
CHST10	protein_coding	-0.63270509	5.33E-10	9.37E-09	Down
SMAD9	protein_coding	-0.905606152	5.40E-10	9.49E-09	Down
LY6G5C	protein_coding	-0.65738618	5.43E-10	9.52E-09	Down
FITM2	protein_coding	-0.617394123	5.61E-10	9.81E-09	Down
ARHGAP30	protein_coding	-0.848906676	5.71E-10	9.98E-09	Down
EXOC3-AS1	long_non_coding	-0.76817282	5.74E-10	1.00E-08	Down
CD226	protein_coding	-0.963745584	5.76E-10	1.00E-08	Down
MAP2	protein_coding	-1.097113148	5.77E-10	1.00E-08	Down
FDCSP	protein_coding	-2.160108165	5.84E-10	1.01E-08	Down
ARHGAP25	protein_coding	-0.872696141	5.85E-10	1.01E-08	Down
JAK3	protein_coding	-0.816752152	5.97E-10	1.03E-08	Down
ERG	protein_coding	-0.752306291	6.00E-10	1.04E-08	Down
CACNA2D3	protein_coding	-1.141189515	6.13E-10	1.06E-08	Down
ELL2	protein_coding	-0.771705309	6.33E-10	1.09E-08	Down
ACVR1C	protein_coding	-1.143498805	6.44E-10	1.11E-08	Down
HSPB8	protein_coding	-0.942130021	6.62E-10	1.14E-08	Down
IFFO1	protein_coding	-0.593691442	6.94E-10	1.19E-08	Down
DENND2A	protein_coding	-0.768093137	6.95E-10	1.19E-08	Down
ZNF135	protein_coding	-0.71739317	7.37E-10	1.26E-08	Down
PCED1B-AS1	long_non_coding	-1.004503549	7.43E-10	1.26E-08	Down
SOWAHA	protein_coding	-1.107210062	7.45E-10	1.27E-08	Down
CD3D	protein_coding	-1.173266002	7.50E-10	1.27E-08	Down
POU2AF1	protein_coding	-1.477657875	7.63E-10	1.29E-08	Down
FCN3	protein_coding	-0.906140999	7.78E-10	1.32E-08	Down
PMM1	protein_coding	-0.625823472	8.03E-10	1.36E-08	Down
NFATC1	protein_coding	-0.814383767	8.07E-10	1.36E-08	Down
COL14A1	protein_coding	-1.128041402	8.44E-10	1.42E-08	Down
CHI3L2	protein_coding	-1.261673133	8.55E-10	1.44E-08	Down
CXCR4	protein_coding	-1.03508659	8.56E-10	1.44E-08	Down
TOGARAM2	protein_coding	-1.309121295	8.60E-10	1.44E-08	Down
HLA-DOB	protein_coding	-1.256346091	8.81E-10	1.47E-08	Down
CYSLTR1	protein_coding	-0.931735128	9.09E-10	1.52E-08	Down
CPT1C	protein_coding	-0.818482291	9.11E-10	1.52E-08	Down
CD6	protein_coding	-0.99106471	9.13E-10	1.52E-08	Down
LHX6	protein_coding	-0.707934966	9.36E-10	1.55E-08	Down
GRK3	protein_coding	-0.660611565	1.02E-09	1.67E-08	Down
TMPRSS6	protein_coding	-1.301208714	1.02E-09	1.68E-08	Down
MAFB	protein_coding	-1.108986338	1.15E-09	1.87E-08	Down
MYCT1	protein_coding	-0.732063649	1.15E-09	1.88E-08	Down
CACNA1D	protein_coding	-0.889850051	1.17E-09	1.90E-08	Down

ZFP82	protein_coding	-0.705774932	1.17E-09	1.90E-08	Down
AL109811.3	long_non_coding	-0.813535007	1.20E-09	1.93E-08	Down
ZDHHC15	protein_coding	-0.772399532	1.20E-09	1.93E-08	Down
NME5	protein_coding	-0.951504892	1.20E-09	1.94E-08	Down
C17orf107	protein_coding	-0.756827107	1.23E-09	1.98E-08	Down
KCNT2	protein_coding	-0.944214092	1.26E-09	2.02E-08	Down
TRO	protein_coding	-0.806698334	1.26E-09	2.02E-08	Down
RNF157-AS1	long_non_coding	-1.085426638	1.27E-09	2.03E-08	Down
ROBO4	protein_coding	-0.585491187	1.36E-09	2.17E-08	Down
ZNF132	protein_coding	-0.663859199	1.37E-09	2.18E-08	Down
GSTM2	protein_coding	-0.757793747	1.37E-09	2.19E-08	Down
FGD3	protein_coding	-0.817914735	1.39E-09	2.21E-08	Down
PRIMA1	protein_coding	-1.458252702	1.41E-09	2.24E-08	Down
OGN	protein_coding	-1.467901192	1.41E-09	2.24E-08	Down
WAS	protein_coding	-0.900548619	1.44E-09	2.27E-08	Down
KCNK16	protein_coding	-2.210973835	1.48E-09	2.34E-08	Down
SOX17	protein_coding	-0.832318575	1.50E-09	2.36E-08	Down
MYRIP	protein_coding	-1.158294248	1.52E-09	2.39E-08	Down
MPP1	protein_coding	-0.695580551	1.56E-09	2.44E-08	Down
ACKR1	protein_coding	-1.402758805	1.57E-09	2.45E-08	Down
APOH	protein_coding	-1.684003904	1.62E-09	2.53E-08	Down
LINC00261	long_non_coding	-1.52318788	1.66E-09	2.58E-08	Down
TMED8	protein_coding	-0.641220704	1.66E-09	2.58E-08	Down
PIK3R5	protein_coding	-0.915018896	1.67E-09	2.59E-08	Down
PDE4B	protein_coding	-0.851908266	1.69E-09	2.62E-08	Down
MAN1A1	protein_coding	-0.800145692	1.79E-09	2.77E-08	Down
TNXB	protein_coding	-1.112443931	1.85E-09	2.84E-08	Down
KLHL6	protein_coding	-1.064814432	1.86E-09	2.86E-08	Down
EGFL7	protein_coding	-0.827459244	1.87E-09	2.86E-08	Down
GDAP1	protein_coding	-0.820465303	1.91E-09	2.91E-08	Down
AP002360.2	long_non_coding	-0.759613585	1.92E-09	2.93E-08	Down
EPHX2	protein_coding	-0.88085289	1.94E-09	2.96E-08	Down
FREM1	protein_coding	-1.150742048	2.05E-09	3.10E-08	Down
FCRL5	protein_coding	-1.57828715	2.05E-09	3.10E-08	Down
PDE1C	protein_coding	-0.868785107	2.13E-09	3.21E-08	Down
HSD17B14	protein_coding	-0.905345777	2.16E-09	3.25E-08	Down
B9D1	protein_coding	-0.818874824	2.16E-09	3.25E-08	Down
TAGAP	protein_coding	-1.020019316	2.17E-09	3.26E-08	Down
PPP1R3F	protein_coding	-0.868724191	2.24E-09	3.35E-08	Down
44445	protein_coding	-0.659437353	2.26E-09	3.38E-08	Down
PTPRC	protein_coding	-1.178236992	2.32E-09	3.46E-08	Down
CXCR6	protein_coding	-1.04626554	2.39E-09	3.55E-08	Down
NKX6-1	protein_coding	-1.409298562	2.50E-09	3.70E-08	Down
SLC16A12	protein_coding	-1.686543615	2.51E-09	3.72E-08	Down
TRAM1L1	protein_coding	-0.789751948	2.53E-09	3.73E-08	Down
FAM167A	protein_coding	-1.063521081	2.58E-09	3.80E-08	Down
RBP5	protein_coding	-0.822545857	2.60E-09	3.82E-08	Down
TEK	protein_coding	-0.798805751	2.66E-09	3.90E-08	Down
NAPB	protein_coding	-0.738625491	2.75E-09	4.02E-08	Down
OLFM1	protein_coding	-1.074340042	2.76E-09	4.03E-08	Down
LRRC37A3	protein_coding	-0.794675731	2.76E-09	4.03E-08	Down
DPY19L2	protein_coding	-1.079225638	2.77E-09	4.04E-08	Down

WDR7	protein_coding	-0.587818324	2.80E-09	4.08E-08	Down
NTRK3	protein_coding	-1.087101798	2.93E-09	4.25E-08	Down
SCN7A	protein_coding	-1.340879101	2.93E-09	4.25E-08	Down
RNF150	protein_coding	-0.939262923	2.99E-09	4.32E-08	Down
TGFBR3	protein_coding	-0.824412309	3.01E-09	4.34E-08	Down
FGF13	protein_coding	-1.063169024	3.01E-09	4.35E-08	Down
GPD1	protein_coding	-1.352183182	3.06E-09	4.40E-08	Down
TVP23A	protein_coding	-0.677763811	3.27E-09	4.69E-08	Down
TIAM1	protein_coding	-0.845341313	3.28E-09	4.70E-08	Down
LINC00641	long_non_coding	-0.750133552	3.32E-09	4.75E-08	Down
ARFGEF3	protein_coding	-1.114182877	3.34E-09	4.77E-08	Down
MEDAG	protein_coding	-1.356166938	3.36E-09	4.80E-08	Down
LRRN2	protein_coding	-0.959358163	3.40E-09	4.84E-08	Down
PTGDS	protein_coding	-1.201186014	3.44E-09	4.89E-08	Down
SLC35F3	protein_coding	-1.422486046	3.45E-09	4.90E-08	Down
BTK	protein_coding	-0.989698062	3.54E-09	5.02E-08	Down
AC105942.1	long_non_coding	-0.586452777	3.55E-09	5.02E-08	Down
GZMK	protein_coding	-1.283869825	3.55E-09	5.02E-08	Down
SOX5	protein_coding	-0.829937829	3.61E-09	5.09E-08	Down
EBI3	protein_coding	-0.968832705	3.65E-09	5.15E-08	Down
CR1	protein_coding	-1.31962702	3.66E-09	5.15E-08	Down
ITGAL	protein_coding	-1.057118202	3.66E-09	5.15E-08	Down
CXCL13	protein_coding	-2.01848607	3.68E-09	5.17E-08	Down
PGBD5	protein_coding	-0.921505626	3.71E-09	5.21E-08	Down
ST8SIA1	protein_coding	-0.961835534	3.74E-09	5.25E-08	Down
CLSTN2	protein_coding	-1.108268921	3.76E-09	5.26E-08	Down
TUSC3	protein_coding	-1.004538317	3.80E-09	5.31E-08	Down
STAB1	protein_coding	-0.680562397	3.86E-09	5.38E-08	Down
NDRG4	protein_coding	-1.105910793	3.87E-09	5.39E-08	Down
TTLL6	protein_coding	-1.424855739	4.10E-09	5.70E-08	Down
PRRT1	protein_coding	-0.735841444	4.25E-09	5.86E-08	Down
MPEG1	protein_coding	-1.005156431	4.30E-09	5.92E-08	Down
FAM13C	protein_coding	-0.89024658	4.31E-09	5.93E-08	Down
TRAC	TR	-1.044079655	4.32E-09	5.93E-08	Down
TMEM131L	protein_coding	-0.748240729	4.36E-09	5.97E-08	Down
CD3G	protein_coding	-1.126839514	4.50E-09	6.16E-08	Down
AC007066.2	long_non_coding	-0.692932054	4.55E-09	6.21E-08	Down
VAT1L	protein_coding	-1.000874569	4.60E-09	6.28E-08	Down
ESR1	protein_coding	-0.899582057	4.71E-09	6.41E-08	Down
PTPRN2	protein_coding	-1.234476035	4.80E-09	6.52E-08	Down
ZNF853	protein_coding	-0.694641603	4.98E-09	6.75E-08	Down
ADCY5	protein_coding	-0.970078683	5.04E-09	6.81E-08	Down
CD96	protein_coding	-1.046398768	5.13E-09	6.93E-08	Down
FAM169A	protein_coding	-1.028078414	5.20E-09	7.01E-08	Down
CMAHP	pseudogene	-0.692098928	5.27E-09	7.10E-08	Down
PCDHB5	protein_coding	-0.970024201	5.35E-09	7.19E-08	Down
NPAS3	protein_coding	-0.871020502	5.37E-09	7.20E-08	Down
ART4	protein_coding	-1.083981509	5.41E-09	7.25E-08	Down
PARP15	protein_coding	-1.182032079	5.66E-09	7.56E-08	Down
KCNJ5	protein_coding	-1.478681142	5.69E-09	7.58E-08	Down
MLXIPL	protein_coding	-1.310911979	5.71E-09	7.59E-08	Down
LCP1	protein_coding	-0.915429178	5.90E-09	7.82E-08	Down

UBASH3A	protein_coding	-0.981172273	5.90E-09	7.82E-08	Down
SCIMP	protein_coding	-0.981931574	5.98E-09	7.90E-08	Down
SERPINI1	protein_coding	-0.94727773	6.05E-09	7.98E-08	Down
SIRPG	protein_coding	-1.135812781	6.10E-09	8.04E-08	Down
HEPACAM2	protein_coding	-1.565377818	6.18E-09	8.14E-08	Down
USHBP1	protein_coding	-0.690595976	6.25E-09	8.23E-08	Down
SPEF2	protein_coding	-0.730672326	6.28E-09	8.25E-08	Down
TIGIT	protein_coding	-1.195639855	6.31E-09	8.27E-08	Down
RTN4RL1	protein_coding	-1.170352388	6.43E-09	8.42E-08	Down
TCTN1	protein_coding	-0.648725508	6.49E-09	8.48E-08	Down
CCL19	protein_coding	-1.668698912	6.55E-09	8.55E-08	Down
CD7	protein_coding	-0.986213699	6.56E-09	8.55E-08	Down
VASH1	protein_coding	-0.592706251	7.12E-09	9.22E-08	Down
PCLO	protein_coding	-1.193190855	7.26E-09	9.38E-08	Down
TSPYL4	protein_coding	-0.59361424	7.42E-09	9.56E-08	Down
CLU	protein_coding	-1.076188678	7.46E-09	9.61E-08	Down
CYYR1	protein_coding	-0.698678949	7.69E-09	9.89E-08	Down
ASB2	protein_coding	-1.225245507	8.02E-09	1.03E-07	Down
FAM171B	protein_coding	-0.805248896	8.33E-09	1.07E-07	Down
SELE	protein_coding	-1.470642304	9.08E-09	1.15E-07	Down
AC009014.1	protein_coding	-1.518702978	9.16E-09	1.16E-07	Down
CCR4	protein_coding	-1.294426807	9.23E-09	1.17E-07	Down
PELI3	protein_coding	-0.754764441	9.52E-09	1.20E-07	Down
TOX	protein_coding	-1.061805418	9.70E-09	1.22E-07	Down
ADGRG2	protein_coding	-1.376018114	9.72E-09	1.22E-07	Down
FNBP1	protein_coding	-0.607468715	1.01E-08	1.27E-07	Down
FOSB	protein_coding	-1.425732354	1.04E-08	1.30E-07	Down
UCN3	protein_coding	-1.699634306	1.07E-08	1.34E-07	Down
DPEP2	protein_coding	-0.854476187	1.07E-08	1.34E-07	Down
NEGR1	protein_coding	-0.872585374	1.08E-08	1.35E-07	Down
RNF180	protein_coding	-0.759399958	1.09E-08	1.35E-07	Down
SRGAP3	protein_coding	-0.721602061	1.11E-08	1.37E-07	Down
IPCEF1	protein_coding	-0.857072324	1.11E-08	1.38E-07	Down
GPS2	protein_coding	-0.624608194	1.12E-08	1.39E-07	Down
AC046185.3	TEC	-0.760283171	1.17E-08	1.45E-07	Down
DDC	protein_coding	-1.561615093	1.20E-08	1.48E-07	Down
ECSCR	protein_coding	-0.681568516	1.21E-08	1.49E-07	Down
FILIP1	protein_coding	-0.858367565	1.24E-08	1.53E-07	Down
LMO2	protein_coding	-0.590246598	1.25E-08	1.53E-07	Down
BIN2	protein_coding	-0.821092651	1.26E-08	1.55E-07	Down
ADD2	protein_coding	-0.98992647	1.29E-08	1.58E-07	Down
GYPC	protein_coding	-0.69013454	1.33E-08	1.62E-07	Down
TTYH2	protein_coding	-0.657490826	1.35E-08	1.65E-07	Down
SFMBT2	protein_coding	-0.669635317	1.36E-08	1.66E-07	Down
SPOCK2	protein_coding	-1.147053685	1.42E-08	1.72E-07	Down
GALNT13	protein_coding	-1.174116626	1.44E-08	1.74E-07	Down
PIK3CD	protein_coding	-0.716588437	1.45E-08	1.75E-07	Down
PALD1	protein_coding	-0.630743028	1.45E-08	1.75E-07	Down
IGHM	IG	-1.58620086	1.53E-08	1.84E-07	Down
ASXL3	protein_coding	-0.860574378	1.57E-08	1.88E-07	Down
CXorf36	protein_coding	-0.595571434	1.57E-08	1.88E-07	Down
PTGFR	protein_coding	-1.044650161	1.58E-08	1.89E-07	Down

MICU3	protein_coding	-0.785353415	1.58E-08	1.89E-07	Down
GRIK3	protein_coding	-1.144169921	1.61E-08	1.91E-07	Down
KCND3	protein_coding	-0.915523322	1.61E-08	1.92E-07	Down
WDFY3-AS2	long_non_coding	-0.626900334	1.66E-08	1.97E-07	Down
POU6F2	protein_coding	-1.490382668	1.74E-08	2.06E-07	Down
AC011899.2	long_non_coding	-0.834955906	1.74E-08	2.06E-07	Down
RGS11	protein_coding	-1.055331776	1.80E-08	2.12E-07	Down
CUBN	protein_coding	-0.653167055	1.89E-08	2.22E-07	Down
PLA2G4C	protein_coding	-0.61771658	1.91E-08	2.24E-07	Down
IL7R	protein_coding	-1.150247261	1.91E-08	2.24E-07	Down
ADAMTS1	protein_coding	-0.922182379	1.91E-08	2.24E-07	Down
H1FX-AS1	long_non_coding	-0.792381695	1.99E-08	2.33E-07	Down
ST8SIA3	protein_coding	-1.600835062	2.02E-08	2.35E-07	Down
BHLHE22	protein_coding	-0.938759299	2.06E-08	2.40E-07	Down
FGF12	protein_coding	-1.042309472	2.12E-08	2.46E-07	Down
SRCIN1	protein_coding	-1.004579999	2.17E-08	2.51E-07	Down
CCDC181	protein_coding	-0.977161714	2.20E-08	2.54E-07	Down
ZNF569	protein_coding	-0.631694114	2.22E-08	2.56E-07	Down
PRUNE2	protein_coding	-1.322784451	2.27E-08	2.62E-07	Down
KIAA1683	protein_coding	-0.909517305	2.32E-08	2.67E-07	Down
TPPP	protein_coding	-1.013063877	2.34E-08	2.69E-07	Down
MADCAM1	protein_coding	-1.288020356	2.37E-08	2.71E-07	Down
DZIP3	protein_coding	-0.66708711	2.40E-08	2.75E-07	Down
ADCYAP1	protein_coding	-1.41398743	2.44E-08	2.79E-07	Down
AOAH	protein_coding	-0.935038341	2.44E-08	2.79E-07	Down
TTYH1	protein_coding	-1.435821581	2.44E-08	2.79E-07	Down
HDC	protein_coding	-1.006820361	2.48E-08	2.82E-07	Down
RGS4	protein_coding	-1.136865649	2.51E-08	2.85E-07	Down
MMP24	protein_coding	-0.885080541	2.52E-08	2.86E-07	Down
AC022107.1	TEC	-0.728989876	2.55E-08	2.89E-07	Down
CCR2	protein_coding	-1.0424109	2.59E-08	2.93E-07	Down
PCAT19	long_non_coding	-0.683035837	2.64E-08	2.98E-07	Down
CD52	protein_coding	-1.069837031	2.69E-08	3.03E-07	Down
ZNF429	protein_coding	-0.602109578	2.73E-08	3.07E-07	Down
OSBPL6	protein_coding	-0.94982983	2.76E-08	3.08E-07	Down
CLSTN3	protein_coding	-0.611126696	2.79E-08	3.11E-07	Down
CADM1	protein_coding	-0.781554586	2.80E-08	3.12E-07	Down
NEUROD1	protein_coding	-1.732874467	2.82E-08	3.15E-07	Down
AC008969.1	long_non_coding	-0.748459357	2.84E-08	3.16E-07	Down
PRKCQ-AS1	long_non_coding	-0.872112918	2.89E-08	3.21E-07	Down
NRXN3	protein_coding	-1.06259761	2.92E-08	3.25E-07	Down
CD2	protein_coding	-0.987532364	2.93E-08	3.25E-07	Down
FAM124A	protein_coding	-0.723148778	2.95E-08	3.27E-07	Down
CSF2RB	protein_coding	-0.943619663	2.97E-08	3.29E-07	Down
C10orf128	protein_coding	-0.702569593	2.99E-08	3.31E-07	Down
TTC25	protein_coding	-0.880492874	3.06E-08	3.38E-07	Down
SCN4B	protein_coding	-0.761354148	3.08E-08	3.39E-07	Down
CD69	protein_coding	-1.123351352	3.09E-08	3.40E-07	Down
MYO3A	protein_coding	-1.205964672	3.12E-08	3.43E-07	Down
RPARP-AS1	long_non_coding	-0.661812898	3.13E-08	3.44E-07	Down
ANXA6	protein_coding	-0.602099532	3.15E-08	3.46E-07	Down
CFP	protein_coding	-0.977671324	3.15E-08	3.46E-07	Down

CSRNP3	protein_coding	-0.951492626	3.23E-08	3.54E-07	Down
PLCL1	protein_coding	-0.682934707	3.26E-08	3.57E-07	Down
MYH3	protein_coding	-0.61674406	3.31E-08	3.61E-07	Down
PHYHIPL	protein_coding	-1.153027544	3.43E-08	3.73E-07	Down
SERPINA6	protein_coding	-1.613026094	3.44E-08	3.74E-07	Down
PLP1	protein_coding	-1.382087851	3.49E-08	3.79E-07	Down
TMEM198	protein_coding	-0.764503094	3.50E-08	3.80E-07	Down
P2RY13	protein_coding	-0.967395446	3.50E-08	3.80E-07	Down
SV2B	protein_coding	-1.285084583	3.60E-08	3.88E-07	Down
MYEF2	protein_coding	-0.862122165	3.64E-08	3.92E-07	Down
KCNJ8	protein_coding	-0.840532305	3.65E-08	3.93E-07	Down
TTR	protein_coding	-2.129740068	3.68E-08	3.96E-07	Down
DNAH6	protein_coding	-0.739128442	4.02E-08	4.28E-07	Down
GYG2	protein_coding	-0.686969717	4.03E-08	4.28E-07	Down
CD180	protein_coding	-0.978009941	4.18E-08	4.43E-07	Down
SCD5	protein_coding	-0.777381558	4.42E-08	4.66E-07	Down
GABRB3	protein_coding	-1.219627539	4.51E-08	4.75E-07	Down
QPCT	protein_coding	-1.1140916	4.60E-08	4.83E-07	Down
LDB2	protein_coding	-0.613838208	4.72E-08	4.95E-07	Down
CD8A	protein_coding	-0.999463129	4.72E-08	4.95E-07	Down
VENTX	protein_coding	-0.771096426	4.81E-08	5.03E-07	Down
ID4	protein_coding	-0.717425265	4.95E-08	5.15E-07	Down
STARD9	protein_coding	-0.608823498	4.95E-08	5.15E-07	Down
LPL	protein_coding	-0.959913612	4.96E-08	5.16E-07	Down
LAT2	protein_coding	-0.679967316	4.97E-08	5.16E-07	Down
BCAM	protein_coding	-0.962352153	4.98E-08	5.17E-07	Down
ABCA3	protein_coding	-0.763407606	5.00E-08	5.19E-07	Down
BRSK1	protein_coding	-0.861572074	5.09E-08	5.27E-07	Down
ALPL	protein_coding	-0.750137499	5.09E-08	5.27E-07	Down
BHMT2	protein_coding	-0.731086556	5.11E-08	5.28E-07	Down
FMC1	protein_coding	-0.613631099	5.17E-08	5.34E-07	Down
CTTNBP2	protein_coding	-0.877467989	5.32E-08	5.47E-07	Down
TBC1D9	protein_coding	-0.587512668	5.40E-08	5.54E-07	Down
BCHE	protein_coding	-0.944068001	5.56E-08	5.69E-07	Down
CD72	protein_coding	-0.907887314	5.58E-08	5.71E-07	Down
TMEM74B	protein_coding	-1.051932949	5.60E-08	5.72E-07	Down
CCR5	protein_coding	-0.941947742	5.68E-08	5.81E-07	Down
RHOJ	protein_coding	-0.617511386	5.69E-08	5.81E-07	Down
SMARCD3	protein_coding	-0.588921506	5.85E-08	5.95E-07	Down
NYAP1	protein_coding	-1.073995494	5.89E-08	5.99E-07	Down
PCDHB4	protein_coding	-0.87416657	6.64E-08	6.66E-07	Down
GAMT	protein_coding	-1.038948273	6.67E-08	6.68E-07	Down
F8	protein_coding	-0.618939971	6.74E-08	6.75E-07	Down
GPR155	protein_coding	-0.606720175	6.78E-08	6.78E-07	Down
FAM105A	protein_coding	-0.850702066	6.84E-08	6.82E-07	Down
VTN	protein_coding	-1.263814796	6.96E-08	6.93E-07	Down
TMEM132C	protein_coding	-1.124465971	7.05E-08	7.02E-07	Down
SPRN	protein_coding	-0.610493356	7.12E-08	7.07E-07	Down
ITPR1	protein_coding	-0.664485523	7.18E-08	7.13E-07	Down
STK33	protein_coding	-0.854907277	7.24E-08	7.17E-07	Down
LTB	protein_coding	-1.180084752	7.30E-08	7.22E-07	Down
ZBTB16	protein_coding	-1.152332547	7.33E-08	7.24E-07	Down

SAMHD1	protein_coding	-0.651034167	7.60E-08	7.49E-07	Down
LRP3	protein_coding	-0.668857876	7.68E-08	7.56E-07	Down
NCKAP1L	protein_coding	-0.851769433	7.80E-08	7.66E-07	Down
TMEM100	protein_coding	-0.917572365	7.92E-08	7.77E-07	Down
SAMD11	protein_coding	-0.836125903	8.03E-08	7.86E-07	Down
CXorf21	protein_coding	-0.8329417	8.05E-08	7.88E-07	Down
MOB1B	protein_coding	-0.635806908	8.23E-08	8.04E-07	Down
MPPED2	protein_coding	-0.849068344	8.29E-08	8.09E-07	Down
RAI2	protein_coding	-0.631208665	8.41E-08	8.17E-07	Down
PDE8B	protein_coding	-0.742405443	8.45E-08	8.21E-07	Down
ARHGEF6	protein_coding	-0.691994748	8.51E-08	8.24E-07	Down
TMEM255A	protein_coding	-1.008300917	9.00E-08	8.69E-07	Down
C2orf40	protein_coding	-1.060073524	9.02E-08	8.70E-07	Down
CAPS2	protein_coding	-0.619342769	9.04E-08	8.70E-07	Down
ST6GALNAC3	protein_coding	-0.669861096	9.15E-08	8.81E-07	Down
SNRPN	protein_coding	-0.665552787	9.26E-08	8.90E-07	Down
PARVB	protein_coding	-0.761158664	9.51E-08	9.13E-07	Down
TLL1	protein_coding	-0.921483393	9.54E-08	9.15E-07	Down
C3orf14	protein_coding	-0.713604873	9.65E-08	9.25E-07	Down
ZNF737	protein_coding	-0.657295665	9.68E-08	9.27E-07	Down
TMEM156	protein_coding	-0.997933415	9.76E-08	9.33E-07	Down
SLA	protein_coding	-0.857465291	1.04E-07	9.86E-07	Down
ZDHHC11B	protein_coding	-1.029242814	1.09E-07	1.03E-06	Down
ST8SIA4	protein_coding	-0.728428438	1.10E-07	1.04E-06	Down
PTN	protein_coding	-0.996065772	1.10E-07	1.04E-06	Down
LGI4	protein_coding	-0.888373647	1.11E-07	1.05E-06	Down
AC145207.5	long_non_coding	-0.664184881	1.13E-07	1.06E-06	Down
MFNG	protein_coding	-0.586691691	1.13E-07	1.07E-06	Down
GMFG	protein_coding	-0.705729553	1.14E-07	1.07E-06	Down
BMP5	protein_coding	-1.425074578	1.17E-07	1.10E-06	Down
CASS4	protein_coding	-0.794569898	1.19E-07	1.11E-06	Down
KRBA1	protein_coding	-0.594896774	1.21E-07	1.13E-06	Down
CABP7	protein_coding	-1.391757597	1.21E-07	1.14E-06	Down
SLC25A45	protein_coding	-0.596196478	1.23E-07	1.15E-06	Down
IGLV7-46	IG	-1.448545161	1.25E-07	1.17E-06	Down
RAB36	protein_coding	-0.731634054	1.26E-07	1.17E-06	Down
CYTIP	protein_coding	-0.886369875	1.26E-07	1.17E-06	Down
ABAT	protein_coding	-0.994177753	1.26E-07	1.17E-06	Down
PLD6	protein_coding	-0.695735787	1.26E-07	1.18E-06	Down
CDH19	protein_coding	-1.310890913	1.27E-07	1.18E-06	Down
CYTH4	protein_coding	-0.682216882	1.34E-07	1.24E-06	Down
ARG2	protein_coding	-0.929346205	1.34E-07	1.24E-06	Down
CNRIP1	protein_coding	-0.600859139	1.38E-07	1.28E-06	Down
SEMA6D	protein_coding	-0.746774831	1.38E-07	1.28E-06	Down
MFAP4	protein_coding	-0.87678869	1.38E-07	1.28E-06	Down
KANK3	protein_coding	-0.687431079	1.39E-07	1.28E-06	Down
EBF3	protein_coding	-0.746078095	1.44E-07	1.32E-06	Down
NAALAD2	protein_coding	-0.602137511	1.46E-07	1.33E-06	Down
PRICKLE2	protein_coding	-0.609172232	1.46E-07	1.34E-06	Down
SERP2	protein_coding	-0.734040054	1.47E-07	1.34E-06	Down
RASSF4	protein_coding	-0.604739101	1.48E-07	1.34E-06	Down
PRKCQ	protein_coding	-0.860283713	1.51E-07	1.37E-06	Down

RAB37	protein_coding	-0.904444934	1.51E-07	1.38E-06	Down
CHST9	protein_coding	-1.32450547	1.52E-07	1.38E-06	Down
ATP8B2	protein_coding	-0.602662811	1.56E-07	1.42E-06	Down
ZFP28	protein_coding	-0.64083629	1.58E-07	1.43E-06	Down
GPR65	protein_coding	-0.768776779	1.62E-07	1.46E-06	Down
ASB9	protein_coding	-1.097450269	1.65E-07	1.48E-06	Down
FAM26F	protein_coding	-0.86743749	1.66E-07	1.49E-06	Down
MAP1B	protein_coding	-0.779387267	1.66E-07	1.49E-06	Down
MEF2C	protein_coding	-0.618037915	1.67E-07	1.50E-06	Down
DOCK2	protein_coding	-0.871243479	1.72E-07	1.54E-06	Down
BMP6	protein_coding	-0.829522229	1.80E-07	1.60E-06	Down
CBFA2T3	protein_coding	-0.915819255	1.81E-07	1.61E-06	Down
FKBP11	protein_coding	-0.812642482	1.81E-07	1.61E-06	Down
SDK2	protein_coding	-0.941612781	1.86E-07	1.65E-06	Down
GADD45G	protein_coding	-0.833742856	1.88E-07	1.66E-06	Down
PARVG	protein_coding	-0.785986921	1.90E-07	1.68E-06	Down
FAXC	protein_coding	-0.843265064	1.91E-07	1.69E-06	Down
PREX2	protein_coding	-0.712852498	1.93E-07	1.70E-06	Down
APBA1	protein_coding	-0.649014235	1.94E-07	1.71E-06	Down
TMTC1	protein_coding	-0.808399842	1.97E-07	1.73E-06	Down
ARVCF	protein_coding	-0.748761623	1.99E-07	1.74E-06	Down
HHEX	protein_coding	-0.789906401	1.99E-07	1.75E-06	Down
ABCB1	protein_coding	-1.043882645	2.02E-07	1.77E-06	Down
NLRP3	protein_coding	-0.77823674	2.02E-07	1.77E-06	Down
ADAM33	protein_coding	-0.845993377	2.04E-07	1.78E-06	Down
SV2A	protein_coding	-0.832467313	2.04E-07	1.78E-06	Down
VAV3	protein_coding	-0.896644125	2.06E-07	1.79E-06	Down
KIAA1456	protein_coding	-0.784570504	2.06E-07	1.79E-06	Down
SYN1	protein_coding	-1.001904311	2.06E-07	1.79E-06	Down
SNCA	protein_coding	-0.909235648	2.06E-07	1.79E-06	Down
RGMA	protein_coding	-0.916948102	2.08E-07	1.80E-06	Down
MMP19	protein_coding	-0.821927346	2.13E-07	1.84E-06	Down
SP140	protein_coding	-1.045223257	2.14E-07	1.86E-06	Down
PI16	protein_coding	-1.607003688	2.17E-07	1.87E-06	Down
ZNF154	protein_coding	-0.617451935	2.18E-07	1.88E-06	Down
S100B	protein_coding	-0.867123649	2.25E-07	1.94E-06	Down
DOCK8	protein_coding	-0.728580783	2.29E-07	1.97E-06	Down
IGHV4-59	IG	-1.419866323	2.30E-07	1.97E-06	Down
DPT	protein_coding	-1.113122172	2.30E-07	1.97E-06	Down
FLT3	protein_coding	-1.179656421	2.34E-07	2.00E-06	Down
C1orf162	protein_coding	-0.719169571	2.36E-07	2.02E-06	Down
SNHG9	long_non_coding	-0.832642951	2.40E-07	2.05E-06	Down
GATS	protein_coding	-0.66872324	2.43E-07	2.07E-06	Down
KCNK17	protein_coding	-1.075568923	2.46E-07	2.09E-06	Down
SNX20	protein_coding	-0.820705042	2.50E-07	2.13E-06	Down
EBF2	protein_coding	-0.955423362	2.50E-07	2.13E-06	Down
MZB1	protein_coding	-1.177190672	2.51E-07	2.13E-06	Down
ADGRD1	protein_coding	-0.79049601	2.57E-07	2.18E-06	Down
GAS2	protein_coding	-0.851123806	2.60E-07	2.20E-06	Down
FIGN	protein_coding	-0.658471192	2.75E-07	2.32E-06	Down
MAGEL2	protein_coding	-0.949185724	2.78E-07	2.34E-06	Down
CST7	protein_coding	-0.81827931	2.87E-07	2.40E-06	Down

NAPSB	pseudogene	-0.963068774	2.88E-07	2.41E-06	Down
CD38	protein_coding	-1.015022947	2.92E-07	2.44E-06	Down
CARMIL3	protein_coding	-0.916625452	2.94E-07	2.45E-06	Down
FGB	protein_coding	-1.695916976	2.96E-07	2.47E-06	Down
CD53	protein_coding	-0.8489918	3.07E-07	2.54E-06	Down
MAGI2	protein_coding	-0.682997117	3.09E-07	2.56E-06	Down
KCNIP2	protein_coding	-0.84930378	3.09E-07	2.56E-06	Down
TMEM61	protein_coding	-1.07544981	3.11E-07	2.57E-06	Down
ZNF497	protein_coding	-0.672500647	3.14E-07	2.59E-06	Down
PLA2G2A	protein_coding	-1.828719608	3.14E-07	2.60E-06	Down
GCNT4	protein_coding	-0.773821624	3.14E-07	2.60E-06	Down
A1CF	protein_coding	-1.172719816	3.16E-07	2.61E-06	Down
LEPR	protein_coding	-0.765606209	3.21E-07	2.65E-06	Down
NPM2	protein_coding	-0.998443637	3.26E-07	2.69E-06	Down
44256	protein_coding	-0.677478405	3.28E-07	2.69E-06	Down
SEMA6A	protein_coding	-0.73809935	3.28E-07	2.70E-06	Down
DIRAS3	protein_coding	-0.931834788	3.36E-07	2.75E-06	Down
LYL1	protein_coding	-0.713443263	3.41E-07	2.80E-06	Down
DOCK3	protein_coding	-0.993705922	3.43E-07	2.81E-06	Down
EDARADD	protein_coding	-1.033175181	3.47E-07	2.84E-06	Down
PSTPIP1	protein_coding	-0.73922225	3.60E-07	2.93E-06	Down
C1orf228	protein_coding	-0.814922085	3.63E-07	2.94E-06	Down
SESN3	protein_coding	-0.61149457	3.66E-07	2.97E-06	Down
SLC5A9	protein_coding	-0.896195135	3.68E-07	2.98E-06	Down
EBF4	protein_coding	-0.706431005	3.91E-07	3.14E-06	Down
PATL2	protein_coding	-0.83777247	4.10E-07	3.28E-06	Down
ADAMDEC1	protein_coding	-1.221078322	4.10E-07	3.28E-06	Down
FAM155B	protein_coding	-0.97307033	4.13E-07	3.30E-06	Down
PLEK	protein_coding	-0.854521005	4.19E-07	3.34E-06	Down
SPARCL1	protein_coding	-0.709832525	4.26E-07	3.39E-06	Down
GAP43	protein_coding	-1.033074441	4.29E-07	3.41E-06	Down
PARM1	protein_coding	-0.974160835	4.34E-07	3.44E-06	Down
ADRB2	protein_coding	-0.847325699	4.34E-07	3.44E-06	Down
ADAMTS3	protein_coding	-0.797300293	4.39E-07	3.47E-06	Down
ABHD14A	protein_coding	-0.634896872	4.42E-07	3.49E-06	Down
CCL4	protein_coding	-0.808229658	4.48E-07	3.54E-06	Down
KDR	protein_coding	-0.603522917	4.57E-07	3.59E-06	Down
GIPR	protein_coding	-1.112766737	4.57E-07	3.59E-06	Down
IGKV3-15	IG	-1.372388174	4.61E-07	3.62E-06	Down
TMEM86B	protein_coding	-0.892398689	4.72E-07	3.70E-06	Down
KLHL13	protein_coding	-0.726598985	4.74E-07	3.71E-06	Down
CLEC4E	protein_coding	-1.000673298	4.78E-07	3.74E-06	Down
C5	protein_coding	-0.768064738	4.83E-07	3.77E-06	Down
BVES	protein_coding	-0.885851949	4.90E-07	3.82E-06	Down
SLAMF6	protein_coding	-0.983161253	4.96E-07	3.87E-06	Down
NR4A3	protein_coding	-1.162298529	5.04E-07	3.92E-06	Down
EMID1	protein_coding	-0.613471155	5.08E-07	3.94E-06	Down
CES1	protein_coding	-1.095839223	5.35E-07	4.13E-06	Down
KIAA1644	protein_coding	-1.020526081	5.53E-07	4.26E-06	Down
GPIHBP1	protein_coding	-0.788309996	5.63E-07	4.33E-06	Down
MERTK	protein_coding	-0.635491886	5.81E-07	4.45E-06	Down
MS4A7	protein_coding	-0.812720677	5.90E-07	4.52E-06	Down

CCDC184	protein_coding	-0.969955209	6.11E-07	4.66E-06	Down
LPAR1	protein_coding	-0.624715948	6.16E-07	4.70E-06	Down
RNF157	protein_coding	-0.720355236	6.20E-07	4.73E-06	Down
SSR4	protein_coding	-0.670565831	6.41E-07	4.87E-06	Down
C17orf100	protein_coding	-0.587198826	6.55E-07	4.96E-06	Down
SORBS1	protein_coding	-0.767169437	6.58E-07	4.98E-06	Down
KCTD16	protein_coding	-0.934773074	6.60E-07	4.98E-06	Down
PLCXD3	protein_coding	-1.157710868	6.77E-07	5.10E-06	Down
PCDH17	protein_coding	-0.686911687	7.05E-07	5.29E-06	Down
NTRK2	protein_coding	-0.988737532	7.05E-07	5.29E-06	Down
IGLV3-25	IG	-1.460727328	7.08E-07	5.30E-06	Down
CACNA2D1	protein_coding	-0.699897099	7.09E-07	5.31E-06	Down
SYT5	protein_coding	-1.258955539	7.09E-07	5.31E-06	Down
SCN8A	protein_coding	-0.670882107	7.20E-07	5.38E-06	Down
BOC	protein_coding	-0.771881904	7.36E-07	5.48E-06	Down
PDCD1	protein_coding	-0.953241537	7.40E-07	5.50E-06	Down
CTNND2	protein_coding	-1.20836315	7.55E-07	5.61E-06	Down
STMN3	protein_coding	-0.757586854	7.56E-07	5.61E-06	Down
FAM159B	protein_coding	-1.494087612	7.66E-07	5.68E-06	Down
LILRB5	protein_coding	-0.918344069	7.79E-07	5.75E-06	Down
FAM46A	protein_coding	-0.612844699	7.94E-07	5.85E-06	Down
ADAMTSL3	protein_coding	-0.882325721	7.96E-07	5.86E-06	Down
HVCN1	protein_coding	-0.694296318	8.03E-07	5.90E-06	Down
SNHG19	long_non_coding	-0.892148104	8.04E-07	5.91E-06	Down
IGLV1-51	IG	-1.375863907	8.08E-07	5.93E-06	Down
SYNPO2	protein_coding	-0.889855291	8.11E-07	5.94E-06	Down
AC139530.1	long_non_coding	-0.647498461	8.21E-07	6.01E-06	Down
SRRM3	protein_coding	-0.749057202	8.24E-07	6.02E-06	Down
REC8	protein_coding	-0.871488585	8.35E-07	6.09E-06	Down
NES	protein_coding	-0.652183994	8.63E-07	6.27E-06	Down
KIF12	protein_coding	-0.997939777	8.83E-07	6.39E-06	Down
ATP8B4	protein_coding	-0.646226788	8.86E-07	6.41E-06	Down
RGS18	protein_coding	-0.85183637	9.18E-07	6.60E-06	Down
SYNM	protein_coding	-0.910757318	9.27E-07	6.65E-06	Down
AC104083.1	long_non_coding	-0.679913475	9.55E-07	6.82E-06	Down
GFRA1	protein_coding	-1.044633742	9.61E-07	6.86E-06	Down
CD1C	protein_coding	-1.007325597	9.79E-07	6.96E-06	Down
LINC01089	long_non_coding	-0.705674857	1.01E-06	7.18E-06	Down
MAGI2-AS3	long_non_coding	-0.633256269	1.03E-06	7.29E-06	Down
MDGA1	protein_coding	-0.613868939	1.07E-06	7.55E-06	Down
FAM171A2	protein_coding	-0.865011526	1.08E-06	7.64E-06	Down
RASL10B	protein_coding	-0.802737575	1.10E-06	7.72E-06	Down
SLC1A2	protein_coding	-1.317781515	1.10E-06	7.75E-06	Down
PDE7B	protein_coding	-0.723223978	1.10E-06	7.75E-06	Down
C1orf127	protein_coding	-1.276664347	1.11E-06	7.77E-06	Down
INMT	protein_coding	-0.655117176	1.19E-06	8.33E-06	Down
VLDLR	protein_coding	-0.648800831	1.20E-06	8.38E-06	Down
SNAI3	protein_coding	-0.636633775	1.22E-06	8.50E-06	Down
IGLV3-21	IG	-1.405015528	1.23E-06	8.54E-06	Down
FLRT2	protein_coding	-0.935762522	1.24E-06	8.58E-06	Down
ZNF204P	pseudogene	-0.590750705	1.25E-06	8.65E-06	Down
SPIB	protein_coding	-1.213766043	1.28E-06	8.84E-06	Down

TNFAIP8L3	protein_coding	-0.725261944	1.29E-06	8.89E-06	Down
ACSL1	protein_coding	-0.607969818	1.38E-06	9.52E-06	Down
BRSK2	protein_coding	-1.258090073	1.39E-06	9.54E-06	Down
TCAF2	protein_coding	-0.610896034	1.40E-06	9.63E-06	Down
USP27X-AS1	long_non_coding	-0.595368145	1.42E-06	9.74E-06	Down
NTN1	protein_coding	-0.9241407	1.43E-06	9.81E-06	Down
COL21A1	protein_coding	-0.8383943	1.49E-06	1.02E-05	Down
ZDHHC8P1	pseudogene	-1.162077515	1.51E-06	1.03E-05	Down
FAM47E	protein_coding	-0.697436977	1.58E-06	1.07E-05	Down
CD320	protein_coding	-0.607915729	1.58E-06	1.07E-05	Down
PGR	protein_coding	-0.866137754	1.63E-06	1.10E-05	Down
SPEG	protein_coding	-0.943945995	1.63E-06	1.11E-05	Down
IGHV3-49	IG	-1.32907615	1.65E-06	1.12E-05	Down
QRICH2	protein_coding	-0.589442289	1.67E-06	1.12E-05	Down
MPP6	protein_coding	-0.646185406	1.67E-06	1.13E-05	Down
SUSD4	protein_coding	-0.925320758	1.67E-06	1.13E-05	Down
LILRB1	protein_coding	-0.750480976	1.69E-06	1.14E-05	Down
IGFN1	protein_coding	-1.174068609	1.70E-06	1.14E-05	Down
IGHV1-2	IG	-1.462122639	1.73E-06	1.16E-05	Down
ABCB4	protein_coding	-0.752683149	1.74E-06	1.17E-05	Down
SNCAIP	protein_coding	-0.956041665	1.76E-06	1.18E-05	Down
SLC45A1	protein_coding	-0.681266907	1.77E-06	1.18E-05	Down
TSNAXIP1	protein_coding	-0.619851392	1.77E-06	1.19E-05	Down
CGNL1	protein_coding	-0.606657789	1.77E-06	1.19E-05	Down
TNFAIP8L2	protein_coding	-0.670204617	1.78E-06	1.19E-05	Down
RPS6KA6	protein_coding	-0.908205531	1.81E-06	1.21E-05	Down
IL6R	protein_coding	-0.660626657	1.81E-06	1.21E-05	Down
SCGB2A1	protein_coding	-1.212817644	1.88E-06	1.24E-05	Down
CD1E	protein_coding	-0.967433357	1.88E-06	1.25E-05	Down
ABCA6	protein_coding	-0.743681893	1.93E-06	1.28E-05	Down
MYO1F	protein_coding	-0.608216301	1.97E-06	1.30E-05	Down
GNA14	protein_coding	-0.673007857	1.99E-06	1.31E-05	Down
ENAM	protein_coding	-1.124679255	1.99E-06	1.31E-05	Down
DICER1-AS1	long_non_coding	-0.667443229	2.00E-06	1.31E-05	Down
DNAJC3-AS1	long_non_coding	-0.630981562	2.00E-06	1.32E-05	Down
RSPO3	protein_coding	-1.049733202	2.02E-06	1.33E-05	Down
PIFO	protein_coding	-0.88090017	2.02E-06	1.33E-05	Down
CTLA4	protein_coding	-0.899227163	2.05E-06	1.34E-05	Down
IGLV6-57	IG	-1.33677336	2.14E-06	1.40E-05	Down
MATK	protein_coding	-0.701163178	2.18E-06	1.42E-05	Down
SLC30A8	protein_coding	-1.664879846	2.20E-06	1.43E-05	Down
UBAP1L	protein_coding	-0.590179129	2.25E-06	1.46E-05	Down
DOK3	protein_coding	-0.620912449	2.28E-06	1.48E-05	Down
G6PC2	protein_coding	-1.738146498	2.31E-06	1.49E-05	Down
AC034236.2	long_non_coding	-0.609516023	2.35E-06	1.52E-05	Down
ZNF467	protein_coding	-0.780246232	2.36E-06	1.52E-05	Down
IL2RB	protein_coding	-0.733229396	2.38E-06	1.53E-05	Down
PPP2R3B	protein_coding	-0.599300078	2.40E-06	1.55E-05	Down
TRPC4	protein_coding	-0.733092992	2.45E-06	1.58E-05	Down
PROX1	protein_coding	-0.92320636	2.48E-06	1.59E-05	Down
GPR34	protein_coding	-0.802534502	2.49E-06	1.60E-05	Down
IGSF1	protein_coding	-1.063506397	2.54E-06	1.63E-05	Down

AC004803.1	long_non_coding	-0.589054133	2.55E-06	1.63E-05	Down
PRPH	protein_coding	-1.082739955	2.58E-06	1.65E-05	Down
IGLV8-61	IG	-1.426782298	2.58E-06	1.65E-05	Down
BANK1	protein_coding	-0.895841653	2.61E-06	1.67E-05	Down
PTP4A3	protein_coding	-0.650496578	2.64E-06	1.68E-05	Down
CDH22	protein_coding	-1.332447098	2.70E-06	1.72E-05	Down
CCDC40	protein_coding	-0.648489298	2.91E-06	1.83E-05	Down
LIPE	protein_coding	-0.708513398	2.91E-06	1.84E-05	Down
NEFH	protein_coding	-0.803073424	2.93E-06	1.84E-05	Down
PRG4	protein_coding	-0.909665019	3.01E-06	1.89E-05	Down
NUCB2	protein_coding	-0.684624871	3.15E-06	1.97E-05	Down
CEACAM21	protein_coding	-0.710695273	3.21E-06	2.01E-05	Down
ITGA8	protein_coding	-0.680341376	3.26E-06	2.03E-05	Down
PAPPA2	protein_coding	-1.356393539	3.32E-06	2.06E-05	Down
PLXNA4	protein_coding	-0.884633075	3.33E-06	2.06E-05	Down
PLA2G5	protein_coding	-0.812632928	3.51E-06	2.16E-05	Down
PRF1	protein_coding	-0.660487306	3.55E-06	2.18E-05	Down
NKG7	protein_coding	-0.748661177	3.60E-06	2.21E-05	Down
PTGER3	protein_coding	-0.77303353	3.64E-06	2.23E-05	Down
LRRK2	protein_coding	-0.73143819	3.68E-06	2.25E-05	Down
ANXA2R	protein_coding	-0.690610951	3.97E-06	2.42E-05	Down
LIN7B	protein_coding	-0.690753201	3.99E-06	2.42E-05	Down
PDZK1	protein_coding	-0.911123436	4.07E-06	2.47E-05	Down
AP004608.1	long_non_coding	-1.214211902	4.14E-06	2.51E-05	Down
NPTX2	protein_coding	-0.991856346	4.16E-06	2.52E-05	Down
ZNF366	protein_coding	-0.729102093	4.17E-06	2.52E-05	Down
MCOLN2	protein_coding	-0.816161545	4.24E-06	2.56E-05	Down
PTPN7	protein_coding	-0.770102003	4.35E-06	2.62E-05	Down
FOLR2	protein_coding	-0.800663798	4.36E-06	2.63E-05	Down
CADM4	protein_coding	-0.611746799	4.62E-06	2.76E-05	Down
TMEM38B	protein_coding	-0.596432861	4.69E-06	2.79E-05	Down
CR2	protein_coding	-1.942470049	4.70E-06	2.80E-05	Down
CD209	protein_coding	-0.887807139	4.78E-06	2.84E-05	Down
KIF26A	protein_coding	-0.739944174	4.87E-06	2.89E-05	Down
CA11	protein_coding	-0.622139365	4.92E-06	2.92E-05	Down
IGHV4-31	IG	-1.354380982	5.13E-06	3.02E-05	Down
CCL5	protein_coding	-0.762750063	5.22E-06	3.08E-05	Down
CLEC2D	protein_coding	-0.734877745	5.38E-06	3.16E-05	Down
FAIM2	protein_coding	-0.808005375	5.38E-06	3.16E-05	Down
EVI2B	protein_coding	-0.751167013	5.42E-06	3.17E-05	Down
PODXL2	protein_coding	-0.85150385	5.57E-06	3.25E-05	Down
CH25H	protein_coding	-0.758939433	5.66E-06	3.30E-05	Down
CALCRL	protein_coding	-0.638366735	5.72E-06	3.33E-05	Down
KLHDC9	protein_coding	-0.610186914	5.74E-06	3.34E-05	Down
BAALC	protein_coding	-0.69204781	5.77E-06	3.35E-05	Down
MS4A6A	protein_coding	-0.680912118	5.80E-06	3.37E-05	Down
SEC31B	protein_coding	-0.645618878	6.02E-06	3.49E-05	Down
DCLK1	protein_coding	-0.819814372	6.19E-06	3.57E-05	Down
EPHB1	protein_coding	-0.721151844	6.31E-06	3.63E-05	Down
ECHDC3	protein_coding	-0.830548263	6.47E-06	3.72E-05	Down
SAA1	protein_coding	-1.181086931	6.53E-06	3.75E-05	Down
CES4A	protein_coding	-0.769581257	6.70E-06	3.83E-05	Down

SLC38A11	protein_coding	-1.043510982	6.90E-06	3.93E-05	Down
ADAMTSL1	protein_coding	-0.706728685	7.04E-06	4.00E-05	Down
INPP5D	protein_coding	-0.65222594	7.08E-06	4.02E-05	Down
LRCH2	protein_coding	-0.625986661	7.16E-06	4.06E-05	Down
AC133065.6	TEC	-0.680377957	7.25E-06	4.11E-05	Down
ITGA4	protein_coding	-0.741470768	7.43E-06	4.20E-05	Down
MUM1L1	protein_coding	-0.96609001	7.83E-06	4.40E-05	Down
AC084018.1	long_non_coding	-0.635909893	7.88E-06	4.42E-05	Down
SPN	protein_coding	-0.726491595	7.96E-06	4.45E-05	Down
CYP1B1	protein_coding	-0.846123335	8.07E-06	4.50E-05	Down
GPR173	protein_coding	-0.671930115	8.34E-06	4.64E-05	Down
SMIM10L2A	protein_coding	-0.678803968	8.44E-06	4.69E-05	Down
SELENOP	protein_coding	-0.649184678	8.53E-06	4.74E-05	Down
IL6	protein_coding	-1.189737322	8.57E-06	4.76E-05	Down
PLD4	protein_coding	-0.743445079	8.62E-06	4.78E-05	Down
HGF	protein_coding	-0.707246248	8.69E-06	4.82E-05	Down
APBB1IP	protein_coding	-0.693327326	8.90E-06	4.92E-05	Down
LCP2	protein_coding	-0.602288764	8.90E-06	4.92E-05	Down
CFD	protein_coding	-0.855657108	9.59E-06	5.25E-05	Down
MGP	protein_coding	-0.704992613	9.83E-06	5.36E-05	Down
ST6GALNAC5	protein_coding	-0.861217529	1.01E-05	5.46E-05	Down
FAM20A	protein_coding	-0.683865159	1.03E-05	5.58E-05	Down
PCDH9	protein_coding	-0.754971373	1.04E-05	5.61E-05	Down
LY86	protein_coding	-0.69973758	1.19E-05	6.35E-05	Down
IGKV1-27	IG	-1.301198202	1.22E-05	6.47E-05	Down
AOX1	protein_coding	-0.979476453	1.26E-05	6.67E-05	Down
IAPP	protein_coding	-1.736878045	1.28E-05	6.79E-05	Down
NCF4	protein_coding	-0.595274447	1.36E-05	7.16E-05	Down
ESRRG	protein_coding	-0.858487774	1.38E-05	7.26E-05	Down
IGHV3-33	IG	-1.247567399	1.39E-05	7.27E-05	Down
SYT11	protein_coding	-0.677577407	1.41E-05	7.40E-05	Down
RAB3A	protein_coding	-0.618685062	1.42E-05	7.41E-05	Down
IGHGP	pseudogene	-1.199866605	1.43E-05	7.47E-05	Down
SYPL2	protein_coding	-0.591523096	1.49E-05	7.74E-05	Down
TLR7	protein_coding	-0.774354746	1.52E-05	7.89E-05	Down
B4GALNT4	protein_coding	-0.919282683	1.53E-05	7.92E-05	Down
PLEKHB1	protein_coding	-0.706054856	1.56E-05	8.05E-05	Down
AC090152.1	long_non_coding	-0.709256554	1.57E-05	8.07E-05	Down
TP53I13	protein_coding	-0.606289515	1.61E-05	8.27E-05	Down
SUSD3	protein_coding	-0.68670889	1.61E-05	8.27E-05	Down
CACNB4	protein_coding	-0.615194789	1.62E-05	8.32E-05	Down
IGHV3-74	IG	-1.171724812	1.66E-05	8.51E-05	Down
GLP2R	protein_coding	-0.800974929	1.69E-05	8.63E-05	Down
IL21R	protein_coding	-0.808910139	1.71E-05	8.75E-05	Down
GOLGA2P5	pseudogene	-0.591359067	1.74E-05	8.85E-05	Down
AIF1L	protein_coding	-0.639039236	1.77E-05	8.98E-05	Down
HMCN2	protein_coding	-0.763420285	1.86E-05	9.42E-05	Down
IGKV3-20	IG	-1.183565619	1.90E-05	9.59E-05	Down
SYT16	protein_coding	-1.098589673	1.98E-05	9.94E-05	Down
CTSW	protein_coding	-0.732537712	2.00E-05	0.000100252	Down
ABI3BP	protein_coding	-0.751054925	2.00E-05	0.000100252	Down
CYSLTR2	protein_coding	-0.667298971	2.01E-05	0.000100603	Down

PPFIA3	protein_coding	-0.717560888	2.02E-05	0.000101218	Down
LILRB2	protein_coding	-0.653359433	2.09E-05	0.000104249	Down
KLHDC8A	protein_coding	-0.684721312	2.12E-05	0.000105302	Down
CCDC96	protein_coding	-0.623084298	2.13E-05	0.000106136	Down
SERPINE2	protein_coding	-0.715880795	2.14E-05	0.000106454	Down
RADIL	protein_coding	-0.752914793	2.16E-05	0.000107344	Down
ELOVL2	protein_coding	-0.843483511	2.17E-05	0.000107823	Down
FAM189A1	protein_coding	-0.808086766	2.21E-05	0.000109708	Down
ZNF385D	protein_coding	-0.829005391	2.23E-05	0.000110633	Down
TUBB2B	protein_coding	-0.679401639	2.27E-05	0.000112045	Down
PLPPR4	protein_coding	-0.711919692	2.30E-05	0.000113322	Down
FXYD2	protein_coding	-1.108922709	2.33E-05	0.000114626	Down
HCST	protein_coding	-0.63016594	2.39E-05	0.000117348	Down
FAM198A	protein_coding	-0.668450528	2.39E-05	0.000117653	Down
SERPINF1	protein_coding	-0.654014755	2.40E-05	0.000117744	Down
IGHG2	IG	-1.12062427	2.48E-05	0.000121181	Down
ITIH5	protein_coding	-0.682898462	2.51E-05	0.000122909	Down
NPHS1	protein_coding	-1.211554521	2.52E-05	0.00012315	Down
LRFN1	protein_coding	-0.693023602	2.57E-05	0.000125385	Down
PKNOX2	protein_coding	-0.646179326	2.65E-05	0.000128933	Down
CIITA	protein_coding	-0.611020889	2.71E-05	0.000131392	Down
KNDC1	protein_coding	-0.966889156	2.74E-05	0.000132826	Down
LINC00987	long_non_coding	-0.713504704	2.78E-05	0.000134627	Down
CD8B	protein_coding	-0.785037232	2.82E-05	0.000135959	Down
TNFRSF19	protein_coding	-0.606061924	2.86E-05	0.000137829	Down
PRR29	protein_coding	-0.620593721	2.91E-05	0.000140096	Down
DNAH7	protein_coding	-0.609827288	3.03E-05	0.000144921	Down
ZDBF2	protein_coding	-0.601295754	3.06E-05	0.000146547	Down
KMO	protein_coding	-0.631597446	3.08E-05	0.000147365	Down
SST	protein_coding	-1.247252697	3.10E-05	0.000147849	Down
ATP8A2	protein_coding	-0.641514969	3.14E-05	0.00014947	Down
RYR2	protein_coding	-0.714228545	3.16E-05	0.000150364	Down
SSPO	protein_coding	-0.739440401	3.23E-05	0.000153339	Down
AKAP7	protein_coding	-0.763131398	3.24E-05	0.000153488	Down
SERPINA1	protein_coding	-0.891039644	3.62E-05	0.000169382	Down
GREM2	protein_coding	-1.074519281	3.66E-05	0.00017067	Down
TM6SF1	protein_coding	-0.586186882	3.70E-05	0.000172502	Down
LILRA5	protein_coding	-0.70609162	3.70E-05	0.000172502	Down
ADCY2	protein_coding	-0.754217695	3.73E-05	0.00017368	Down
VWDE	protein_coding	-1.016952453	3.92E-05	0.000181738	Down
RGS17	protein_coding	-0.602033865	3.96E-05	0.000183495	Down
FHL5	protein_coding	-0.676625879	4.10E-05	0.000189332	Down
KLRD1	protein_coding	-0.644305403	4.14E-05	0.000191111	Down
RNFT2	protein_coding	-0.618259797	4.23E-05	0.000194755	Down
CYP39A1	protein_coding	-0.618655409	4.30E-05	0.00019765	Down
CSF1R	protein_coding	-0.592749744	4.55E-05	0.000207524	Down
CPNE5	protein_coding	-0.588943825	4.59E-05	0.000209057	Down
IGKV2-24	IG	-1.148928936	4.60E-05	0.00020953	Down
ADAM23	protein_coding	-0.6315213	4.75E-05	0.000215791	Down
KIRREL2	protein_coding	-1.167692624	4.94E-05	0.000223012	Down
IGLV10-54	IG	-1.38269832	4.98E-05	0.00022469	Down
KCNG1	protein_coding	-0.859522643	5.13E-05	0.000230857	Down

JSRP1	protein_coding	-0.931769748	5.18E-05	0.000232662	Down
ADAMTS8	protein_coding	-0.959927708	5.39E-05	0.000241359	Down
ADAMTS16	protein_coding	-0.925803297	5.45E-05	0.000243462	Down
SYNGR3	protein_coding	-0.683321188	5.45E-05	0.000243492	Down
DAAM2	protein_coding	-0.668052047	5.49E-05	0.000245141	Down
PLN	protein_coding	-0.904762786	5.56E-05	0.000247675	Down
EVI2A	protein_coding	-0.631657547	5.69E-05	0.00025322	Down
FCER1A	protein_coding	-0.842580124	5.71E-05	0.000253954	Down
KCNK2	protein_coding	-0.832077127	5.73E-05	0.000254144	Down
COL23A1	protein_coding	-0.587481754	5.80E-05	0.000257022	Down
SVEP1	protein_coding	-0.700055761	5.90E-05	0.000261432	Down
SLC1A7	protein_coding	-0.672705569	6.23E-05	0.000274105	Down
NRG1	protein_coding	-0.728827503	6.54E-05	0.000286465	Down
CPS1	protein_coding	-1.002786351	6.54E-05	0.000286465	Down
KCNAB1	protein_coding	-0.606135413	6.62E-05	0.000289193	Down
IL1RL1	protein_coding	-0.809867215	6.63E-05	0.000289594	Down
MBNL1-AS1	long_non_coding	-0.693015785	6.64E-05	0.000289878	Down
C3orf70	protein_coding	-0.610252461	6.65E-05	0.000290309	Down
TMEM236	protein_coding	-0.804649786	6.73E-05	0.000292673	Down
ANKRD33B	protein_coding	-0.616879949	6.96E-05	0.000302099	Down
MYO1G	protein_coding	-0.608664641	7.15E-05	0.000309564	Down
FYB1	protein_coding	-0.684670479	7.46E-05	0.000321163	Down
IGLV1-44	IG	-1.094287379	7.55E-05	0.000324591	Down
TIMP4	protein_coding	-0.759405214	7.62E-05	0.00032695	Down
NKX2-3	protein_coding	-0.641087258	7.65E-05	0.000328158	Down
TENM2	protein_coding	-0.779413479	7.84E-05	0.000334795	Down
KCNC3	protein_coding	-0.649567922	8.13E-05	0.000345866	Down
CCL4L2	protein_coding	-0.732480133	8.28E-05	0.000351602	Down
APLNR	protein_coding	-0.616963589	8.39E-05	0.000355708	Down
THBS4	protein_coding	-0.81064242	8.46E-05	0.00035802	Down
CDK5R1	protein_coding	-0.691439931	8.48E-05	0.000358595	Down
IGKC	IG	-1.038875232	8.58E-05	0.000362349	Down
KLF15	protein_coding	-0.72945313	8.58E-05	0.000362349	Down
IGKV3D-20	IG	-1.204508324	8.70E-05	0.000366676	Down
TWIST2	protein_coding	-0.605015221	8.75E-05	0.000368188	Down
JCHAIN	protein_coding	-1.067536673	8.80E-05	0.000369995	Down
IGHV3-15	IG	-1.072850368	8.82E-05	0.000370787	Down
WT1	protein_coding	-0.895484652	8.87E-05	0.000372522	Down
SLC18A2	protein_coding	-0.683753086	9.01E-05	0.00037778	Down
FPR1	protein_coding	-0.666263798	9.21E-05	0.000385103	Down
ADAMTSL2	protein_coding	-0.586525637	9.25E-05	0.000386557	Down
SAMSN1	protein_coding	-0.630846232	9.36E-05	0.000390472	Down
BTNL9	protein_coding	-0.604501206	9.51E-05	0.000396119	Down
PDE6B	protein_coding	-0.633828611	9.65E-05	0.000401287	Down
SLIT2	protein_coding	-0.642993104	9.67E-05	0.00040164	Down
WNK2	protein_coding	-0.811726722	0.000101697	0.000419074	Down
GPR183	protein_coding	-0.70009529	0.000102628	0.000422047	Down
LMOD1	protein_coding	-0.747254227	0.000105135	0.000431602	Down
DCDC2	protein_coding	-0.800252476	0.000106041	0.000435006	Down
IGLV7-43	IG	-1.111264263	0.000106172	0.000435272	Down
MEOX2	protein_coding	-0.666993339	0.000115432	0.000467301	Down
HLA-DOA	protein_coding	-0.637034829	0.000117221	0.000473811	Down

GALNT17	protein_coding	-0.596650482	0.000124363	0.00049788	Down
GRB14	protein_coding	-0.693674209	0.000124426	0.000497998	Down
CKB	protein_coding	-0.675256498	0.000125127	0.000500278	Down
IGLV3-1	IG	-1.097809059	0.000126439	0.000504591	Down
HNF1A-AS1	long_non_coding	-0.783055166	0.0001265	0.000504703	Down
CX3CR1	protein_coding	-0.734206144	0.000126716	0.000505032	Down
OLFM2	protein_coding	-0.608209986	0.000127285	0.000507036	Down
TMEM119	protein_coding	-0.609468503	0.00012748	0.000507679	Down
GREB1	protein_coding	-0.605244274	0.000128019	0.000509558	Down
PRR36	protein_coding	-0.759405578	0.00012872	0.000511811	Down
CMKLR1	protein_coding	-0.622368076	0.000129346	0.00051403	Down
FRMPD3	protein_coding	-0.75579974	0.000130341	0.000517142	Down
MS4A4A	protein_coding	-0.64761606	0.000130549	0.000517727	Down
44442	protein_coding	-0.688486254	0.000131249	0.000520094	Down
CHRNA3	protein_coding	-0.851542614	0.000131835	0.00052228	Down
IGLC3	IG	-1.000349019	0.000132643	0.000524936	Down
FMO2	protein_coding	-0.732107167	0.000133117	0.000526123	Down
NRN1	protein_coding	-0.674763981	0.000133926	0.000528672	Down
KCNJ2	protein_coding	-0.608836182	0.000135147	0.00053276	Down
P2RY1	protein_coding	-0.66714992	0.000135662	0.000534652	Down
VIP	protein_coding	-1.13736442	0.000136849	0.000538549	Down
DHRS2	protein_coding	-0.920292426	0.000137497	0.000540479	Down
TFEC	protein_coding	-0.658487172	0.000140039	0.000549049	Down
ADH6	protein_coding	-0.806073074	0.000145942	0.000568377	Down
SIGLEC8	protein_coding	-0.7679391	0.000152877	0.000592952	Down
GHRL	protein_coding	-0.930405155	0.000154738	0.000598945	Down
AQP1	protein_coding	-0.60534449	0.000155125	0.000600288	Down
IGLV3-10	IG	-1.187120912	0.000157878	0.000609994	Down
IGKV3-11	IG	-0.991245883	0.000157914	0.000609994	Down
SBSPON	protein_coding	-0.637923702	0.000166973	0.000641069	Down
CRYAB	protein_coding	-0.621089104	0.000172307	0.000658219	Down
TPTEP1	pseudogene	-0.61317034	0.000177696	0.000676932	Down
FGL2	protein_coding	-0.636569863	0.000192337	0.000724702	Down
DLK1	protein_coding	-1.233663895	0.000197617	0.000742899	Down
IGHV3-72	IG	-1.055691697	0.000198684	0.000745953	Down
SFRP4	protein_coding	-0.736897835	0.000204489	0.000763665	Down
ALDH1A1	protein_coding	-0.639730949	0.000208157	0.000776024	Down
TMEM27	protein_coding	-0.730639378	0.000209963	0.000781981	Down
JAML	protein_coding	-0.611906666	0.000218665	0.000810817	Down
CRISP3	protein_coding	-1.416969619	0.000221208	0.000819049	Down
MRGPRF	protein_coding	-0.629467605	0.000222491	0.000822993	Down
SLC2A2	protein_coding	-1.057162452	0.000223988	0.000828329	Down
RGN	protein_coding	-0.69354568	0.000224567	0.000829805	Down
NEXN	protein_coding	-0.623920806	0.000227177	0.000838692	Down
CDX2	protein_coding	-0.829868189	0.000229861	0.000847158	Down
CFAP43	protein_coding	-0.611118664	0.000245013	0.000896687	Down
PNMA2	protein_coding	-0.672183569	0.000246682	0.00090236	Down
EDN3	protein_coding	-0.90766475	0.000269974	0.000975803	Down
IGKV1-5	IG	-1.005469084	0.000270669	0.000977617	Down
AP000892.3	TEC	-0.669828285	0.000276864	0.000996436	Down
LCN12	protein_coding	-0.682578941	0.000280636	0.001007146	Down
MRAP2	protein_coding	-0.648568866	0.000288302	0.001029544	Down

PEG10	protein_coding	-0.801905347	0.000293948	0.00104724	Down
SMOC1	protein_coding	-1.04572747	0.000305702	0.001082918	Down
BNIP3	protein_coding	-0.606775069	0.000308019	0.001089696	Down
GFI1	protein_coding	-0.636105772	0.000308788	0.001091909	Down
GZMA	protein_coding	-0.593566692	0.000316403	0.00111339	Down
AC110285.2	long_non_coding	-0.714465201	0.000318178	0.001119375	Down
CCDC80	protein_coding	-0.659278684	0.000321862	0.001131026	Down
FBLN1	protein_coding	-0.61978749	0.000324476	0.001139202	Down
MAATS1	protein_coding	-0.600668031	0.000324489	0.001139202	Down
IGSF21	protein_coding	-0.590998958	0.000327608	0.00114751	Down
S1PR4	protein_coding	-0.644745445	0.000329876	0.001154375	Down
PIK3CG	protein_coding	-0.627406418	0.000333552	0.001166165	Down
IGLV2-11	IG	-0.984560142	0.00034718	0.001206585	Down
IGLC2	IG	-0.910416405	0.000352222	0.00122103	Down
IGHV3-11	IG	-1.038351513	0.000358164	0.001239366	Down
UGT2B15	protein_coding	-1.065075767	0.000359849	0.00124463	Down
MEOX1	protein_coding	-0.645926853	0.000360958	0.001247612	Down
DNAJC12	protein_coding	-0.695753111	0.000373501	0.001285987	Down
IGHV2-26	IG	-1.127157323	0.000386528	0.00132393	Down
IGLV1-40	IG	-0.978112453	0.000395374	0.001350574	Down
STC2	protein_coding	-0.621210376	0.00039843	0.001360397	Down
IGLV3-19	IG	-1.034942171	0.00040159	0.00136944	Down
CNN1	protein_coding	-0.674206685	0.000433673	0.001465565	Down
ADRA2C	protein_coding	-0.653535251	0.000438514	0.00147962	Down
TMEM132B	protein_coding	-0.5976024	0.000440761	0.001486541	Down
IGHV1-46	IG	-0.972454522	0.000441713	0.00148909	Down
MIAT	long_non_coding	-0.684082478	0.000450407	0.00151534	Down
IGLV3-9	IG	-1.094128215	0.000460817	0.001546619	Down
CCL3	protein_coding	-0.599799147	0.000462783	0.00155219	Down
SIGLEC10	protein_coding	-0.598053144	0.000467987	0.001567567	Down
DACH1	protein_coding	-0.690815388	0.000478423	0.001599344	Down
C3	protein_coding	-0.631340584	0.0004998	0.001661657	Down
REEP1	protein_coding	-0.674230025	0.000516381	0.001710412	Down
GPR27	protein_coding	-0.653370487	0.000524103	0.001732966	Down
S100A1	protein_coding	-0.647249418	0.000537538	0.001770927	Down
TMEM130	protein_coding	-0.711764586	0.000540201	0.00177806	Down
IGLV2-14	IG	-0.96608517	0.000545358	0.001792312	Down
CSF3R	protein_coding	-0.596865854	0.00063884	0.002062033	Down
SLC16A9	protein_coding	-0.640603019	0.000644502	0.002077658	Down
CD84	protein_coding	-0.59876536	0.000646674	0.002082447	Down
CRYBA2	protein_coding	-1.10224284	0.000656008	0.002108225	Down
DPYSL4	protein_coding	-0.593571173	0.000706636	0.002250708	Down
PDZD2	protein_coding	-0.596556017	0.00071048	0.002260576	Down
IGHG4	IG	-1.201416107	0.000726383	0.002305379	Down
GUCY2C	protein_coding	-0.828584692	0.00074453	0.002354428	Down
IGKV1-9	IG	-0.966093569	0.000765838	0.002413441	Down
IGHA1	IG	-0.860002884	0.000769523	0.002422538	Down
RAB3B	protein_coding	-0.625594859	0.000805679	0.002516525	Down
IGHG3	IG	-0.987993848	0.000807766	0.002522004	Down
HOGA1	protein_coding	-0.80486408	0.000814079	0.002537017	Down
C6	protein_coding	-0.935814591	0.000829313	0.002581312	Down
IGHV4-39	IG	-0.958432892	0.000875398	0.002704788	Down

PAIP2B	protein_coding	-0.7222468	0.000883174	0.002726038	Down
MAMDC2	protein_coding	-0.609523955	0.000916877	0.002814611	Down
SGCA	protein_coding	-0.624583214	0.000921158	0.002825468	Down
SLC17A4	protein_coding	-0.918700271	0.000937891	0.002868676	Down
NMNAT2	protein_coding	-0.640175058	0.000977304	0.002976629	Down
ECEL1	protein_coding	-0.65243142	0.00101491	0.003078196	Down
SSTR5-AS1	long_non_coding	-0.966871875	0.001074972	0.003231303	Down
MFSD6L	protein_coding	-0.632221869	0.001137917	0.003397631	Down
CPLX1	protein_coding	-0.585836128	0.001160459	0.0034561	Down
GFRA3	protein_coding	-0.726968027	0.001212152	0.003591727	Down
IGHG1	IG	-0.906909916	0.001260948	0.003716722	Down
IGLV1-47	IG	-0.879895574	0.001291294	0.003792911	Down
SIGLEC14	protein_coding	-0.755108693	0.001403522	0.004075235	Down
IGKV1-6	IG	-0.864680991	0.001583096	0.004535899	Down
HS3ST2	protein_coding	-0.672912202	0.001642214	0.004687583	Down
IGHV3-21	IG	-0.857846424	0.001681721	0.004783262	Down
IGHV4-34	IG	-0.868749237	0.001713799	0.004857204	Down
AK5	protein_coding	-0.690699097	0.00171853	0.004868795	Down
SMIM6	protein_coding	-0.624057039	0.001725948	0.004888897	Down
BTBD11	protein_coding	-0.656808778	0.001801991	0.005077762	Down
SAA2	protein_coding	-0.822802476	0.001813181	0.005104553	Down
GPR37	protein_coding	-0.591557763	0.001860878	0.005218504	Down
PAH	protein_coding	-0.98551818	0.001952346	0.005438833	Down
MS4A8	protein_coding	-0.825261402	0.001989662	0.005530606	Down
COL28A1	protein_coding	-0.696403657	0.001999963	0.005557202	Down
PPY	protein_coding	-1.379655682	0.002075725	0.005745629	Down
VNN2	protein_coding	-0.609118464	0.00222392	0.006107933	Down
TMEM151A	protein_coding	-0.759209821	0.002457071	0.006673401	Down
C16orf54	protein_coding	-0.703075979	0.002513992	0.006806076	Down
IGHV5-51	IG	-0.847247	0.002725641	0.007322899	Down
TRBC2	TR	-0.589501064	0.002802159	0.007493726	Down
IGHA2	IG	-0.816948422	0.002802938	0.007493726	Down
EEF1A2	protein_coding	-0.765638485	0.002805831	0.00749852	Down
CNTN1	protein_coding	-0.592784429	0.0029011	0.007713735	Down
IGKV4-1	IG	-0.829275899	0.002931514	0.007785055	Down
CCL11	protein_coding	-0.59560376	0.002970708	0.007873984	Down
CLDN3	protein_coding	-0.706150012	0.003015735	0.007975225	Down
KCNJ16	protein_coding	-0.87459914	0.003047416	0.008050589	Down
MASP1	protein_coding	-0.706683766	0.003177066	0.008358179	Down
LRRC55	protein_coding	-0.611558578	0.003889762	0.009955299	Down
IGHV3-23	IG	-0.812603182	0.004095278	0.01042853	Down
INS	protein_coding	-1.148598259	0.004140441	0.010519644	Down
HBA2	protein_coding	-0.812086004	0.004319847	0.010930697	Down
ALKAL2	protein_coding	-0.771039211	0.00446683	0.011251964	Down
IGHV1-24	IG	-0.851354592	0.004496735	0.011319779	Down
DPEP1	protein_coding	-0.706266814	0.004575341	0.011490973	Down
FGF7	protein_coding	-0.607756546	0.004875278	0.012139802	Down
IGHV3-30	IG	-0.851619106	0.005117442	0.012667984	Down
LGALS2	protein_coding	-0.668891273	0.005122947	0.012679545	Down
SSTR1	protein_coding	-0.697950483	0.005147826	0.012730741	Down
PTPRZ1	protein_coding	-0.612241535	0.005156645	0.012750472	Down
CHRM3	protein_coding	-0.636332787	0.005260416	0.01297535	Down

HPN	protein_coding	-0.713720056	0.005393898	0.013259344	Down
TMED6	protein_coding	-0.654533396	0.005873882	0.014261436	Down
IGFALS	protein_coding	-0.619149425	0.005912686	0.014339538	Down
SLC4A4	protein_coding	-0.703390158	0.00694551	0.016452922	Down
IGHV3-53	IG	-0.732200593	0.007236262	0.01703766	Down
ACE2	protein_coding	-0.693012173	0.008073519	0.018695728	Down
F11	protein_coding	-0.749497168	0.008119359	0.018787546	Down
ANPEP	protein_coding	-0.697166709	0.008222619	0.01900041	Down
DACT2	protein_coding	-0.610896359	0.00893084	0.02042546	Down
CEACAM7	protein_coding	-0.846394808	0.009586444	0.021747902	Down
ACTG2	protein_coding	-0.66121485	0.010075139	0.022724088	Down
WSCD2	protein_coding	-0.603319309	0.010196395	0.022963449	Down
IGLL5	protein_coding	-0.704470085	0.010475468	0.023494336	Down
CILP	protein_coding	-0.651757315	0.010583022	0.023714532	Down
GCG	protein_coding	-1.097382742	0.010951265	0.02442071	Down
IGLV2-8	IG	-0.683881077	0.014213346	0.030515766	Down
CCL17	protein_coding	-0.642586582	0.015600542	0.033068547	Down
IGLV2-23	IG	-0.65074879	0.016011509	0.033811975	Down
IGHV1-18	IG	-0.720023977	0.01666084	0.034993298	Down
HBB	protein_coding	-0.657760492	0.017170921	0.035871034	Down
SLC38A3	protein_coding	-0.710529329	0.020981945	0.04269245	Down
AC245369.3	IG	-0.662445807	0.022407912	0.045260165	Down
APOBEC2	protein_coding	-0.616796988	0.024973279	0.049707469	Down
CFTR	protein_coding	-0.587317343	0.029023781	0.05640127	Down
REG3G	protein_coding	-1.108283598	0.032927028	0.062741181	Down
ERP27	protein_coding	-0.64766246	0.033728157	0.064018539	Down
IGKV1-17	IG	-0.605747442	0.03648227	0.06844146	Down
CELA3B	protein_coding	-1.063572574	0.046211123	0.083742831	Down
